# Synthesis of *N*-Heteroarenemethyl Esters via C–C Bond Cleavage of Acyl Cyanides Under Transition Metal-Free Conditions

**DOI:** 10.3389/fchem.2021.822625

**Published:** 2022-01-27

**Authors:** Miao Lai, Fangyao Su, Jingyi Hu, Mengzhuo Wang, Mingqin Zhao, Ganlin Zhang

**Affiliations:** Flavors and Fragrance Engineering and Technology Research Center of Henan Province, College of Tobacco Science, Henan Agricultural University, Zhengzhou, China

**Keywords:** N-heteroaryl esters, C-C bond cleavage, acyl cyanides, transition-metal free synthesis, N-heteroaryl methanols

## Abstract

A practical method to synthesize *N*-heteroaryl esters from *N*-heteroaryl methanols with acyl cyanides *via* C–C bond cleavage without using any transition metal is demonstrated here. The use of Na_2_CO_3_/15-crown-5 couple enables access to a series of *N*-heteroaryl esters in high efficiency. This protocol is operationally simple and highly environmentally benign producing only cyanides as byproducts.

## Introduction

**SCHEME 1:**
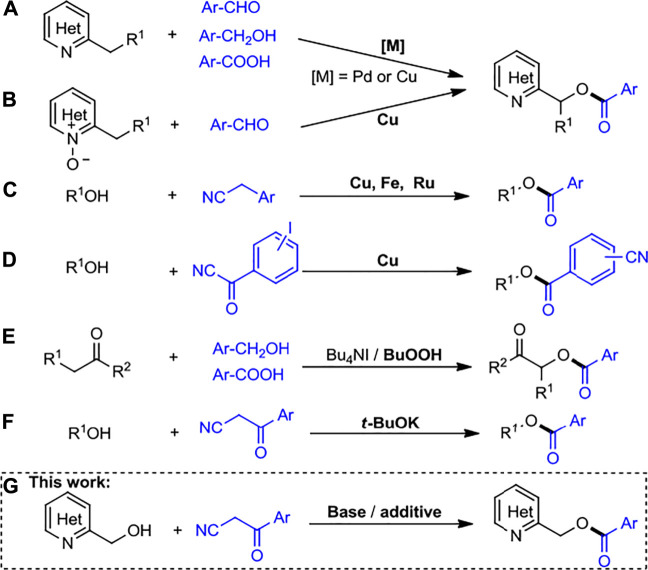
Strategies toward the synthesis of esterification products.

Heteroaryl esters and their derivatives could serve as interesting building blocks for the preparation of various functionalized products including bioactive natural products, pharmaceuticals, dyes, and flavors (Otera, 2010; Trotier Faurion et al., 2013; Armani et al., 2014; Liu B. et al., 2015; Xu et al., 2018; Bayout et al., 2020; Xu et al., 2022). Therefore, methodologies for the synthesis of these molecular architectures have experienced huge developments in recent years. The conventional syntheses include interesterification and oxidative carbonylation of ethers (Zhao et al., 2014; Lu et al., 2015), and the reactions of alcohols with methanol (Zhang and Wang, 2019), carboxylic acids (Teruaki et al., 2003; Saeed et al., 2008), aldehydes (Tang et al., 2014; Huang et al., 2016; Chun and Chung, 2017), ketones (Huang et al., 2014; Rammurthy et al., 2021), aliphatic amides (Hie et al., 2016; Bourne-Branchu et al., 2017), carbonates (Chen et al., 2014), and acid halides (Tamaddon et al., 2005; Akhlaghinia et al., 2010), respectively. In the last decade, direct activation and functionalization of the C–H bond has emerged as a powerful method in the field of organic synthesis and witnessed significant progress (McMurray et al., 2011; Huang et al., 2012; Wencel-Delord and Glorius, 2013; Chen et al., 2015; Liu C. et al., 2015; Dong et al., 2017). For example, successful benzylic C (sp^3^)-H acyloxylations of alkyl *N*-heteroarenes were achieved using simple aldehydes and acids *via* a copper or palladium catalysis (Scheme 1A) (Jiang et al., 2010; Chen et al., 2018; Cheng et al., 2019). Moreover, Soulé and coworkers achieved oxidative esterification of aldehydes with prefunctionalized 2-alkylheterocycle *N*-oxides *via* copper catalysis (Scheme 1B) (Wang et al., 2017).

In recent years, the process of carbon–carbon (C–C) bond cleavage of ketones under transition metal (Fe, Cu, or Ru) catalysis has provided various ester compounds (Yan et al., 2014; Huang et al., 2016; Arzumanyan, 2017).

Recently, a simple and direct aerobic oxidative esterification reaction of arylacetonitriles with alcohols/phenols is achieved in the presence of a copper salt and molecular oxygen (Dong et al., 2021). On the other hand, Song and Plietker groups reported aerobic oxidative C–CN bond cleavage of arylacetonitriles leading to various esters with catalysis of Fe and Ru, respectively (Scheme 1C) (Kong et al., 2016; Eisele et al., 2019). In general, ester compounds could be achieved using acylcyanides as acylating agents. Cu-catalyzed esterification using acyl cyanides with alcohols to yield the corresponding cyano-substituted esters is also reported (Scheme 1D) (Chen et al., 2020).

Furthermore, esterification reactions could proceed using photochemical strategies, in which reaction mechanisms involve mainly single electron transfer, energy transfer, or other radical procedures (Deng et al., 2021). A novel and metal-free method for the synthesis of α-ketoesters from β-ketonitriles and alcohols was reported, but under visible light irradiation conditions (Xu et al., 2018). Besides, the acyloxycarbonyl compounds could be obtained by TBAI-catalyzed acyloxylation of ketones (Uyanik et al., 2011; Guo et al., 2014), which the excess TBHP was required for the reaction process (Scheme 1E). Very recently, Subaramannian et al. reported *t*-BuOK catalyzed esterification using acyl cyanides with alcohols (Scheme 1F); however, their applicability was limited to *N*-heteroaryl methanols (Subaramanian et al., 2020). In addition, transition metal-free activation of amides by cleavage of C–N bond to obtain the aryl esters is well known (Li et al., 2018; Li and Szostak, 2020). Therefore, these reported esterification protocols involve the metal catalysts, lack of step efficiency, conditions of light irradiation, limited substrate scope of *N*-heterocyclic compounds, and need of oxidants. In our continuous effort in the construction of *N*-heteroaryl compounds (Lai et al., 2018; Zhai et al., 2018), we disclose herein an efficient and new protocol for the base-promoted esterification of *N*-heteroaryl methanols via C–C bond cleavage of arylacetonitriles acyl cyanides as acylating sources (Scheme 1G). The present protocol is simple to handle and does not involve any metal catalyst detrimental to environmental safety.

## Result and discussion

Our initial optimization using 2-pyrazinylmethanol *1a* and benzoylacetonitrile *2a* as the model substrates revealed that the reaction proceeded as anticipated with 1 equiv of *t*-BuOK in toluene according to the reported conditions (Roy et al., 2019; Subaramanian et al., 2020), affording the desired product *3a* in 10% yield (Table 1, entry 1). No product was detected in the absence of base (Table 1, entry 2), which suggests that it played a crucial role. Further screening of other bases indicated that Na_2_CO_3_ was obviously superior to the others, providing the desired product *3a* in 25% isolated yield (Table 1, entries 3–9 vs. entry 1). Interestingly, an improved yield (61%) was obtained when the reaction was conducted in the presence of 15-crown-5 (Table 1, entry 10).The role of crown ether in the reaction is increasing the solubility of base in solvent and known to be effective for trapping the potassium and sodium ions (Liotta et al., 1974; Lutz et al., 1988; Hay et al., 1993; Reuter et al., 1999). Subsequently, the evaluation of additives was conducted, and the results indicated that none of the screened additives (PPh_3_, 1,10-Phen, and TMEDA) benefited the outcome (29%–32%, Table 1, entries 11–13 vs. 10). Thus, 15-crown-5 was selected as the additive to assess the effect of solvents, including 1,4-dioxane, DMF, DMSO, DCE, and THF (Table 1, entries 14–18). All of the attempts did not show any improvement on the reaction yield compared with toluene (Table 1, entry 10). Then, we explored the other reaction parameters including the reaction temperature, the mol ratio of *1a* and *2a*, and the amount of additive. The yield was not much improved compared with the yield of 140°C (51%) when the reaction was conducted at 150°C (62%, Table 1, entry 20 vs. 10). Therefore, the reaction temperature remains unchanged. Gratifyingly, when the mole ratio of *1a* and *2a* was modified from 1:1 to 2.5:1, the reaction provided the best yield (85%) of *3a* (Table 1, entry 22 vs. entry 10). Then, the effect of the amount of additive and reaction time was examined; unfortunately, no better results were obtained (Table 1, entries 23–25). Finally, the optimal reaction conditions were identified as follows: The mixture of *1a* (0.5 mmol), *2a* (0.2 mmol), Na_2_CO_3_ (0.2 mmol), and 15-crown-5 (0.2 mmol) was stirred in toluene at 140°C under N_2_ atmosphere for 24 h.

**TABLE 1:** Optimization of reaction conditions^a^.

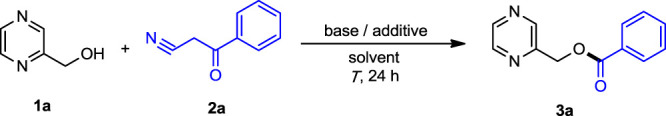

Entry	Base	Additive	Solvent	T (°C)	Yields of 3a (%)^b^
1	*t*-BuOK	-	Toluene	140	10
2	-	-	Toluene	140	0
3	KOH	-	Toluene	140	11
4	K_2_CO_3_	-	Toluene	140	12
5	KHMDS	-	Toluene	140	10
6	NaOH	-	Toluene	140	18
7	*t*-BuONa	-	Toluene	140	16
8	NaOAc	-	Toluene	140	15
9	Na_2_CO_3_	-	Toluene	140	25
10	Na_2_CO_3_	15-Crown-5	Toluene	140	61
11	Na_2_CO_3_	PPh_3_	Toluene	140	32
12	Na_2_CO_3_	1,10-Phen	Toluene	140	29
13	Na_2_CO_3_	TMEDA	Toluene	140	30
14	Na_2_CO_3_	15-Crown-5	1,4-Dioxane	140	40
15	Na_2_CO_3_	15-Crown-5	DMF	140	Trace
16	Na_2_CO_3_	15-Crown-5	DMSO	140	42
17	Na_2_CO_3_	15-Crown-5	DCE	140	0
18	Na_2_CO_3_	15-Crown-5	THF	140	41
19	Na_2_CO_3_	15-Crown-5	Toluene	120	50
20	Na_2_CO_3_	15-Crown-5	Toluene	150	62
21^c^	Na_2_CO_3_	15-Crown-5	Toluene	140	56
**22** **^d^**	**Na**_**2**_ **CO**_**3**_	**15-Crown-5**	**Toluene**	**140**	**85**
23^d^ ^,^ ^e^	Na_2_CO_3_	15-Crown-5	Toluene	140	83
24^d^ ^,^ ^f^	Na_2_CO_3_	15-Crown-5	Toluene	140	69
25^d^ ^,^ ^g^	Na_2_CO_3_	15-Crown-5	Toluene	140	81

a Note. 2-Pyrazinylmethanol 1a (0.2 mmol), Benzoylacetonitrile 2a (0.2 mmol), base (1.0 eq), additive (1.0 eq), and toluene (1 ml) for 24 h, under N_2_ atmosphere.

b Isolated yields.

c 1a/2a ratio = 0.2/0.5.

d 1a/2a ratio = 0.5/0.2.

e Additive (1.5 eq) was used.

f Additive (0.5 eq) was used.

g 48 h.

**SCHEME 2:**
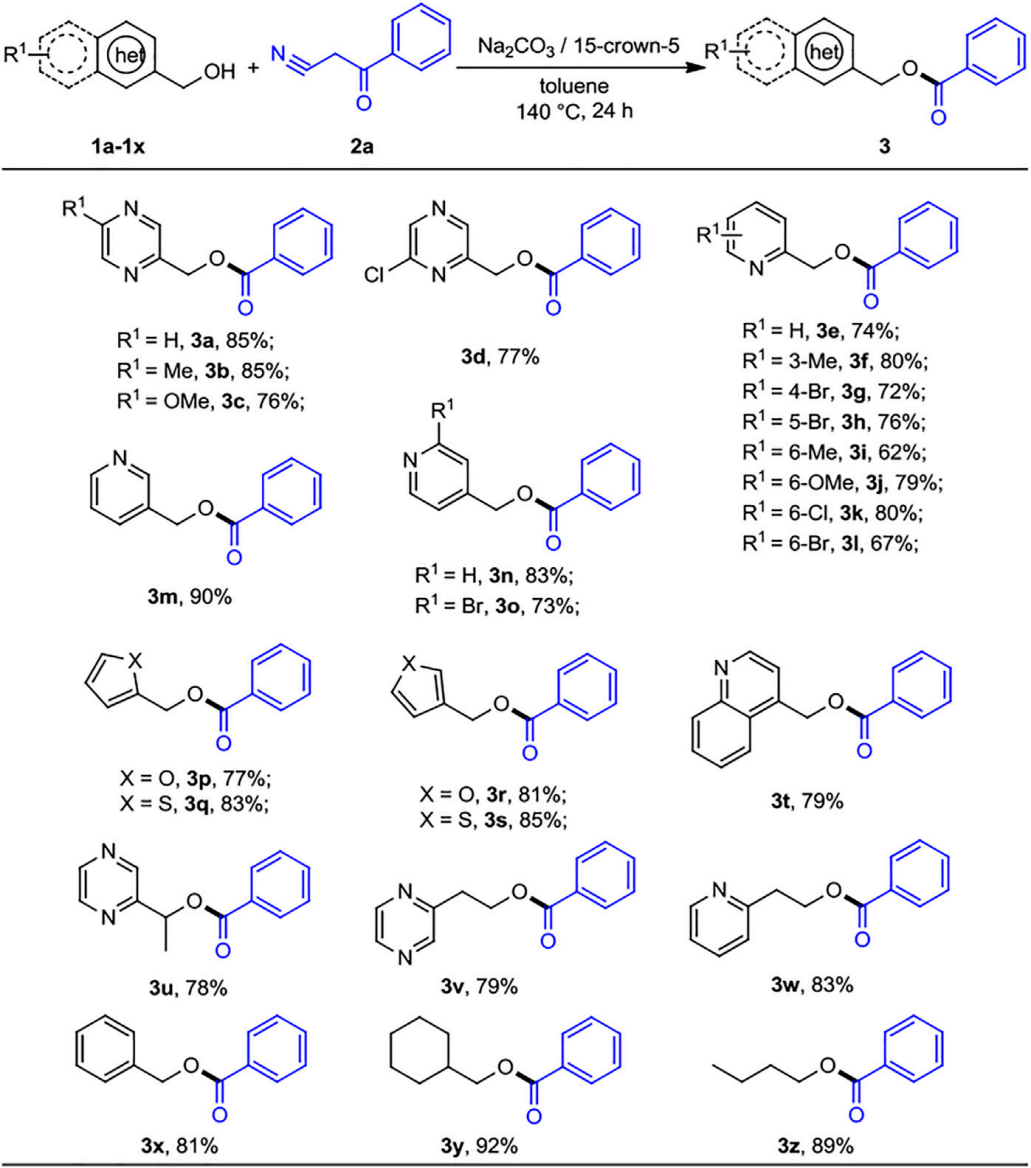
Substrate scope of heteroaryl methanols for the acylation reactions. ^*a,b*^Reaction conditions: ^*a*^heteroaryl methanols 1 (0.5 mmol), benzoylacetonitrile 2 (0.2 mmol), Na_2_CO_3_ (0.2 mmol), 15-crown-5 (0.2 mmol), and toluene (1 ml) at 140°C for 24 h, under N_2_ atmosphere. ^*b*^Isolated yields.

With the optimized reaction conditions in hand, we next sought to generalize the protocol on a range of diverse substrates. As shown in Scheme 2, various commercially available *N*-heteroaryl methanols and benzoylacetonitrile were exposed to the standard reaction conditions, and the desired heteroarenemethyl benzoates were successfully afforded with good yields. Initially, pyrazinylmethanol was substituted by an electron-donating group such as Me− or MeO−, and we obtained the desired products with moderate to good yields (*3b*, 85%) and (*3c*, 76%), respectively, while, the electron-withdrawing substituent Cl− also gave the corresponding ester *3d* at a moderate yield of 77%. 2-pyridinylmethanols with 3-methyl, 6-methyl, 6-methoxy, 4-Br, 5-Br, 6-Cl, and 6-Br groups were converted to the corresponding products with lower yields (*3e-3l*, 62%–80% yields). It was worth mentioning that the yields were slightly decreased by the presence of different substituents on the 2-pyridinylmethanols. However, 3- and 4-pyridinylmethanol exhibited good reactivity obtaining the esterification products *3m* and *3n* in 90% and 83% yield, respectively. 4-Pyridinylmethanol bearing electron-withdrawing substituent (2-Br) revealed the lower yield of desired product (*3o*, 73%). The reactions of benzoylacetonitrile with furanmethanols, thiophenemethanols, and 4-quinolylmethanol also smoothly afforded the corresponding products *3p*–*3t* in yields of 77%–85%. As we expected, both α-methyl-2-pyrazinemethanol, 2-pyrazinylethyl alcohol, and 2-pyridinylethyl alcohol worked well to give the corresponding products *3u*–*3w* in 78%–83% yield. Interestingly, simple alcohols such as benzyl and aliphatic (*1v*–*1y*) could be used as substrates under the present conditions, and the desired products (*3x*–*3z*) were obtained at the yields of 81%, 92%, and 88%, respectively. The results indicated that the simple unhindered aliphatic alcohols show the excellent nucleophilic activity.

**SCHEME 3:**
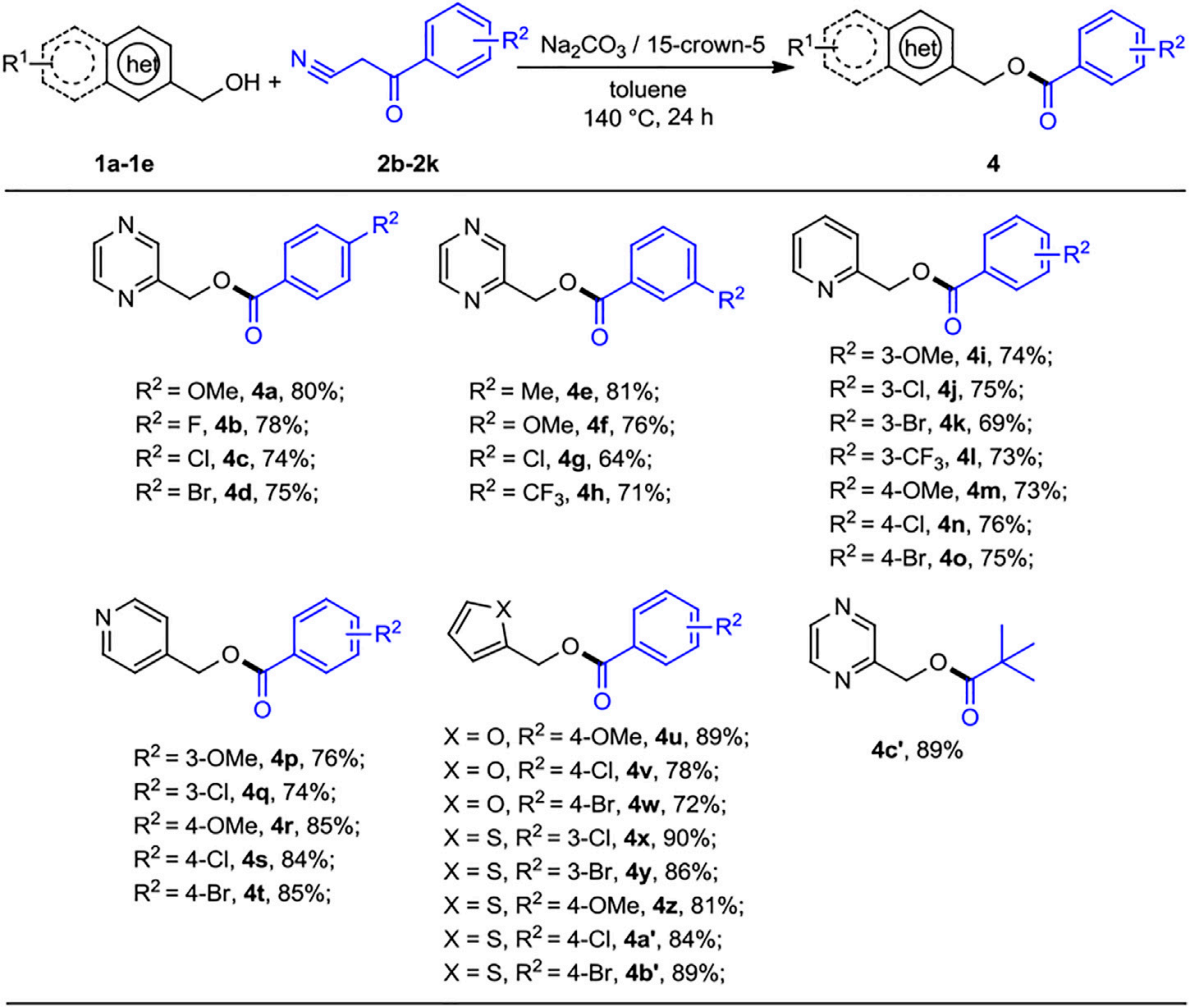
Substrate scope of benzoylacetonitriles for the acylation reactions. ^a,b^Reaction conditions: ^*a*^heteroaryl methanols 1 (0.5 mmol), benzoylacetonitrile derivatives 2 (0.2 mmol), Na_2_CO_3_ (0.2 mmol), 15-crown-5 (0.2 mmol), and toluene (1 ml) at 140°C for 24 h, under N_2_ atmosphere. ^*b*^Isolated yields.

Next, the scope of the reaction was evaluated on various benzoylacetonitrile derivatives under the optimized reaction conditions, and the results are summarized in Scheme 3. Benzoylacetonitrile containing electron-donating groups methoxy (*2b*, *2g*) and methyl (*2f*) were successfully transformed to the corresponding esters to give the products (*4a*, *4e*, and *4f*) at good yields of 76%–81%. Slight lower yields were obtained when electron-withdrawing groups 4-F, 4-Cl, 4-Br, 3-Cl, and 3-CF_3_ substituted benzoylacetonitriles with 2-pyrazinylmethanol were subjected to this transformation (*4b*–*4d*, *4g*–*4h*, 64%–78% yields). Interestingly, 2- and 4-pyridinylmethanol bearing the OMe group and halogen atom (Cl, Br, and CF_3_) at the *para* or *meta* position are well tolerated, as the products of pyridin-2-ylmethyl and -4-ylmethyl substituted benzoates, *4i*–*4t* were obtained in 73%–85% yields. The reactions of benzoylacetonitriles substituted at the *para* or *meta* position by various groups (OMe, Cl, and Br) with 2-furanmethanol and 2-thiophenemethanol underwent a smooth reaction to provide the products *4u*–*4b′* in good to excellent yields. Moreover, pyvaolylacetonitrile displayed the good reactivity, as *4c′* was isolated in 89% yield.

**SCHEME 4:**
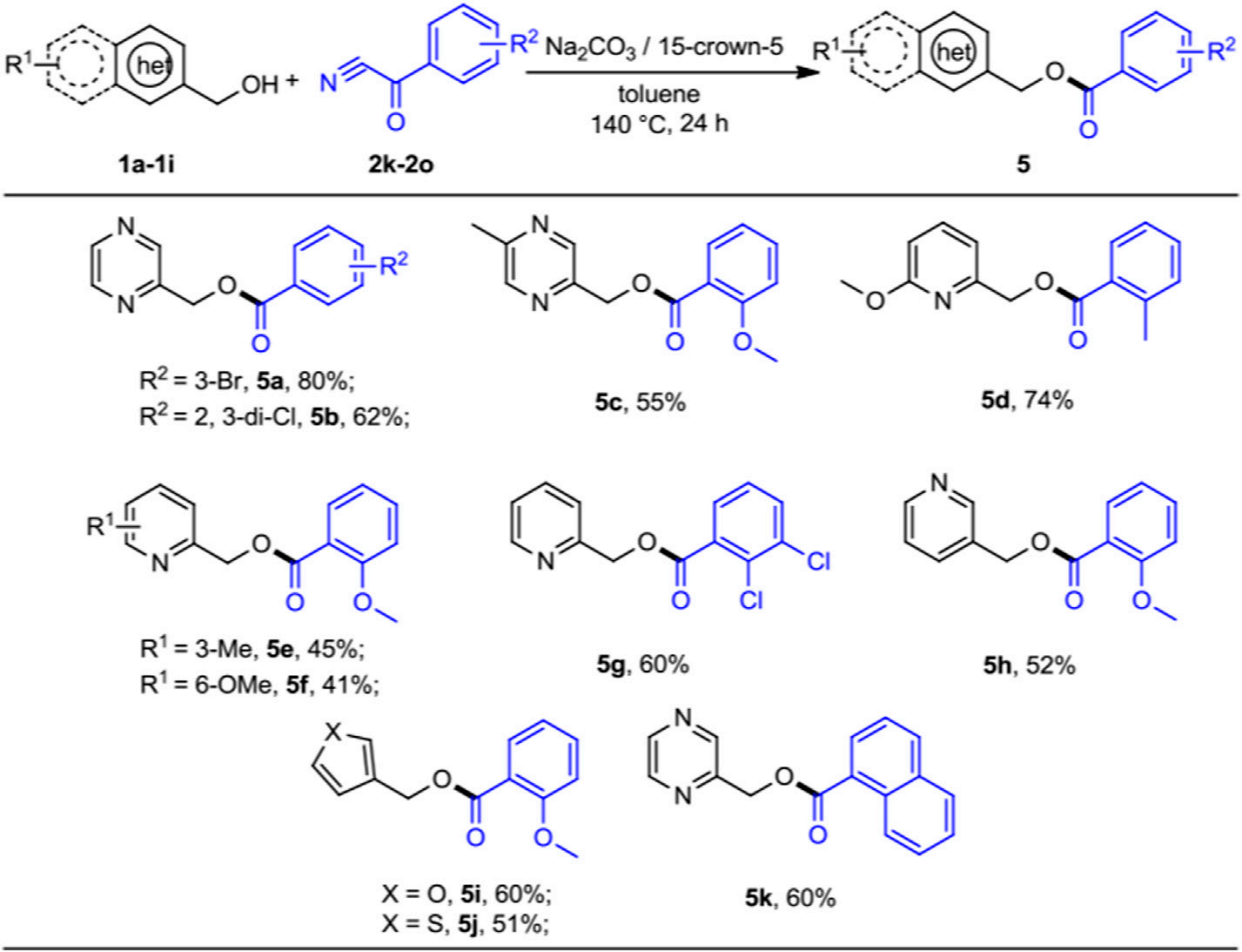
Substrate scope of benzoyl cyanides for the acylation reactions. ^*a,b*^Reaction conditions: ^*a*^heteroaryl methanols 1 (0.5 mmol), benzoyl cyanides 2 (0.2 mmol), Na_2_CO_3_ (0.2 mmol), 15-crown-5 (0.2 mmol), and toluene (1 ml) at 140°C for 24 h, under N_2_ atmosphere. ^*b*^Isolated yields.

Finally, we also briefly set out to evaluate the scope of aryl cyanides (Scheme 4). As expected, these reactions proceeded smoothly when 2-pyrazinylmethanol and pyridinylmethanols with benzoyl cyanides served as the substrates, leading the corresponding products *5a*–5h in moderate to good yields (41%–80%). 3-furanmethanol and 3-thiophenemethanol could undergo the expected acylation with 2-methoxy-α-oxo-benzeneacetonitrile (*2m*) in moderate yields (*5i*–*5j*, 51%–60%). We were pleased to observe that α-oxo-1-naphthaleneacetonitrile was readily reacted with *1a* in moderate yield (*5k*, 60%). It was satisfying to discover that the protocol was amenable for most benzoyl cyanides substrates, which is different from the work of Subaramannian et al. involving the single-electron transfer (SET) in the catalytic transformation (Subaramanian et al., 2020). This was probably because the nature of reaction mechanism of these two methods is distinct.

**SCHEME 5:**
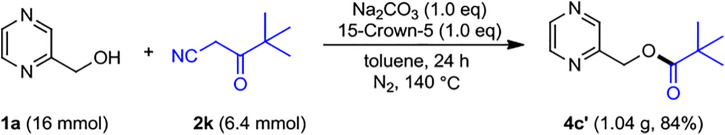
Gram-scale reaction of 1a and 2k.

**SCHEME 6:**
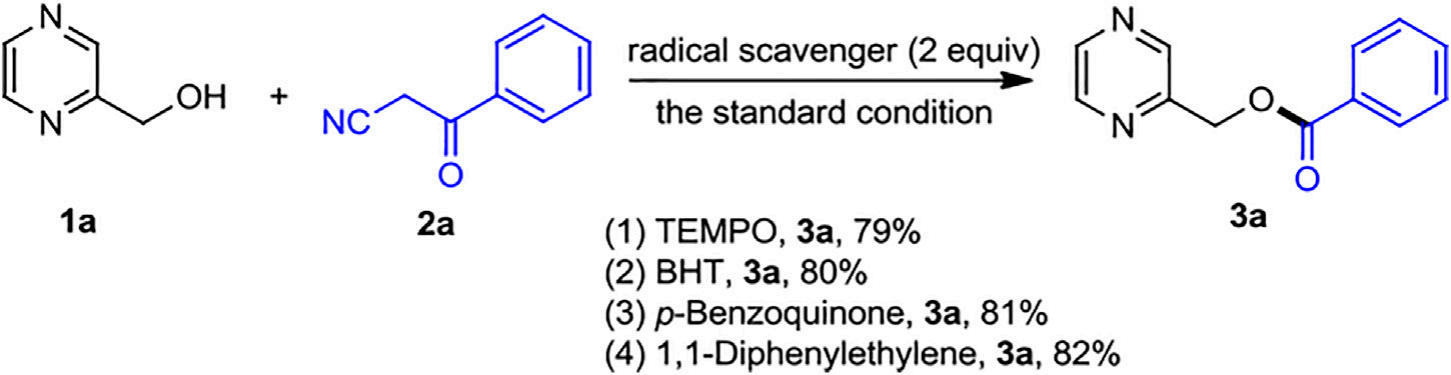
Mechanistic experiments.

The gram-scale reaction of *1a* (16 mmol, 1.761 g) and *2k* (6.4 mmol, 0.801 g) was carried out to demonstrate the practicability of this protocol, which could give the desired product *4c′* in 84% yield (Scheme 5). As shown in Scheme 6, radical trapping experiments were performed through the addition of TEMPO, BHT, *p*-benzoquinone, and 1,1-diphenylethylene to the reaction system in the standard conditions, and the formation of *3a* was not suppressed. These results inferred that this transformation did not occur *via* a radical mechanism.

**SCHEME 7:**
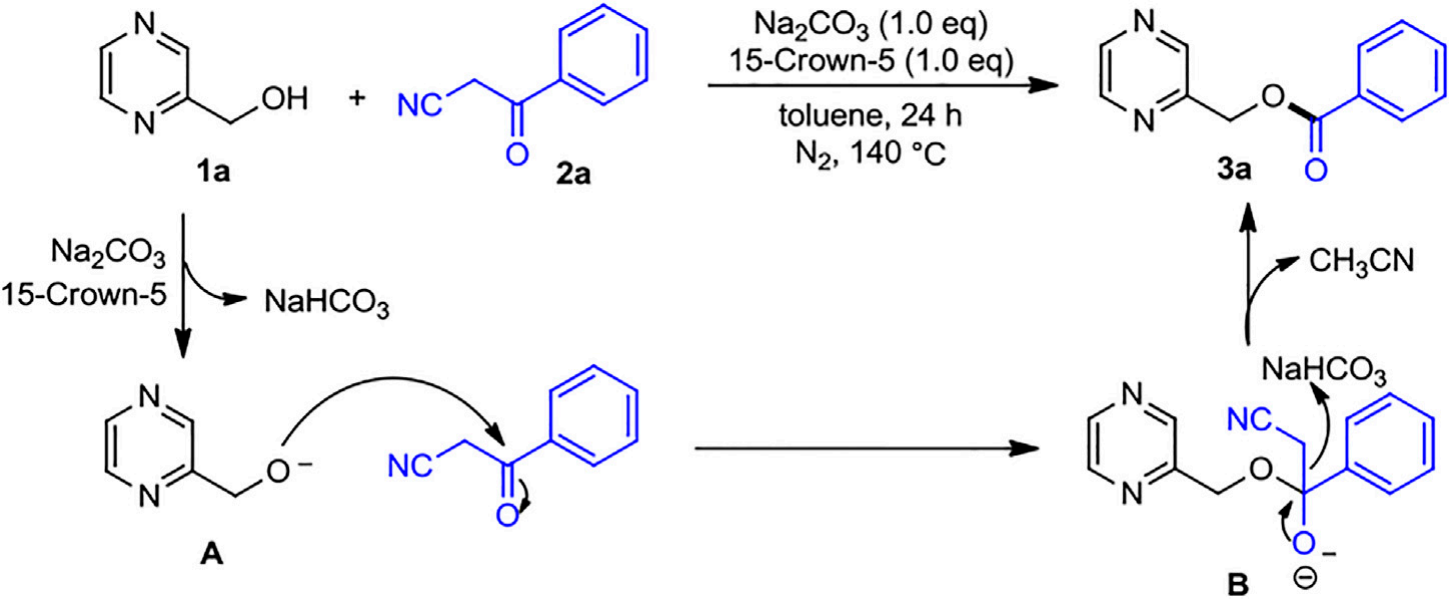
Plausible reaction mechanism.

A tentative mechanism was proposed and is shown in Scheme 7 on the basis of the results presented above and previous reports (Xie et al., 2014; Kong et al., 2016). The reaction initiated with Na_2_CO_3_ associated with 15-crown-5 induced deprotonation of 2-pyrazinylmethanol *1a* to form 2-pyrazinyl alkoxy anion *A*. Then anion *A* undergoes a nucleophilic attack to the carbonyl group of benzoylacetonitrile *2a* and affords intermediate *B*. Moreover, by abstracting a proton from the *in situ* formed NaHCO_3_, the thermodynamic favorable C–C bond cleavage of *B* would provide the desired product *3a*, in which the side product CH_3_CN is released.

## Conclusion

In summary, Na_2_CO_3_/15-crown-5 couple-mediated direct acylation of *N*-heteroaryl methanols with acyl cyanides *via* C–C bond cleavage is reported. This new synthetic protocol proceeds under metal-free conditions and offers broad substrate scope in high efficiency. A variety of *N*-heteroaryl esters including pyrazines, pyridines, quinolines, furans, and thiophenes, which are key molecules in pharmaceuticals, natural products, dyes, or flavors, have thus been efficiently synthesized with good yields.

## Data Availability

The original contributions presented in the study are included in the article/Supplementary Material, Further inquiries can be directed to the corresponding author.
