# Supplementary material for: Synthesis of N-Heteroarenemethyl Esters via C–C Bond Cleavage of Acyl Cyanides Under Transition Metal-Free Conditions
Source: Front Chem. 2022 Jan 27;9:822625. doi: 10.3389/fchem.2021.822625 (PMC8828493; doi:10.3389/fchem.2021.822625)
Supplement: Supplementary file 1 [file DataSheet1.PDF]

Supplementary Material

Synthesis of *N*-Heteroarenemethyl Esters *via* C-C Bond Cleavage of Acyl Cyanides under Transition-Metal Free Conditions

Miao Lai[#], Fangyao Su[#], Jingyi Hu, Mengzhuo Wang, Mingqin Zhao*, Ganlin Zhang

Flavors and Fragrance Engineering & Technology Research Center of Henan Province, College of Tobacco Science, Henan Agricultural University, Zhengzhou 450002, P.R. China

zhaomingqin@126.com

Contents

1. **General Information**……………………………………………………… **S-2**
2. **Experimental section**……………………………………………………… **S-2**
3. **Mechanistic investigation**…………………………………………………… **S-2**
4. **Characterization data**……………………………………………………… **S-3**
5. **References**………………………………………………………………… **S-15**
6. **Copy of ^{1}H and ^{13}C NMR Spectra**………………………………………… **S-17**

Supplementary Material

1 General information

All the reagents were obtained commercially and used without further purification. Silica gel was purchased from Qing Dao Hai Yang Chemical Industry Co. Analytical thin layer chromatography (TLC) was performed on precoated silica gel F_{254} plates. Compounds were visualized by irradiation with UV light (254 nm).

Analytical information: ^{1}H NMR and ^{13}C NMR spectra data were recorded by a BRUKER AVANCE III 400 MHz spectrometer (^{1}H 400 MHz, ^{13}C 100 MHz), using $CDCl_3$ as the solvent with tetramethylsilane (TMS) as the internal standard at room temperature. ^{1}H NMR spectral data are given as chemical shifts in ppm: followed by multiplicity (s-singlet; d-doublet; t-triplet; q-quartet; m-multiplet), number of protons and coupling constants. ^{13}C NMR chemical shifts are expressed in ppm. HRMS data were obtained using AB SCIEX Triple TOF 5600+ high resolution mass spectrometer (USA). The products listed below were determined by ^{1}H and ^{13}C NMR spectra. Infrared spectra were recorded with a Thermo Scientific Nicolet 6700 FT-IR Spectrometer. Melting points were determined using melting point X-4 (Gongyi Kerui) apparatus.

2 Experimental Section

General procedure: Reaction of N-heteroaryl methanols with acyl cyanides

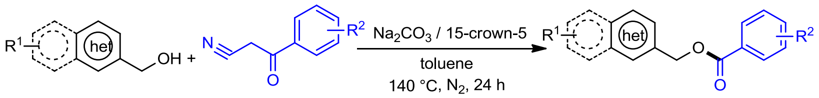

1 **2** **3-5**

In an oven dried screw cap reaction tube (15 mL), the *N*-heteroaryl methanols **1** (0.5 mmol), acyl cyanides **2** (0.2mmol), Na_2CO_3 (0.2 mmol), 15-crown-5 (0.2mmol) and toluene (1 mL) were added in a gentle stream of nitrogen. Then the reaction mixture was stirred with a magnetic stirring bar at 140 °C (oil-bath temperature) for 24 h. After completion of the reaction, the crude mixture was worked up with water and extracted using dichloromethane, followed by the solvent was removed under reduced pressure and finally the residue was purified by silica gel column chromatography (230-400 mesh size) using *n*-pentane and ethyl acetate as an eluent to give the corresponding esters **3, 4** and **5** in moderate to good yields.

3 Mechanistic investigation

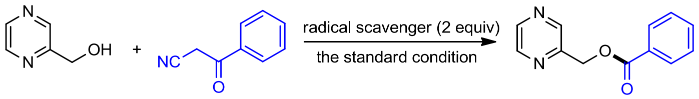

1a **2a** **3a**

(1) TEMPO, **3a**, 79%
(2) BHT, **3a**, 80%
(3) *p*-Benzoquinone, **3a**, 81%
(4) 1,1-Diphenylethylene, **3a**, 82%

In an oven dried screw cap reaction tube (15 mL), 2-pyrazinylmethanol **1a** (0.5 mmol), benzoylacetonitrile **2a** (0.2 mmol), Na_2CO_3 (0.2 mmol), 15-crown-5 (0.2 mmol), radical quencher (0.2 mmol) and toluene (1 mL) were added in a gentle stream of nitrogen. Then, the reaction mixture was stirred with a magnetic stirring bar at 140 °C (oil-bath temperature) for 24 h. After completion of the reaction, the crude mixture was worked up with water and extracted using dichloromethane, followed by the solvent was removed under reduced pressure and finally the residue was purified by

silica gel column chromatography (230-400 mesh size) using *n*-pentane and ethyl acetate as an eluent to obtain the desired product (**3a**).

4 Characterization Data

pyrazin-2-ylmethyl benzoate (3a)[1]

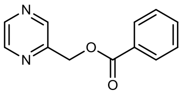

Isolated yield = 88%; white solid; ^{1}H NMR (400 MHz, $CDCl_3$) ppm: 8.78 (d, *J* = 1.1 Hz, 1H), 8.63 – 8.58 (m, 1H), 8.56 (d, *J* = 2.5 Hz, 1H), 8.12 (dt, *J* = 8.5, 1.5 Hz, 2H), 7.60 (ddd, *J* = 8.7, 2.6, 1.3 Hz, 1H), 7.51 – 7.43 (m, 2H), 5.53 (s, 2H); ^{13}C NMR (100 MHz, $CDCl_3$) ppm: 166.09, 151.61, 144.18, 143.76, 133.45, 129.84, 129.42, 128.54, 65.16; CAS Number: 1025351-04-5.

(5-methylpyrazin-2-yl) methyl benzoate (3b)

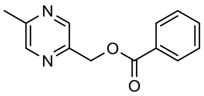

Isolated yield = 85%; light yellow liquid; ^{1}H NMR (400 MHz, $CDCl_3$) ppm: 8.65 (d, *J* = 1.0 Hz, 1H), 8.47 (s, 1H), 8.12 – 8.06 (m, 2H), 7.62 – 7.54 (m, 1H), 7.50 – 7.41 (m, 2H), 5.48 (s, 2H), 2.59 (s, 3H); ^{13}C NMR (100 MHz, $CDCl_3$) ppm: 166.17, 153.46, 148.13, 143.97, 142.86, 133.35, 129.82, 129.55, 128.49, 65.14, 21.39; IR (KBr) ν_{max} 3065, 2927, 1724, 1488, 1451, 1274, 1112, 711, and 671 cm^{-1}; HRMS (ESI) calcd. for $C_{13}H_{12}N_2O_2$: [M+H]$^+$: 229.0977, found: 229.0976.

(5-methoxypyrazin-2-yl) methyl benzoate (3c)

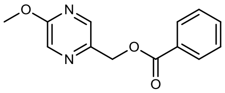

Isolated yield = 76%; light yellow liquid; ^{1}H NMR (400 MHz, $CDCl_3$) ppm: 8.28 (d, *J* = 1.2 Hz, 1H), 8.24 (d, *J* = 1.3 Hz, 1H), 8.09 (d, *J* = 1.2 Hz, 1H), 8.07 (t, *J* = 1.7 Hz, 1H), 7.60 – 7.52 (m, 1H), 7.47 – 7.40 (m, 2H), 5.43 (s, 2H), 3.98 (s, 3H); ^{13}C NMR (100 MHz, $CDCl_3$) ppm: 166.26, 160.13, 142.48, 140.20, 135.29, 133.23, 129.78, 129.73, 128.42, 64.87, 53.82; IR (KBr) ν_{max} 3067, 2945, 2851, 1723, 1541, 1483, 1273, 1029, and 711 cm^{-1}; HRMS (ESI) calcd. for $C_{13}H_{12}N_2O_3$: [M+H]$^+$: 245.0926, found: 245.0924.

(6-chloropyrazin-2-yl) methyl benzoate (3d)

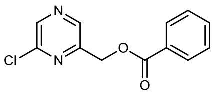

Isolated yield = 77%; light yellow solid; m. p. 74 – 75 °C; ^{1}H NMR (400 MHz, $CDCl_3$) ppm: 8.67 (s, 1H), 8.58 (s, 1H), 8.14 – 8.08 (m, 2H), 7.66 – 7.58 (m, 1H), 7.52 – 7.45 (m, 2H), 5.49 (s, 2H); ^{13}C NMR (100 MHz, $CDCl_3$) ppm: 165.93, 151.45, 148.80, 144.06, 141.07, 133.60, 129.87, 129.18, 128.59, 64.40; IR (KBr) ν_{max} 3062, 2922, 2851, 1709, 1564, 1524, 1293, 1004, and 710 cm^{-1}; HRMS (ESI) calcd. for $C_{12}H_9ClN_2O_2$: [M+H]$^+$: 249.0431, found: 249.0430.

pyridin-2-ylmethyl benzoate (3e)[1]

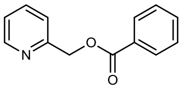

Isolated yield = 74%; deep yellow liquid; ^{1}H NMR (400 MHz, $CDCl_3$) ppm: 8.62 (dd, *J* = 4.8, 0.6 Hz, 1H), 8.17 – 8.10 (m, 2H), 7.72 (dt, *J* = 7.7, 1.8 Hz, 1H), 7.63 – 7.55 (m, 1H), 7.47 (dd, *J* = 10.7, 4.5 Hz, 3H), 7.27 – 7.23 (m, 1H), 5.50 (s, 2H), 5.50 (s, 2H); ^{13}C NMR (100 MHz, $CDCl_3$) ppm: 166.25, 156.02, 149.49, 136.84, 133.22, 129.83, 128.46, 122.89, 121.71, 67.22; CAS Number: 66310-15-4.

(3-methylpyridin-2-yl) methyl benzoate (3f)

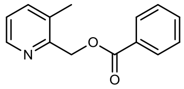

Isolated yield = 80%; deep yellow liquid; ^{1}H NMR (400 MHz, $CDCl_3$) ppm: 8.46 (d, J = 4.0 Hz, 1H), 8.13 – 8.01 (m, 2H), 7.59 – 7.49 (m, 2H), 7.43 (t, J = 7.7 Hz, 2H), 7.21 (dd, J = 7.6, 4.8 Hz, 1H), 5.49 (s, 2H), 2.43 (s, 3H); ^{13}C NMR (100 MHz, $CDCl_3$) ppm: 166.35, 153.41, 146.94, 138.34, 133.07, 132.73, 129.90, 129.80, 128.37, 123.52, 66.51, 18.17; IR (KBr) ν_{max} 3445, 2962, 2925, 2853, 1720, 1645, 1274, 1109, 712 cm^{-1}; HRMS (ESI) calcd. for $C_{14}H_{13}NO_2$: $[M+H]^+$: 228.1025, found: 228.1025.

(4-bromopyridin-2-yl) methyl benzoate (3g)

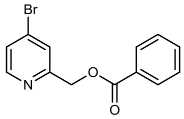

Isolated yield = 72%; light yellow liquid; ^{1}H NMR (400 MHz, $CDCl_3$) ppm: 8.43 (d, J = 5.3 Hz, 1H), 8.13 (dd, J = 8.4, 1.3 Hz, 2H), 7.66 – 7.58 (m, 2H), 7.48 (t, J = 6.9 Hz, 2H), 7.43 (dd, J = 5.3, 1.8 Hz, 1H), 5.47 (s, 2H); ^{13}C NMR (100 MHz, $CDCl_3$) ppm: 166.09, 157.60, 150.19, 133.68, 133.41, 129.85, 129.55, 128.54, 126.30, 124.95, 66.44; IR (KBr) ν_{max} 2923, 2851, 1726, 1601, 1573, 1269, 1114, 1070, and 710 cm^{-1}; HRMS (ESI) calcd. for $C_{13}H_{10}BrNO_2$: $[M+H]^+$: 291.9973, found: 291.9970.

(5-bromopyridin-2-yl) methyl benzoate (3h)

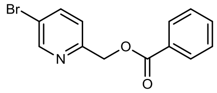

Isolated yield = 76%; deep yellow solid; m. p. 49 – 50 °C; ^{1}H NMR (400 MHz, $CDCl_3$) ppm: 8.67 (d, J = 2.1 Hz, 1H), 8.14 – 8.07 (m, 2H), 7.84 (dd, J = 8.3, 2.3 Hz, 1H), 7.59 (t, J = 7.4 Hz, 1H), 7.46 (t, J = 7.7 Hz, 2H), 7.36 (d, J = 8.3 Hz, 1H), 5.44 (s, 2H); ^{13}C NMR (100 MHz, $CDCl_3$) ppm: 166.13, 154.59, 150.58, 139.42, 133.36, 129.81, 129.62, 128.51, 123.06, 119.91, 66.55; IR (KBr) ν_{max} 3065, 2921, 2850, 1705, 1576, 1449, 1277, 1115, and 708 cm^{-1}; HRMS (ESI) calcd. for $C_{13}H_{10}BrNO_2$: $[M+H]^+$: 291.9973, found: 291.9973.

(6-methylpyridin-2-yl) methyl benzoate (3i)[1]

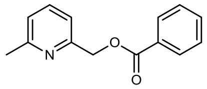

Isolated yield = 62%; deep yellow liquid; ^{1}H NMR (400 MHz, $CDCl_3$) ppm: 7.57 (t, J = 7.7 Hz, 1H), 7.06 (d, J = 2.5 Hz, 1H), 7.04 (d, J = 2.6 Hz, 1H), 4.72 (s, 2H), 2.55 (s, 3H); ^{13}C NMR (100 MHz, $CDCl_3$) ppm: 158.13, 157.39, 136.99, 121.82, 117.46, 63.95, 24.18; CAS Number: 2161397-20-0.

(6-methoxypyridin-2-yl) methyl benzoate (3j)

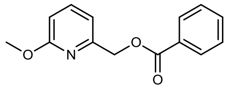

Isolated yield = 79%; white solid; m. p. 47 – 48 °C; ^{1}H NMR (400 MHz, $CDCl_3$) ppm: 8.13 (dd, J = 8.4, 1.3 Hz, 2H), 7.64 – 7.54 (m, 2H), 7.51 – 7.42 (m, 2H), 6.99 (d, J = 7.8 Hz, 1H), 6.67 (d, J = 8.0 Hz, 1H), 5.39 (s, 2H), 3.92 (s, 3H); ^{13}C NMR (100 MHz, $CDCl_3$) ppm: 166.28, 163.78, 153.61, 139.06, 133.15, 130.05, 129.78, 128.45, 113.76, 109.87, 66.83, 53.40; IR (KBr) ν_{max} 2926, 2851, 1721, 1596, 1582, 1327, 1566, 1123, and 709 cm^{-1}; HRMS (ESI) calcd. for $C_{14}H_{13}NO_3$: $[M+H]^+$: 244.0974, found: 244.0971.

(6-chloropyridin-2-yl) methyl benzoate (3k)

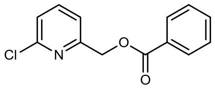

Isolated yield = 80%; light yellow liquid; ^{1}H NMR (400 MHz, $CDCl_3$) ppm: 8.12 (dt, J = 8.5, 1.5 Hz, 2H), 7.68 (t, J = 7.8 Hz, 1H), 7.63 – 7.56 (m, 1H), 7.51 – 7.44 (m, 2H), 7.38 (dd, J = 7.6, 0.6 Hz, 1H), 7.29 (d, J = 7.9 Hz, 1H), 5.45 (s, 2H); ^{13}C NMR (100 MHz, $CDCl_3$) ppm: 166.05, 156.97, 151.05, 139.46, 133.38, 129.82, 129.59, 128.52, 123.52, 119.98, 66.37; IR (KBr) ν_{max} 3064, 2924, 2850, 1723, 1587, 1278, 1112, 987, and 712 cm^{-1}; HRMS (ESI) calcd. for $C_{13}H_{10}ClNO_2$: [M+H]$^+$: 248.0478, found: 248.0480.

(6-bromopyridin-2-yl) methyl benzoate (3l)

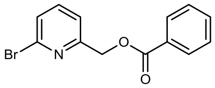

Isolated yield = 67%; yellow liquid; ^{1}H NMR (400 MHz, $CDCl_3$) ppm: 8.38 (d, J = 5.1 Hz, 1H), 8.12 – 8.06 (m, 2H), 7.66 – 7.59 (m, 1H), 7.55 (s, 1H), 7.49 (t, J = 7.7 Hz, 2H), 7.30 (d, J = 5.0 Hz, 1H), 5.35 (s, 2H); ^{13}C NMR (100 MHz, $CDCl_3$) ppm: 165.92, 150.37, 148.15, 142.64, 133.62, 129.79, 129.21, 128.63, 126.15, 120.97, 63.84; IR (KBr) ν_{max} 3061, 2924, 2851, 1724, 1593, 1549, 1269, 1111, and 711 cm^{-1}; HRMS (ESI) calcd. for $C_{13}H_{10}BrNO_2$: [M+H]$^+$: 291.9973, found: 291.9973.

pyridin-3-ylmethyl benzoate (3m)[2]

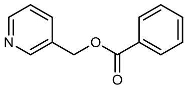

Isolated yield = 90%; white solid; ^{1}H NMR (400 MHz, $CDCl_3$) ppm: 8.73 (d, J = 1.8 Hz, 1H), 8.60 (dd, J = 4.8, 1.5 Hz, 1H), 8.10 – 8.03 (m, 2H), 7.82 – 7.76 (m, 1H), 7.61 – 7.54 (m, 1H), 7.48 – 7.41 (m, 2H), 7.33 (ddd, J = 7.8, 4.9, 0.5 Hz, 1H), 5.39 (s, 2H); ^{13}C NMR (100 MHz, CDCl3) ppm: 166.27, 149.67, 149.66, 136.03, 133.29, 131.70, 129.71, 129.70, 128.48, 123.53, 64.15; CAS Number: 58550-50-8.

pyridin-4-ylmethyl benzoate (3n)[2]

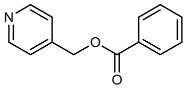

Isolated yield = 83%; white solid; ^{1}H NMR (400 MHz, $CDCl_3$) ppm: 8.63 (dd, J = 4.6, 1.4 Hz, 2H), 8.18 – 8.05 (m, 2H), 7.64 – 7.56 (m, 1H), 7.51 – 7.44 (m, 2H), 7.34 (d, J = 6.0 Hz, 2H), 5.38 (s, 2H); ^{13}C NMR (100 MHz, $CDCl_3$) ppm: 166.09, 150.09, 145.06, 133.44, 129.73, 128.56, 121.86, 64.65; CAS Number: 46721-39-1.

(2-bromopyridin-4-yl) methyl benzoate (3o)

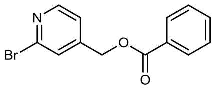

Isolated yield = 73%; light yellow liquid; ^{1}H NMR (400 MHz, $CDCl_3$) ppm: 8.38 (d, J = 5.1 Hz, 1H), 8.13 – 8.07 (m, 2H), 7.65 – 7.59 (m, 1H), 7.55 (d, J = 0.6 Hz, 1H), 7.49 (t, J = 7.7 Hz, 2H), 7.30 (d, J = 5.1 Hz, 1H), 5.35 (s, 2H); ^{13}C NMR (100 MHz, $CDCl_3$) ppm: 165.93, 150.38, 148.14, 142.65, 133.62, 129.80, 129.21, 128.64, 126.15, 120.97, 63.84; IR (KBr) ν_{max} 3061, 2922, 2850, 1724, 1593, 1549, 1269, 1111, and 711 cm^{-1}; HRMS (ESI) calcd. for $C_{13}H_{10}BrNO_2$: [M+H]$^+$: 291.9973, found: 291.9972.

furan-2-ylmethyl benzoate (3p)[2]

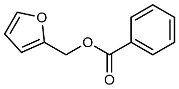

Isolated yield = 83%; colorless liquid; ^{1}H NMR (400 MHz, $CDCl_3$) ppm: 8.09 – 8.02 (m, 2H), 7.61 – 7.50 (m, 1H), 7.48 – 7.37 (m, 3H), 6.49 (d, J = 3.2 Hz, 1H), 6.38 (dd, J = 3.2, 1.9 Hz, 1H), 5.31 (s,

2H); ^{13}C NMR (100 MHz, $CDCl_3$) ppm: 166.27, 149.56, 143.31, 133.10, 129.91, 129.77, 128.36, 110.81, 110.61, 58.52; CAS Number: 34171-46-5.

thiophen-2-ylmethyl benzoate (3q)[2]

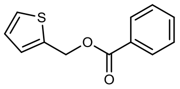

Isolated yield = 77%; light yellow liquid; ^{1}H NMR (400 MHz, $CDCl_3$) ppm: 8.06 (dt, J = 7.8, 0.48 Hz, 2H), 7.57 – 7.53 (m, 1H),7.45-7.40 (m, 2H),7.37 (dd, J = 5.08, 1.16 Hz, 1H), 7.18-7.17 (m, 1H), 7.01 (dd, J = 5.08, 1.56 Hz, 1H), 5.51 (s, 2H); ^{13}C NMR (100 MHz, $CDCl_3$) ppm: 166.29, 138.02, 133.13, 129.93, 129.76, 128.39, 128.21, 126.87, 61.06; CAS Number: 85455-66-9.

furan-3-ylmethyl benzoate (3r)[3]

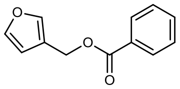

Isolated yield = 81%; colorless liquid; ^{1}H NMR (400 MHz, $CDCl_3$) ppm: 8.40 (d, *J* = 4.9 Hz, 1H), 8.13 – 8.07 (m, 2H), 7.66 – 7.58 (m, 1H), 7.53 – 7.46 (m, 2H), 7.39 (dd, *J* = 1.3, 0.7 Hz, 1H), 7.29 – 7.24 (m, 1H), 5.37 (s, 2H); ^{13}C NMR (100 MHz, $CDCl_3$) ppm: 165.94, 152.04, 149.97, 148.45, 133.62, 129.79, 129.22, 128.63, 122.35, 120.57, 63.96; CAS Number: 98096-44-7.

thiophen-3-ylmethyl benzoate (3s)[3]

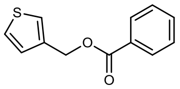

Isolated yield = 86%; yellow liquid; ^{1}H NMR (400 MHz, $CDCl_3$) ppm: 8.06 (dd, *J* = 8.3, 1.3 Hz, 2H), 7.59 – 7.52 (m, 1H), 7.47 – 7.40 (m, 2H), 7.37 (d, *J* = 2.2 Hz, 1H), 7.33 (dd, *J* = 4.9, 3.0 Hz, 1H), 7.17 (dd, *J* = 5.0, 1.1 Hz, 1H), 5.37 (s, 2H); ^{13}C NMR (100 MHz, $CDCl_3$) ppm: 166.42, 136.89, 133.07, 130.11, 129.71, 128.39, 127.63, 126.28, 124.32, 61.85; CAS Number: 182814-05-7.

quinolin-4-ylmethyl benzoate (3t)[4]

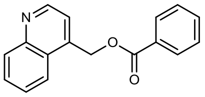

Isolated yield = 79%; white solid; ^{1}H NMR (400 MHz, $CDCl_3$) ppm: 8.94 (d, J = 4.4 Hz, 1H), 8.21 – 8.15 (m, 1H), 8.15 – 8.09 (m, 2H), 8.05 (dd, J = 8.4, 0.7 Hz, 1H), 7.77 (ddd, J = 8.4, 6.9, 1.3 Hz, 1H), 7.67 – 7.61 (m, 1H), 7.61 – 7.57 (m, 1H), 7.55 (d, J = 4.4 Hz, 1H), 7.51 – 7.43 (m, 2H), 5.86 (s, 2H); ^{13}C NMR (100 MHz, $CDCl_3$) ppm: 166.13, 150.31, 148.23, 141.10, 133.45, 130.36, 129.80, 129.56, 129.53, 128.57, 127.15, 126.09, 123.03, 119.78, 63.11; CAS Number: 109091-29-4.

1-(pyrazin-2-yl) ethyl benzoate (3u)

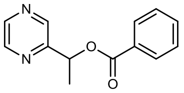

Isolated yield = 78%; colorless liquid; ^{1}H NMR (400 MHz, $CDCl_3$) ppm: 8.77 (s, 1H), 8.60 – 8.55 (m, 1H), 8.53 (d, J = 2.5 Hz, 1H), 8.11 (d, J = 7.1 Hz, 2H), 7.63 – 7.55 (m, 1H), 7.47 (t, J = 7.7 Hz, 2H), 6.23 (q, J = 6.7 Hz, 1H), 1.77 (d, J = 6.7 Hz, 3H); ^{13}C NMR (100 MHz, $CDCl_3$) ppm: 165.73, 155.80, 143.96, 142.71, 133.32, 129.77, 128.49, 71.73, 20.39; IR (KBr) ν_{max} 3062, 2985, 2935, 1720, 1601, 1584, 1269, 1099, and 712 cm^{-1}; HRMS (ESI) calcd. for $C_{13}H_{12}N_2O_2$: $[M+H]^+$: 229.0977, found: 229.0974.

2-(pyrazin-2-yl) ethyl benzoate (3v)

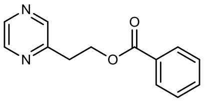

Isolated yield =79%; white liquid; ^{1}H NMR (400 MHz, $CDCl_3$) ppm: 8.57 (s, 1H), 8.56 – 8.53 (m, 1H), 8.46 (d, J = 2.4 Hz, 1H), 8.00 – 7.93 (m, 2H), 7.59 – 7.51 (m, 1H), 7.41 (t, J = 7.7 Hz, 2H), 4.73 (t, J = 6.5 Hz, 2H), 3.29 (t, J = 6.5 Hz, 2H); ^{13}C NMR (100 MHz, $CDCl_3$) ppm: 166.35, 154.00, 145.02, 144.36, 142.90, 133.07, 129.93, 129.54, 128.40, 63.39, 34.79; IR (KBr) ν_{max} 3061, 2966, 2925, 1717, 1602, 1583, 1274, 1113, and 711 cm^{-1}; HRMS (ESI) calcd. for $C_{13}H_{12}N_2O_2$: $[M+H]^+$: 229.0977, found: 229.0977.

2-(pyridin-2-yl) ethyl benzoate (3w)[5]

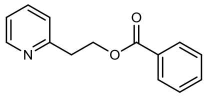

Isolated yield = 83%; white solid; ^{1}H NMR (400 MHz, $CDCl_3$) ppm: 8.57 (d, J = 4.2 Hz, 1H), 7.98 (d, J = 7.1 Hz, 2H), 7.63 (td, J = 7.7, 1.8 Hz, 1H), 7.53 (dd, J = 10.6, 4.3 Hz, 1H), 7.41 (t, J = 7.7 Hz, 2H), 7.25 (d, J = 7.8 Hz, 1H), 7.16 (dd, J = 7.0, 5.3 Hz, 1H), 4.72 (t, J = 6.7 Hz, 2H), 3.26 (t, J = 6.7 Hz, 2H; ^{13}C NMR (100 MHz, $CDCl_3$) ppm: 166.48, 158.11, 149.54, 136.46, 132.90, 130.24, 129.56, 128.33, 123.48, 121.70, 64.15, 37.53; CAS Number: 88733-27-1.

benzyl benzoate(3x)[9]

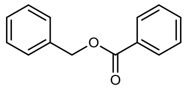

Isolated yield = 81%; colorless liquid; ^{1}H NMR (400 MHz, $CDCl_3$) ppm: 8.17 – 8.03 (m, 2H), 7.55 (t, J = 7.4 Hz, 1H), 7.49 – 7.40 (m, 4H), 7.38 (t, J = 7.3 Hz, 2H), 7.37 – 7.30 (m, 1H), 5.36 (s, 2H); ^{13}C NMR (100 MHz, $CDCl_3$) ppm: 166.44, 136.08, 133.04, 130.15, 129.72, 128.61, 128.39, 128.25, 128.18, 66.70; CAS Number: 120-51-4.

cyclohexylmethyl benzoate(3y)[9]

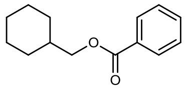

Isolated yield = 92%; light yello liquid; ^{1}H NMR (400 MHz, $CDCl_3$) ppm: 8.13 – 7.98 (m, 2H), 7.54 (t, J = 7.4 Hz, 1H), 7.43 (t, J = 7.6 Hz, 2H), 4.13 (d, J = 6.3 Hz, 2H), 1.91 – 1.64 (m, 6H), 1.38 – 1.17 (m, 3H), 1.07 (qd, J = 12.2, 2.6 Hz, 2H); ^{13}C NMR (100 MHz, $CDCl_3$) ppm: 166.68, 132.79, 130.57, 129.54, 128.32, 70.07, 37.29, 29.78, 26.40, 25.73; CAS Number: 14135-40-1.

butyl benzoate(3z)[9]

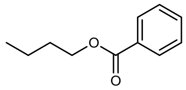

Isolated yield = 89%; colorless solid; ^{1}H NMR (400 MHz, $CDCl_3$) ppm: 8.05 (dd, J = 8.2, 1.0 Hz, 2H), 7.54 (t, J = 7.4 Hz, 1H), 7.43 (t, J = 7.7 Hz, 2H), 4.33 (t, J = 6.6 Hz, 2H), 1.76 (dt, J = 14.6, 6.7 Hz, 2H), 1.48 (dq, J = 14.7, 7.4 Hz, 2H), 0.98 (t, J = 7.4 Hz, 3H); ^{13}C NMR (100 MHz, $CDCl_3$) ppm: 166.64, 132.75, 130.52, 129.50, 128.28, 64.78, 30.76, 19.25, 13.72; CAS Number: 136-60-7.

pyrazin-2-ylmethyl 4-methoxybenzoate(4a)

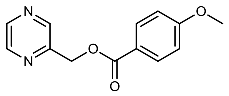

Isolated yield = 80%; light yellow solid; m. p. 112 – 113 °C; ^{1}H NMR (400 MHz, $CDCl_3$) ppm: 8.77 (d, J = 1.2 Hz, 1H), 8.59 (dd, J = 2.4, 1.6 Hz, 1H), 8.55 (d, J = 2.5 Hz, 1H), 8.06 (dt, J = 9.0, 2.8Hz, 2H), 6.94 (dt, J = 8.96, 2.8Hz, 2H), 5.50 (s, 2H), 3.87 (s, 3H); ^{13}C NMR (100 MHz, $CDCl_3$) ppm: 165.80, 163.76, 151.87, 144.09, 143.73, 131.92, 121.76, 113.79, 64.90, 55.48; IR (KBr) ν_{max} 2964, 2920, 1716, 1605, 1511, 1254, 1166, 1055, and 767 cm^{-1}; HRMS (ESI) calcd. for $C_{13}H_{12}N_2O_3$: $[M+H]^+$: 245.0926, found: 245.0923.

pyrazin-2-ylmethyl 4-fluorobenzoate (4b)

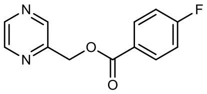

Isolated yield = 78%; deep yellow solid; m. p. 70 – 71 °C; ^{1}H NMR (400 MHz, $CDCl_3$) ppm: 8.77 (d, J = 0.7 Hz, 1H), 8.63 – 8.59 (m, 1H), 8.57 (d, J = 2.5 Hz, 1H), 8.17 – 8.09 (m, 2H), 7.18 – 7.10 (m, 2 H), 5.52 (s, 2H); ^{13}C NMR (100 MHz, $CDCl_3$) ppm: 167.32, 165.13, 164.78, 151.42, 144.24, 143.78, 132.49, 132.40, 125.66 (d, J = 2.95 Hz), 115.86, 115.64, 65.23; IR (KBr) ν_{max} 3051, 2923, 2852, 1725, 1599, 1509, 1290, 1126, and 765 cm^{-1}; HRMS (ESI) calcd. for $C_{12}H_9FN_2O_2$: [M+H]$^+$: 233.0726, found: 233.0724.

pyrazin-2-ylmethyl 4-chlorobenzoate (4c)

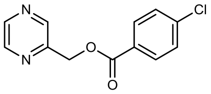

Isolated yield = 74%; light yellow solid; m. p. 85 – 86 °C; ^{1}H NMR (400 MHz, $CDCl_3$) ppm: 8.76 (s, 1H), 8.64-8.59 (m, 1H), 8.57 (d, J = 2.4 Hz, 1H), 8.07-8.01 (m, 2H), 7.44(dt, J =8.7, 2.3Hz, 2H), 5.52 (s, 2H); ^{13}C NMR (100 MHz, $CDCl_3$) ppm: 165.26, 151.31, 144.28, 144.26, 143.80, 139.99, 131.22, 128.92, 127.85, 65.31; IR (KBr) ν_{max} 3046, 2920, 2851, 1728, 1595, 1279, 1128, 1013, and 755 cm^{-1}; HRMS (ESI) calcd. for $C_{12}H_9ClN_2O_2$: [M+H]$^+$: 249.0431, found: 249.0430.

pyrazin-2-ylmethyl 4-bromobenzoate (4d)

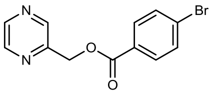

Isolated yield = 75%; light yellow solid; m. p. 93 – 94 °C; ^{1}H NMR (400 MHz, $CDCl_3$) ppm: 8.76 (d, J = 1.2 Hz, 1H), 8.63 – 8.59 (m, 1H), 8.57 (d, J = 2.5 Hz, 1H), 7.96 (dt, J = 8.8, 2.3 Hz, 2H), 7.61 (dt, J = 8.7 Hz, 2H), 5.52 (s, 2H); ^{13}C NMR (100 MHz, $CDCl_3$) ppm: 165.40, 151.28, 144.29, 144.26, 143.81, 131.91, 131.34, 128.68, 128.31, 65.32; IR (KBr) ν_{max} 2923, 2851, 1727, 1589, 1576, 1125, 1011, 990, and 752 cm^{-1}; HRMS (ESI) calcd. for $C_{12}H_9BrN_2O_2$: [M+H]$^+$: 292.9926, found: 292.9926.

pyrazin-2-ylmethyl 3-methylbenzoate (4e)

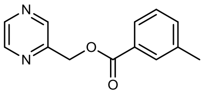

Isolated yield = 81%; yellow liquid; ^{1}H NMR (400 MHz, $CDCl_3$) ppm: 8.78 (s, 1H), 8.59 (d, J = 1.5 Hz, 1H), 8.56 (d, J = 2.5 Hz, 1H), 7.91 (d, J = 8.0 Hz, 2H), 7.41 (d, J = 7.9 Hz, 1H), 7.35 (t, J = 7.5 Hz, 1H), 5.52 (s, 2H), 2.41 (s, 3H); ^{13}C NMR (100 MHz, $CDCl_3$) ppm: 166.25, 151.67, 144.17, 144.13, 143.76, 138.37, 134.22, 130.34, 129.32, 128.43, 126.99, 65.10, 21.27; IR (KBr) ν_{max} 3009, 2924, 2851, 1722, 1593, 1573, 1278, 1106, and 755 cm^{-1}; HRMS (ESI) calcd. for $C_{13}H_{12}N_2O_2$: [M+H]$^+$: 229.0977, found: 229.0974.

pyrazin-2-ylmethyl 3-methoxybenzoate (4f)

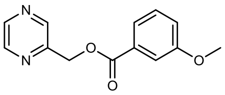

Isolated yield = 76%; deep yellow solid; m. p. 42 – 43 °C; ^{1}H NMR (400 MHz, $CDCl_3$) ppm: 8.78 (d, J = 0.4 Hz, 1H), 8.63 – 8.58 (m, 1H), 8.56 (d, J = 2.4 Hz, 1H), 7.70 (dt, J = 7.7, 1.1Hz, 1H), 7.62 (dd, J = 2.5, 1.6 Hz, 1H), 7.37 (t, J = 8.0 Hz, 1H), 7.14 (ddd, J = 8.3, 2.6, 0.8 Hz, 1H), 5.52 (s, 2H), 3.86 (s, 3H); ^{13}C NMR (100 MHz, $CDCl_3$) ppm: 165.98, 159.65, 151.57, 144.17, 143.76, 130.68, 129.57, 122.22, 119.96, 114.27, 65.23, 55.48; IR (KBr) ν_{max} 3057, 2996, 2933, 2839, 1721, 1600, 1278, 1118, and 755 cm^{-1}; HRMS (ESI) calcd. for $C_{13}H_{12}N_2O_3$: [M+H]$^+$: 245.0926, found: 245.0927.

pyrazin-2-ylmethyl 3-chlorobenzoate (4g)

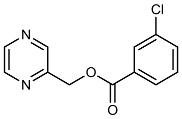

Isolated yield = 64%; yellow liquid; ^{1}H NMR (400 MHz, $CDCl_3$) ppm: 8.77 (s, 1H), 8.61 (s, 1H), 8.58 (s, 1H), 8.08 (s, 1H), 7.99 (d, J = 7.7 Hz, 1H), 7.57 (d, J = 7.7 Hz, 1H), 7.42 (t, J = 7.9 Hz, 1H), 5.53 (s, 2H); ^{13}C NMR (100 MHz, $CDCl_3$) ppm: 164.93, 151.18, 144.32, 144.28, 143.83, 134.72, 133.49, 131.14, 129.88, 127.97, 65.43; IR (KBr) ν_{max} 3073, 2924, 2852, 1728, 1577, 1282, 1263, 1014, and 740 cm^{-1}; HRMS (ESI) calcd. for $C_{12}H_9ClN_2O_2$: $[M+H]^+$: 249.0431, found: 249.0433.

pyrazin-2-ylmethyl 3-(trifluoromethyl) benzoate (4h)

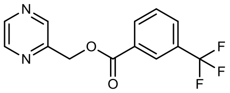

Isolated yield = 71%; yellow solid; m. p. 60 – 61 °C; ^{1}H NMR (400 MHz, $CDCl_3$) ppm: 8.78 (d, J = 1.0 Hz, 1H), 8.64 – 8.60 (m, 1H), 8.59 (d, J = 2.5 Hz, 1H), 8.36 (s, 1H), 8.29 (d, J = 7.8 Hz, 1H), 7.86 (d, J = 7.8 Hz, 1H), 7.62 (t, J = 7.8 Hz, 1H), 5.56 (s, 2H); ^{13}C NMR (100 MHz, $CDCl_3$) ppm: 164.85, 151.03, 144.35, 143.89, 133.04, 130.96 (q, J = 32.8 Hz), 130.30, 129.95 (q, J = 3.56 Hz), 129.80, 129.25, 126.75 (q, J = 3.7 Hz), 123.56 (q, J = 270.8 Hz), 65.55; IR (KBr) ν_{max} 3037, 2955, 1724, 1618, 1269, 1120, 1018, 1007, and 755 cm^{-1}; HRMS (ESI) calcd. for $C_{13}H_9F_3N_2O_2$: $[M+H]^+$: 283.0694, found: 283.0690.

pyridin-2-ylmethyl 3-methoxybenzoate (4i)

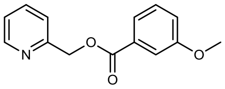

Isolated yield = 74%; deep yellow liquid; ^{1}H NMR (400 MHz, $CDCl_3$) ppm: 8.62 (dt, J = 4.18, 0.72 Hz, 1H), 7.76 – 7.68 (m, 2H), 7.63 (dd, J = 2.6, 1.5 Hz, 1H), 7.45 (d, J = 7.9 Hz, 1H), 7.36 (t, J = 8.0 Hz, 1H), 7.27 – 7.22 (m, 1H), 7.13 (ddd, J = 8.3, 2.7, 1.0 Hz, 1H), 5.49 (s, 2H), 3.85 (s, 3H); ^{13}C NMR (100 MHz, $CDCl_3$) ppm: 166.12, 159.60, 155.96, 149.46, 136.85, 131.12, 129.49, 122.90, 122.20, 121.70, 119.71, 114.28, 67.28, 55.47; IR (KBr) ν_{max} 3025, 2949, 2863, 1734, 1649, 1572, 1296, 1263, and 761cm^{-1}; HRMS (ESI) calcd. for $C_{14}H_{13}NO_3$: $[M+H]^+$: 244.0974, found: 244.0974.

pyridin-2-ylmethyl 3-chlorobenzoate (4j)

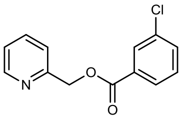

Isolated yield = 75%; yellow liquid; ^{1}H NMR (400 MHz, $CDCl_3$) ppm: 8.63 (dd, J = 4.8, 0.5 Hz, 1H), 8.09 (t, J = 1.8 Hz, 1H), 8.00 (dt, J = 7.8, 1.24Hz, 1H), 7.74 (td, J = 7.7, 1.8 Hz, 1H), 7.55 (ddd, J = 8.0, 2.1, 1.1 Hz, 1H), 7.44 (d, J = 7.8 Hz, 1H), 7.40 (t, J = 7.9 Hz, 1H), 7.30 – 7.22 (m, 1H), 5.49 (s, 2H); ^{13}C NMR (100 MHz, $CDCl_3$) ppm: 165.06, 155.55, 149.58, 136.90, 134.61, 133.25, 131.57, 129.85, 129.79, 127.96, 123.05, 121.88, 67.57; IR (KBr) ν_{max} 3070, 2926, 2852, 1726, 1594, 1573, 1280, 1073, and 749 cm^{-1}; HRMS (ESI) calcd. for $C_{13}H_{10}ClNO_2$: $[M+H]^+$: 248.0478, found: 248.0481.

pyridin-2-ylmethyl 3-bromobenzoate (4k)[6]

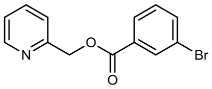

Isolated yield = 69%; yellow liquid; ^{1}H NMR (400 MHz, $CDCl_3$) ppm: 8.63 (dd, J = 4.8, 0.6 Hz, 1H), 8.24 (t, J = 1.7 Hz, 1H), 8.04 (dt, J = 7.8, 1.2 Hz, 1H), 7.77 – 7.68 (m, 2H), 7.44 (d, J = 7.8 Hz, 1H), 7.34 (t, J = 7.9 Hz, 1H), 7.29 – 7.24 (m, 1H), 5.48 (s, 2H); ^{13}C NMR (100 MHz, $CDCl_3$) ppm: 164.93, 155.53, 149.58, 136.90, 136.17, 132.75, 131.76, 130.03, 128.41, 123.06, 122.53, 121.90, 67.58; CAS Number: 501073-45-6.

pyridin-2-ylmethyl 3-(trifluoromethyl) benzoate (4l)

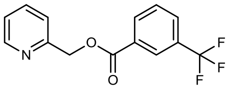

Isolated yield = 73%; yellow liquid; ^{1}H NMR (400 MHz, $CDCl_3$) ppm: 8.67 – 8.60 (m, 1H), 8.38 (s, 1H), 8.30 (d, J = 7.8 Hz, 1H), 7.84 (d, J = 7.8 Hz, 1H), 7.74 (td, J = 7.7, 1.8 Hz, 1H), 7.61 (t, J = 7.8 Hz, 1H), 7.45 (d, J = 7.8 Hz, 1H), 7.31 – 7.24(m,1H),5.52(s,2H); ^{13}CNMR (100 MHz, $CDCl_3$) ppm: 164.99, 155.40, 149.62, 136.92, 133.04, 133.99 (q, J = 32.81 Hz), 130.71, 129.72 (q, 3.57 Hz), 129. 15, 126.72 (q, J = 3.76 Hz), 123.62 (q, J = 270.91 Hz), 123.12, 121.98, 67.72; IR (KBr) ν_{max} 3073, 29 28, 1730, 1594, 1264, 1129, 1072, 1000, and 757 cm^{-1}; HRMS (ESI) calcd. for $C_{14}H_{10}F_3NO_2$: [M+H]$^+$: 282.0742, found: 282.0742.

pyridin-2-ylmethyl 4-methoxybenzoate (4m)[6]

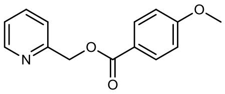

Isolated yield = 73%; light yellow solid; ^{1}H NMR (400 MHz, $CDCl_3$) ppm: 8.61 (d, J = 4.2 Hz, 0H), 8.08 (dt, J = 9.4, 2.8 Hz, 2H), 7.71 (td, J = 7.7, 1.8 Hz, 1H), 7.44 (d, J = 7.8 Hz, 1H), 7.30 – 7.17 (m, 1H), 6.94 (dt, J = 9.0, 2.8 Hz, 2H), 5.46 (s, 2H), 3.87 (s, 3H); ^{13}C NMR (100 MHz, $CDCl_3$) ppm: 165. 93, 163.53, 156.20, 149.36, 136.78, 131.82, 122.77, 122.17, 121.61, 113.65, 66.87, 55.41; CAS Num ber: 70415-72-4.

pyridin-2-ylmethyl 4-chlorobenzoate (4n)[1]

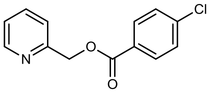

Isolated yield = 76%; deep yellow liquid; ^{1}H NMR (400 MHz, $CDCl_3$) ppm: 8.65 – 8.60 (m, 1H), 8.07 – 8.03 (m, 2H), 7.73 (td, J = 7.7, 1.8 Hz, 1H), 7.47 – 7.39 (m, 3H), 7.29 – 7.21 (m, 1H), 5.48 (s, 2H); ^{13}C NMR (100 MHz, $CDCl_3$) ppm: 165.37, 155.63, 149.51, 139.67, 136.85, 131.17, 128.78, 128.25, 122.98, 121.82, 67.38; CAS Number: 760191-96-6.

pyridin-2-ylmethyl 4-bromobenzoate (4o)[1]

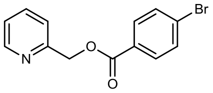

Isolated yield = 75%; yellow liquid; ^{1}H NMR (400 MHz, $CDCl_3$) ppm: 8.67 – 8.57 (m, 1H), 7.97 (dt, J = 8.68,2.24Hz,2H), 7.73 (td, J = 7.7, 1.8 Hz, 1H), 7.60 (dt, J = 8.68,2.28,2H), 7.43 (d, J = 7.8 Hz, 1 H), 7.30 – 7.24 (m, 1H), 5.48 (s, 2H); ^{13}C NMR (100 MHz, $CDCl_3$) ppm: 165.54, 155.65, 149.56, 13 6.87, 131.81, 131.33, 128.74, 128.39, 123.02, 121.85, 67.45; CAS Number: 1624211-68-2.

pyridin-4-ylmethyl 3-methoxybenzoate (4p)

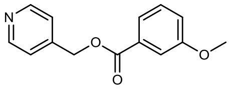

Isolated yield = 76%; deep yellow liquid; ^{1}H NMR (400 MHz, $CDCl_3$) ppm: 8.62 (dd, J = 4.5, 1.5 Hz, 2H), 7.70 (dt, J = 7.7, 1.2 Hz, 1H), 7.61 (dd, J = 2.5, 1.6 Hz, 1H), 7.38 (t, J = 8.0 Hz, 1H), 7.34 (d, J = 6.0 Hz, 2H), 7.14 (ddd, J = 8.3, 2.7, 0.8 Hz, 1H), 5.38 (s, 2H), 3.86 (s, 3H); ^{13}C NMR (100 MHz, $CDCl_3$) ppm: 165.98, 159.67, 150.05, 145.06, 130.78, 129.60, 122.10, 121.85, 119.79, 114.34, 64.72, 55.48; IR (KBr) ν_{max} 3031, 2942, 2836, 1723, 1603, 1562, 1276, 1226, and 754 cm^{-1}; HRMS (ESI) calcd. for $C_{14}H_{13}NO_3$: [M+H]$^+$: 244.0974, found: 244.0975.

pyridin-4-ylmethyl 3-chlorobenzoate (4q)

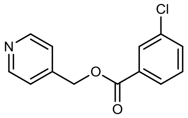

Isolated yield =74%; colorless liquid; ^{1}H NMR (400 MHz, $CDCl_3$) ppm: 8.64 (dd, J = 4.5, 1.5 Hz, 2 H), 8.07 (t, J = 1.8 Hz, 1H), 7.98 (dt, J = 7.8, 1.2 Hz, 1H), 7.58 (ddd, J = 8.0, 2.1, 1.1 Hz, 1H), 7.42 (t, J = 7.9 Hz, 1H), 7.34 (d, J = 6.0 Hz, 2H), 5.38 (s, 2H); ^{13}C NMR (100 MHz, $CDCl_3$) ppm: 164.91, 1 50.15, 144.63, 134.74, 133.48, 131.23, 129.90, 129.79, 127.88, 121.93, 65.03; IR (KBr) ν_{max} 3073, 29 24, 2850, 1726, 1604, 1574, 1256, 1127, 993, and 749 cm^{-1}; HRMS (ESI) calcd. for $C_{13}H_{10}ClNO_2$: $[M+H]^+$: 248.0478, found: 248.0472.

pyridin-4-ylmethyl 4-methoxybenzoate (4r)

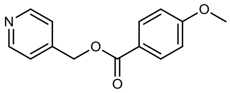

Isolated yield = 85%; light yellow solid; m. p. 94 – 95 °C; ^{1}H NMR (400 MHz, $CDCl_3$) ppm: 8.62 (d d, J = 4.5, 1.5 Hz, 2H), 8.06 (dt, J = 9.0, 3.0 Hz, 2H), 7.33 (d, J = 6.0 Hz, 2H), 6.95 (dt, J = 9.7, 2.8 H z, 2H), 5.35 (s, 2H), 3.87 (s, 3H); ^{13}C NMR (100 MHz, $CDCl_3$) ppm: 165.81, 163.75, 150.03, 145.36, 131.82, 121.86, 121.83, 113.81, 64.36, 55.48; IR (KBr) ν_{max} 3042, 2957, 2852, 1725, 1688, 1599, 126 6, 1229, and 756cm^{-1}; HRMS (ESI) calcd. for $C_{14}H_{13}NO_3$: $[M+H]^+$: 244.0974, found: 244.0971.

pyridin-4-ylmethyl 4-chlorobenzoate (4s)

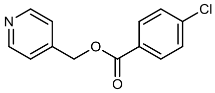

Isolated yield = 84%; light yellow solid; m. p. 82 – 83 °C; ^{1}H NMR (400 MHz, $CDCl_3$) ppm: 8.64 (d, J = 5.9 Hz, 2H), 8.03 (dt, J = 9.0, 2.2 Hz, 2H), 7.49 – 7.42 (m, J = 9.0, 2.2 Hz, 2H), 7.33 (d, J = 5.9 Hz, 2H), 5.37 (s, 2H); ^{13}C NMR (100 MHz, $CDCl_3$) ppm: 165.24, 150.12, 144.77, 139.97, 131.13, 128.93, 127.94, 121.90, 64.88, 29.69; IR (KBr) ν_{max} 3033, 2918, 2849, 1721, 1605, 1592, 1271, 1086, 1009, and 757 cm^{-1}; HRMS (ESI) calcd. for $C_{13}H_{10}ClNO_2$: $[M+H]^+$: 248.0478, found: 248.0476.

pyridin-4-ylmethyl 4-bromobenzoate (4t)

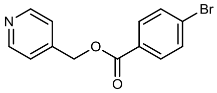

Isolated yield = 85%; yellow solid; m. p. 77 – 78 °C; ^{1}H NMR (400 MHz, $CDCl_3$) ppm: 8.64 (d, J = 5.7 Hz, 2H), 7.96 (dt, J = 9.0, 2.2 Hz, 2H), 7.67 – 7.51 (m, 2H), 7.33 (d, J = 5.9 Hz, 2H), 5.37 (s, 2H); 13C NMR (100 MHz, $CDCl_3$) ppm: 165.38, 150.12, 144.73, 131.93, 131.24, 128.65, 128.40, 121.90, 64.90; IR (KBr) ν_{max} 2970, 2923, 1723, 1605, 1588, 1274, 1124, 1007, and 757 cm^{-1}; HRMS (ESI) ca lcd. for $C_{13}H_{10}BrNO_2$: $[M+H]^+$: 291.9973, found: 291.9973.

furan-2-ylmethyl 4-methoxybenzoate (4u)[7]

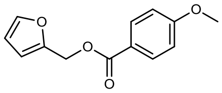

Isolated yield = 89%; yellow liquid; ^{1}H NMR (400 MHz, $CDCl_3$) ppm: 8.00 (dt, J = 9.7, 2.8 Hz, 2H), 7.44 (dd, J = 1.8, 0.8 Hz, 1H), 6.90 (dt, J = 9.7, 2.8 Hz, 2H), 6.47 (d, J = 3.2 Hz, 1H), 6.38 (dd, J = 3. 2, 1.9 Hz, 1H), 5.28 (s, 2H), 3.85 (s, 3H); ^{13}C NMR (100 MHz, $CDCl_3$) ppm: 166.01, 163.49, 149.80, 143.22, 131.82, 122.30, 113.61, 110.62, 110.57, 58.27, 55.43; CAS Number: 182230-12-2.

furan-2-ylmethyl 4-chlorobenzoate (4v)[7]

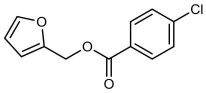

Isolated yield = 78%; deep yellow liquid; ^{1}H NMR (400 MHz, $CDCl_3$) ppm: 7.98 (dt, J = 9.0, 2.3 Hz, 2H), 7.49 – 7.43 (m, 1H), 7.40 (dt, J = 8.6, 2.3 Hz, 2H), 6.49 (d, J = 3.2 Hz, 1H), 6.39 (dd, J = 3.2,

1.9 Hz, 1H), 5.30 (s, 2H); ^{13}C NMR (100 MHz, $CDCl_3$) ppm: 165.41, 149.30, 143.42, 139.58, 131.16, 128.72, 128.36, 110.98, 110.64, 58.71; CAS Number: 4449-28-9.

furan-2-ylmethyl 4-bromobenzoate (4w)

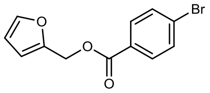

Isolated yield = 72%; light yellow solid; ^{1}H NMR (400 MHz, $CDCl_3$) ppm: 7.91 (dt, *J* = 9.0, 2.3 Hz, 2H), 7.56 (dt, *J* = 9.0, 2.3 Hz, 2H), 7.45 (dd, *J* = 1.8, 0.8 Hz, 1H), 6.49 (d, *J* = 3.2 Hz, 1H), 6.39 (dd, *J* = 3.2, 1.9 Hz, 1H), 5.30 (s, 2H); ^{13}C NMR (100 MHz, $CDCl_3$) ppm: 165.55, 149.27, 143.43, 131.72, 131.29, 128.81, 128.26, 111.00, 110.64, 58.73; IR (KBr) ν_{max} 2962, 2925, 2851, 1721, 1590, 1269, 11 00, 1012, and 755 cm^{-1}; HRMS (ESI) calcd. for $C_{12}H_9BrO_3$: [M+H]$^+$: 280.9813, found: 280.9810.

thiophen-2-ylmethyl 3-chlorobenzoate (4x)

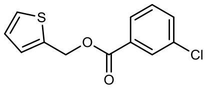

Isolated yield = 90%; colorless liquid; ^{1}H NMR (400 MHz, $CDCl_3$) ppm: 8.02 (t, *J* = 1.8 Hz, 1H), 7.9 4 (dt, *J* = 6.6, 1.2 Hz, 1H), 7.52 (ddd, *J* = 8.0, 2.1, 1.1 Hz, 1H), 7.42 – 7.32 (m, 2H), 7.18 (d, *J* = 3.3 Hz, 1H), 7.01 (dd, *J* = 5.1, 3.5 Hz, 1H), 5.51 (s, 2H); ^{13}C NMR (100 MHz, $CDCl_3$) ppm: 165.09, 137. 55, 134.56, 133.16, 131.68, 129.80, 129.72, 128.51, 127.89, 127.10, 126.92, 61.36; IR (KBr) ν_{max} 307 2, 2924, 1723, 1595, 1574, 1277, 1252, 1071, and 748 cm^{-1}; HRMS (ESI) calcd. for $C_{12}H_9ClO_2S$: [M +H]$^+$: 253.0090, found: 253.0089.

thiophen-2-ylmethyl 3-bromobenzoate (4y)

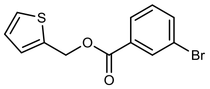

Isolated yield = 86%; colorless liquid; ^{1}H NMR (400 MHz, $CDCl_3$) ppm: 8.18 (t, *J* = 1.7 Hz, 1H), 7.9 8 (dt, *J* = 7.8, 1.2 Hz, 1H), 7.67 (ddd, *J* = 8.0, 2.0, 1.1 Hz, 1H), 7.35 (dd, *J* = 5.1, 1.2 Hz, 1H), 7.30 (t, *J* = 7.9 Hz, 1H), 7.20 – 7.16 (m, 1H), 7.01 (dd, *J* = 5.1, 3.5 Hz, 1H), 5.51 (s, 2H); ^{13}C NMR (100 M Hz, $CDCl_3$) ppm: 164.96, 137.54, 136.08, 132.72, 131.87, 129.97, 128.53, 128.35, 127.12, 126.93, 12 2.49, 61.37; IR (KBr) ν_{max} 3069, 2953, 1721, 1570, 1278, 1252, 1067, 940, and 745 cm^{-1}; HRMS (ESI) calcd. for $C_{12}H_9BrO_2S$: [M+H]$^+$: 296.9585, found: 296.9588.

thiophen-2-ylmethyl 4-methoxybenzoate(4z)[8]

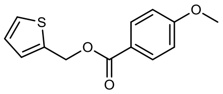

Isolated yield =81%; colorless liquid; ^{1}H NMR (400 MHz, $CDCl_3$) ppm: 8.01 (dt, *J* = 9.0, 2.8 Hz, 2H), 7.33 (dd, *J* = 5.1, 1.2 Hz, 1H), 7.16 (dd, *J* = 3.2, 0.7 Hz, 1H), 7.00 (dd, *J* = 5.1, 3.5 Hz, 1H), 6.90 (dt, *J* = 9.7, 2.8 Hz, 2H), 5.48 (s, 2H), 3.84 (s, 3H); ^{13}C NMR (100 MHz, $CDCl_3$) ppm: 166.02, 163.52, 138.33, 131.82, 128.01, 126.81, 126.74, 122.33, 113.64, 60.81, 55.44; CAS Number:1387436-20-5.

thiophen-2-ylmethyl 4-chlorobenzoate (4a')

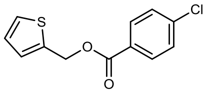

Isolated yield = 84%; colorless liquid; ^{1}H NMR (400 MHz, $CDCl_3$) ppm: 7.98 (dt, *J* = 9.0, 2.3 Hz, 2H), 7.40 (dt, *J* = 19.0, 2.3 Hz, 2H), 7.34 (dd, *J* = 5.1, 1.2 Hz, 1H), 7.17 (dd, *J* = 3.2, 0.7 Hz, 1H), 7.01 (dd, *J* = 5.1, 3.5 Hz, 1H), 5.50 (s, 2H); ^{13}C NMR (100 MHz, $CDCl_3$) ppm: 165.42, 139.61, 137.71, 131.15, 128.75, 128.39, 127.03, 126.90, 61.25; IR (KBr) ν_{max} 2972, 2900, 1722, 1644, 1606,

1278, 1227, 1045, and 754 cm^{-1}; HRMS (ESI) calcd. for $C_{12}H_9ClO_2S$: $[M+H]^+$: 253.0090, found: 253.0088.

thiophen-2-ylmethyl 4-bromobenzoate (4b')

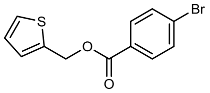

Isolated yield = 89%; white solid; m. p. 70 – 71 ºC; ^1H NMR (400 MHz, $CDCl_3$) ppm: 7.91 (dt, J = 9.0, 2.2 Hz, 2H), 7.56 (dt, J = 8.7, 2.2 Hz, 2H), 7.34 (dd, J = 5.1, 1.2 Hz, 1H), 7.17 (dt, J = 3.5, 0.5 Hz, 1H), 7.01 (dd, J = 5.1, 3.5 Hz, 1H), 5.50 (s, 2H); ^{13}C NMR (100 MHz, $CDCl_3$) ppm: 165.56, 137.69, 131.75, 131.28, 128.84, 128.40, 128.30, 127.04, 126.91, 61.27, 29.72; IR (KBr) ν_{max} 3094, 2958, 2922, 2851, 1707, 1588, 1281, 1093, 1010, and 760 cm^{-1}; HRMS (ESI) calcd. for $C_{12}H_9BrO_2S$: $[M+H]^+$: 296.9585, found: 296.9585.

pyrazin-2-ylmethyl pivalate (4c')

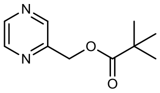

Isolated yield = 89%; yellow liquid; ^1H NMR (400 MHz, $CDCl_3$) ppm: 8.67 – 8.64 (m, 1H), 8.57 – 8.55 (m, 1H), 8.54 (d, J = 2.5 Hz, 1H), 5.28 (s, 2H), 1.28 (s, 9H); ^{13}C NMR (100 MHz, $CDCl_3$) ppm: 177.94, 151.95, 144.03, 143.91, 143.30, 64.66, 38.86, 27.16; IR (KBr) ν_{max} 2975, 2936, 2874, 1736, 1480, 1283, 1144, 1018, and 832 cm^{-1}; HRMS (ESI) calcd. for $C_{10}H_{14}N_2O_2$: $[M+H]^+$: 195.1134, found: 195.1139.

pyrazin-2-ylmethyl 3-bromobenzoate (5a)

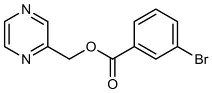

Isolated yield = 80%; yellow solid; m. p. 89 – 90 ºC; ^1H NMR (400 MHz, $CDCl_3$) ppm: 8.77 (s, 1H), 8.63 – 8.59 (m, 1H), 8.58 (d, J = 2.5 Hz, 1H), 8.23 (t, J = 1.7 Hz, 1H), 8.03 (dt, J = 7.8, 1.2 Hz, 1H), 7.72 (ddd, J = 8.0, 2.0, 1.1 Hz, 1H), 7.35 (t, J = 7.9 Hz, 1H), 5.53 (s, 2H); ^{13}C NMR (100 MHz, $CDCl_3$) ppm: 164.80, 151.17, 144.33, 144.28, 143.84, 136.40, 132.79, 131.33, 130.11, 128.42, 122.61, 65.44; IR (KBr) ν_{max} 3069, 2927, 1728, 1685, 1572, 1407, 1282, 1057, and 739 cm^{-1}; HRMS (ESI) calcd. for $C_{12}H_9BrN_2O_2$: $[M+H]^+$: 292.9926, found: 292.9922.

pyrazin-2-ylmethyl 2,3-dichlorobenzoate(5b)

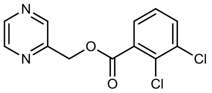

Isolated yield = 62%; yellow liquid; ^1H NMR (400 MHz, $CDCl_3$) ppm: 8.79 (d, J = 1.0 Hz, 1H), 8.64 – 8.60 (m, 1H), 8.58 (d, J = 2.5 Hz, 1H), 7.76 (dd, J = 7.8, 1.6 Hz, 1H), 7.63 (dd, J = 8.0, 1.6 Hz, 1H), 7.33 – 7.26 (m, 1H), 5.54 (s, 2H); ^{13}C NMR (100 MHz, $CDCl_3$) ppm: 164.78, 150.86, 144.35, 144.27, 143.85, 134.83, 133.65, 131.99, 131.92, 129.43, 127.25, 65.86; IR (KBr) ν_{max} 3075, 2924, 1737, 1582, 1286, 1257, 1055, 1019, and 756 cm^{-1}; HRMS (ESI) calcd. for $C_{12}H_8Cl_2N_2O_2$: $[M+H]^+$: 283.0041, found: 283.0040.

(5-methylpyridin-2-yl) methyl 2-methoxybenzoate (5c)

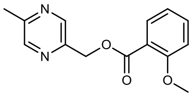

Isolated yield = 55%; light yellow liquid; ^1H NMR (400 MHz, $CDCl_3$) ppm: 8.69 (d, J = 0.8 Hz, 1H), 8.45 (d, J = 0.6 Hz, 1H), 7.89 (dd, J = 8.0, 1.8 Hz, 1H), 7.53 – 7.46 (m, 1H), 6.99 (dd, J = 10.8, 4.5 Hz, 2H), 5.46 (s, 2H), 3.93 (s, 3H), 2.59 (s, 3H); ^{13}C NMR (100 MHz, $CDCl_3$) ppm: 165.68, 159.48, 153.12, 148.47, 143.77, 142.70, 134.08, 132.04, 120.20, 119.14, 112.02, 65.00, 55.96, 21.36; IR

(KBr) ν_{max} 2927, 2840, 1730, 1601, 1301, 1255, 1130, 1081, and 757 cm^{-1}; HRMS (ESI) calcd. for $C_{14}H_{14}N_2O_3$: $[M+H]^+$: 259.1083, found: 259.1081.

(6-methoxypyridin-2-yl) methyl 2-methylbenzoate (5d)

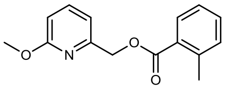

Isolated yield = 74%; colorless solid; m. p. 49 – 50 °C; ^{1}H NMR (400 MHz, $CDCl_3$) ppm: 8.03 (dd, J = 8.1, 1.3 Hz, 1H), 7.56 (dd, J = 8.2, 7.3 Hz, 1H), 7.41 (td, J = 7.5, 1.4 Hz, 1H), 7.28 – 7.23 (m, 2H), 6.97 (dd, J = 7.2, 0.5 Hz, 1H), 6.69 – 6.64 (m, 1H), 5.36 (s, 2H), 3.92 (s, 3H), 2.63 (s, 3H); ^{13}C NMR (100 MHz, $CDCl_3$) ppm: 167.16, 163.80, 153.69, 140.50, 139.05, 132.17, 131.76, 130.77, 129.37, 12 5.77, 113.89, 109.86, 77.38, 77.06, 76.74, 66.67, 53.39, 21.82; IR (KBr) ν_{max} 3006, 2951, 2927, 1720, 1265, 1248, 1151, 1095, and 739 cm^{-1}; HRMS (ESI) calcd. for $C_{15}H_{15}NO_3$: $[M+H]^+$: 258.1130, found: 258.1127.

(3-methylpyridin-2-yl) methyl 2-methoxybenzoate (5e)

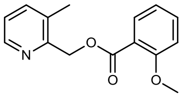

Isolated yield = 45%; colorless liquid; ^{1}H NMR (400 MHz, $CDCl_3$) ppm: 8.45 (d, J = 4.7 Hz, 1H), 7. 84 (dd, J = 7.7, 1.7 Hz, 1H), 7.51 (d, J = 7.0 Hz, 1H), 7.49 – 7.43 (m, 1H), 7.19 (dd, J = 7.6, 4.8 Hz, 1 H), 6.96 (dd, J = 12.5, 5.2 Hz, 2H), 5.46 (s, 2H), 3.88 (s, 3H), 2.44 (s, 3H); ^{13}C NMR (100 MHz, CD Cl_3) ppm: 165.78, 159.34, 153.57, 146.85, 138.25, 133.65, 132.85, 131.86, 123.43, 120.10, 119.71, 1 11.96, 66.38, 55.92, 18.13; IR (KBr) ν_{max} 3054, 3002, 2925, 1726, 1300, 1254, 1049, 1023, and 757 c m^{-1}; HRMS (ESI) calcd. for $C_{15}H_{15}NO_3$: $[M+H]^+$: 258.1130, found: 258.1130.

(6-methylpyrazin-2-yl) methyl 2-methoxybenzoate (5f)

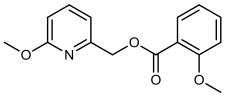

Isolated yield = 41%, light yellow liquid; ^{1}H NMR (400 MHz, $CDCl_3$) ppm: 7.91 (dd, J = 7.9, 1.8 Hz, 1H), 7.57 (dd, J = 8.2, 7.4 Hz, 1H), 7.50 (ddd, J = 8.5, 7.5, 1.8 Hz, 1H), 7.03 (dd, J = 7.3, 0.6 Hz, 1 H), 7.02 – 6.97 (m, 2H), 6.66 (d, J = 8.3 Hz, 1H), 5.36 (s, 2H), 3.93 (s, 6H);^{13}C NMR (101 MHz, CD Cl_3) ppm: 165.71, 163.70, 159.44, 153.88, 139.03, 133.81, 131.92, 120.18, 119.73, 113.75, 112.06, 1 09.67, 66.67, 55.98, 53.40; IR (KBr) ν_{max} 3056, 2946, 2936, 2917, 1297, 1252, 1011, 1046, and 759 c m^{-1}; HRMS (ESI) calcd. for $C_{15}H_{15}NO_4$: $[M+H]^+$: 274.1079, found: 274.1074.

pyridin-2-ylmethyl 2,3-dichlorobenzoate (5g)

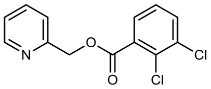

Isolated yield = 60%; light yellow liquid; ^{1}H NMR (400 MHz, $CDCl_3$) ppm: 8.62 (dd, J = 4.8, 0.6 Hz, 1H), 7.79 – 7.69 (m, 2H), 7.61 (dd, J = 8.0, 1.6 Hz, 1H), 7.47 (d, J = 7.8 Hz, 1H), 7.32 – 7.22 (m, 2 H), 5.50 (s, 2H); ^{13}C NMR (100 MHz, $CDCl_3$) ppm: 164.96, 155.16, 149.56, 136.92, 134.67, 133.38, 132.42, 131.84, 129.38, 127.21, 123.11, 122.01, 68.03; IR (KBr) ν_{max} 3068, 3013, 2929, 1736, 1593, 1 573, 1286, 1049, and 756 cm^{-1}; HRMS (ESI) calcd. for $C_{13}H_9Cl_2NO_2$: $[M+H]^+$: 282.0089, found: 282. 0087.

pyridin-3-ylmethyl 2-methoxybenzoate (5h)

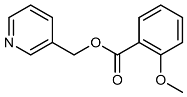

Isolated yield = 52%; white liquid; ^{1}H NMR (400 MHz, $CDCl_3$) ppm: 8.73 (d, J = 1.7 Hz, 1H), 8.59 (dd, J = 4.8, 1.5 Hz, 1H), 7.83 (dd, J = 8.0, 1.8 Hz, 1H), 7.80 (dt, J = 7.8, 1.9 Hz, 1H), 7.52 – 7.46 (m,

1H), 7.32 (dd, J = 7.8, 4.9 Hz, 1H), 6.98 (dd, J = 11.5, 4.4 Hz, 2H), 5.37 (s, 2H), 3.91 (s, 3H); ^{13}C NMR (100 MHz, $CDCl_3$) ppm: 165.79, 159.44, 149.57, 149.48, 135.90, 133.97, 131.91, 131.82, 123.44, 120.18, 119.36, 112.06, 63.96, 55.97; IR (KBr) ν_{max} 3032, 2924, 1726, 1600, 1300, 1250, 1129, 1075, and 757 cm^{-1}; HRMS (ESI) calcd. for $C_{14}H_{13}NO_3$: $[M+H]^+$: 244.0974, found: 244.0971.

furan-3-ylmethyl 2-methoxybenzoate (5i)

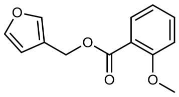

Isolated yield =60%; light yellow liquid; ^{1}H NMR (400 MHz, $CDCl_3$) ppm: 7.80 (dd, J = 7.7, 1.7 Hz, 1H), 7.50 – 7.40 (m, 2H), 6.98 – 6.92 (m, 2H), 6.47 (d, J = 3.2 Hz, 1H), 6.37 (dd, J = 3.2, 1.9 Hz, 1H), 5.28 (s, 2H), 3.89 (s, 3H); ^{13}C NMR (100 MHz, $CDCl_3$) ppm: 165.55, 159.44, 149.71, 143.19, 133.77, 131.83, 120.09, 119.55, 112.04, 110.65, 110.58, 58.37, 56.00; IR (KBr) ν_{max} 2951, 2924, 1720, 1600, 1298, 1244, 1068, 1048, and 756 cm^{-1}; HRMS (ESI) calcd. for $C_{13}H_{12}O_4$: $[M+H]^+$: 233.0814, found: 233.0814.

thiophen-3-ylmethyl 2-methoxybenzoate (5j)

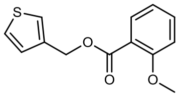

Isolated yield = 51%; light yellow liquid; ^{1}H NMR (400 MHz, $CDCl_3$) ppm: 7.81 (dd, J = 8.0, 1.8 Hz, 1H), 7.46 (ddd, J = 8.5, 7.5, 1.8 Hz, 1H), 7.32 (dd, J = 5.1, 1.2 Hz, 1H), 7.16 (dt, J = 3.5, 0.6 Hz, 1H), 6.99 (dd, J = 5.1, 3.5 Hz, 1H), 6.95 (dd, J = 11.7, 4.3 Hz, 2H), 5.49 (s, 2H), 3.90 (s, 3H); ^{13}C NMR (100 MHz, $CDCl_3$) ppm: 165.63, 159.47, 138.25, 133.80, 131.86, 128.02, 126.78, 126.69, 120.11, 119.57, 112.05, 60.92, 56.00; IR (KBr) ν_{max} 3107, 2924, 2838, 1725, 1600, 1298, 1244, 1068, and 756 cm^{-1}; HRMS (ESI) calcd. for $C_{13}H_{12}O_3S$: $[M+H]^+$: 249.0585, found: 249.0583.

pyrazin-2-ylmethyl 1-naphthoate (5k)

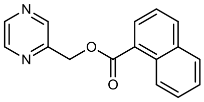

Isolated yield = 60%; yellow liquid; ^{1}H NMR (400 MHz, $CDCl_3$) ppm: 8.97 (d, J = 8.7 Hz, 1H), 8.82 (s, 1H), 8.59 (s, 1H), 8.55 (d, J = 2.1 Hz, 1H), 8.30 (d, J = 7.2 Hz, 1H), 8.04 (d, J = 8.2 Hz, 1H), 7.88 (d, J = 8.1 Hz, 1H), 7.62 (t, J = 7.4 Hz, 1H), 7.52 (dt, J = 15.3, 7.7 Hz, 2H), 5.60 (s, 2H); ^{13}C NMR (100 MHz, $CDCl_3$) ppm: 166.78, 151.68, 144.23, 144.18, 143.83, 134.01, 133.87, 131.49, 130.72, 128.65, 128.06, 126.37, 126.06, 125.70, 124.50, 65.19; IR (KBr) ν_{max} 3050, 3013, 2919, 1717, 1622, 1248, 1131, and 782 cm^{-1}; HRMS (ESI) calcd. for $C_{16}H_{12}N_2O_2$: $[M+H]^+$: 265.0977, found: 265.0974.

5 References

1. C.-S. Wang, T. Roisnel, P. H. Dixneuf, J.-F. Soulé, Synthesis of 2-pyridinemethyl ester derivatives from aldehydes and 2-alkylheterocycle N-oxides via copper-catalyzed tandem oxidative coupling-rearrangement, *Org. Lett.*, **2017**, *19*, 6720-6723.
2. M. N. Rashed , S. M. A. H. Siddiki, A. S. Touchy, M. A. R. Jamil, S. S. Poly, T. Toyao, Z. Maeno, K. Shimizu, Direct phenolysis reactions of unactivated amides into phenolic esters promoted by a heterogeneous CeO_2 catalyst, *Chem. Eur. J.*, **2019**, *25*, 10594-10605.
3. J. Dong, X. Chen, F. Ji, L. Liu, L. Su, M. Mo, J.-S. Tang, Y. Zhou, Copper-mediated simple and direct aerobic oxidative esterification of arylacetonitriles with alcohols/phenols, *Appl. Organomet. Chem.,* **2021**, *35*, 6073-6083.0
4. A. Boutros, J.-Y. Legros, J.-C. Fiaud, 4-Quinolylmethyl and 1-Naphthylmethyl as benzyl-type protecting groups of carboxylic acids removable by homogeneous palladium-catalyzed hydrogenolysis, *Tetrahedron*, **2000**, *56*, 2239-2246.

5. A. R. Katritzky, G. R. Khan, O. A. Schwarz, Pyridylethylation- a new protection method for active hydrogen compounds, *Tetrahedron Lett.*, **1984**, *25*, 1223-1226.
6. S. Ko, C. Lee, M.-G. Choi, Y. Na, S. Chang, Chelation-accelerated sequential decarbonylation of formate and alkoxycarbonylation of aryl halides using a combined Ru and Pd catalyst, *J. Org. Chem*., **2003**, *68*, 1607-1610.
7. X. Qi, R. Zhou, H.-J. Ai, X.-F. Wu, HMF and furfural: Promising platform molecules in rhodium-catalyzed carbonylation reactions for the synthesis of furfuryl esters and tertiary amides, *J. Catal.*, **2020**, *381*, 215-221.
8. Y. Bourne-Branchu, C. Gosmini, G. Danoun, Cobalt-catalyzed esterification of amides, *Chem. Eur. J.*, **2017**, *23*, 10043-10047.
9. M. Subaramanian, M. R. Palmurukan, J. Rana, Catalytic conversion of ketones to esters via C (O)-C bond cleavage under transition-metal free conditions, *Chem Commun.*, **2020**, *56*, 8143-8146.

6 Copy of ^{1}H and ^{13}C NMR Spectra

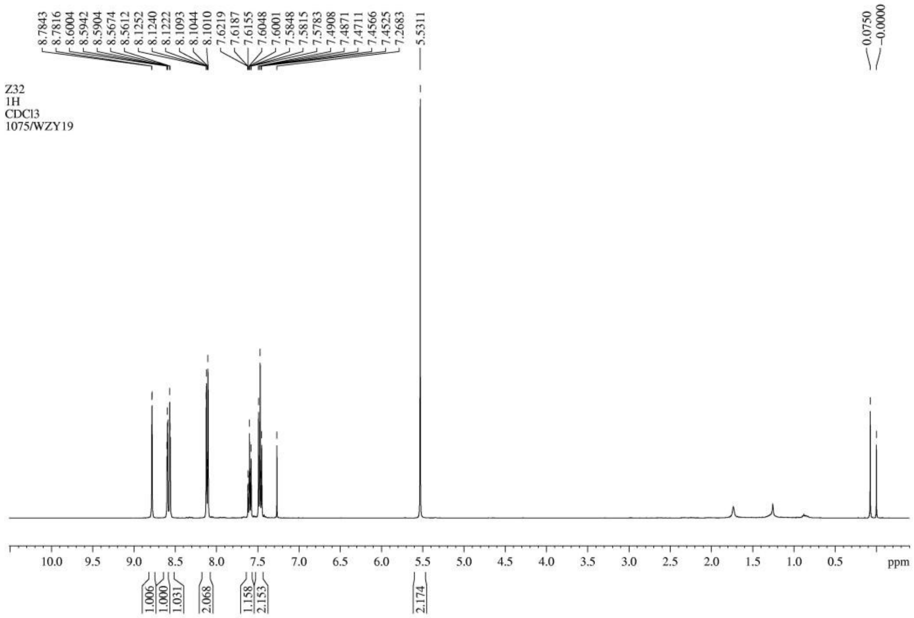

^{1}H NMR spectrum of compound **3a**

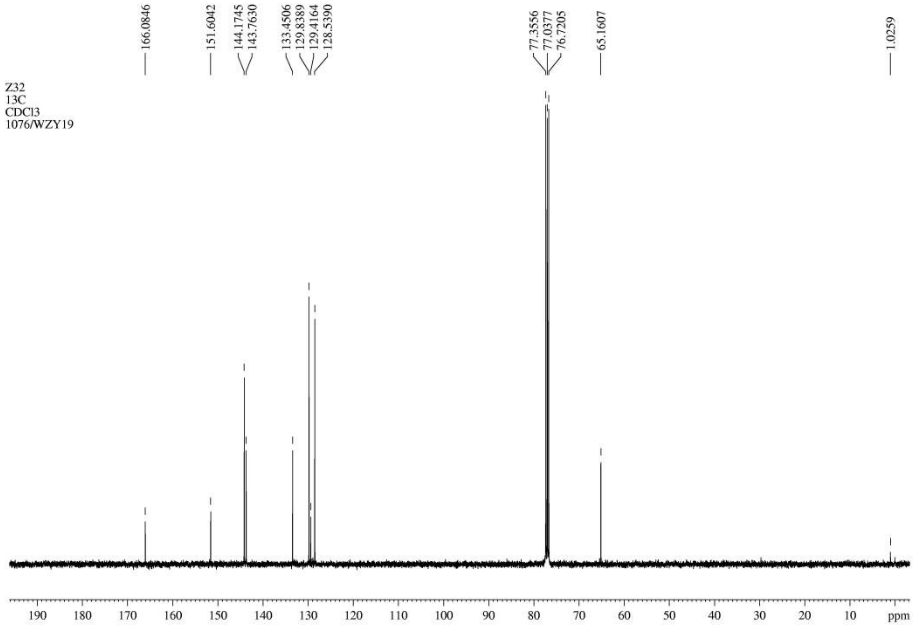

^{13}C NMR spectrum of compound **3a**

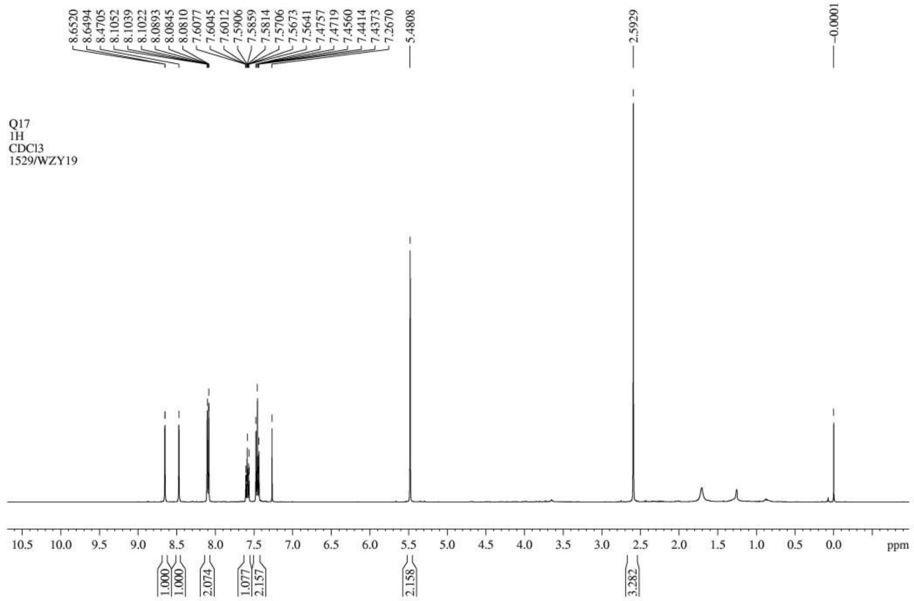

^{1}H NMR spectrum of compound **3b**

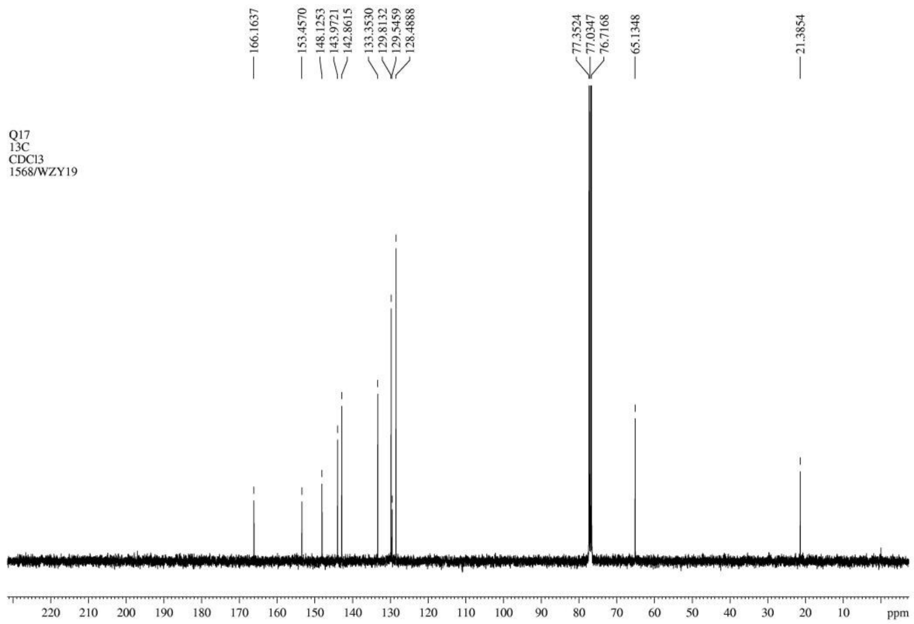

^{13}C NMR spectrum of compound **3b**

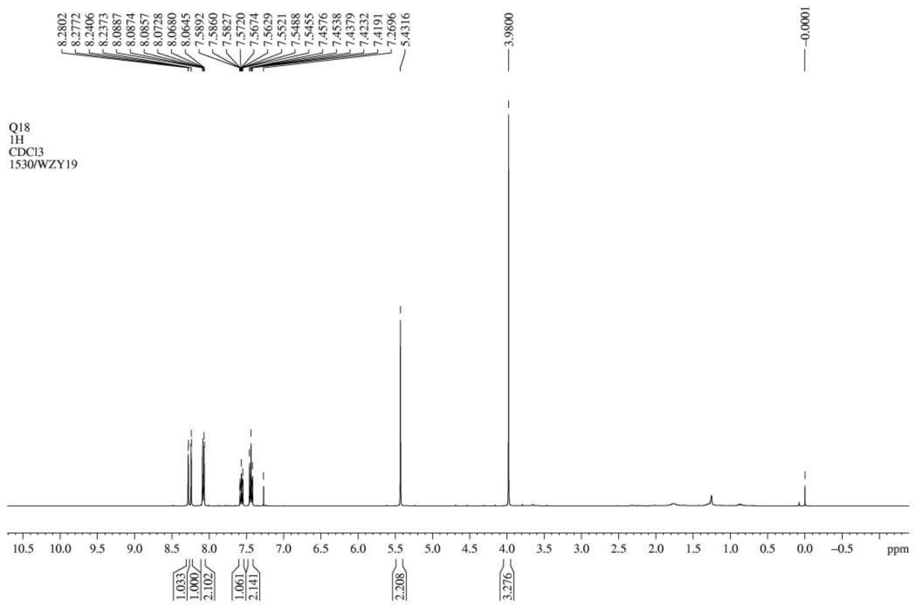

^{1}H NMR spectrum of compound **3c**

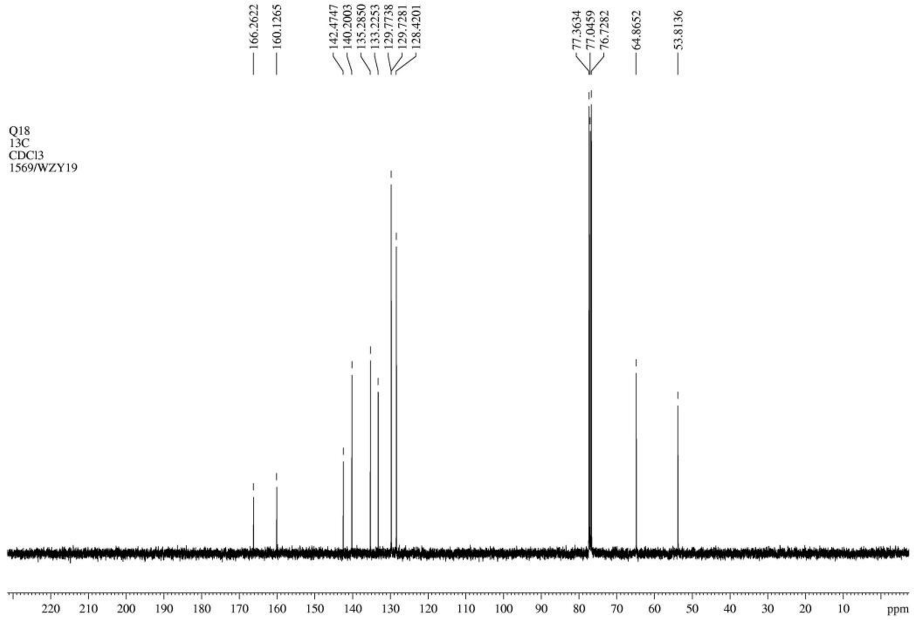

^{13}C NMR spectrum of compound **3c**

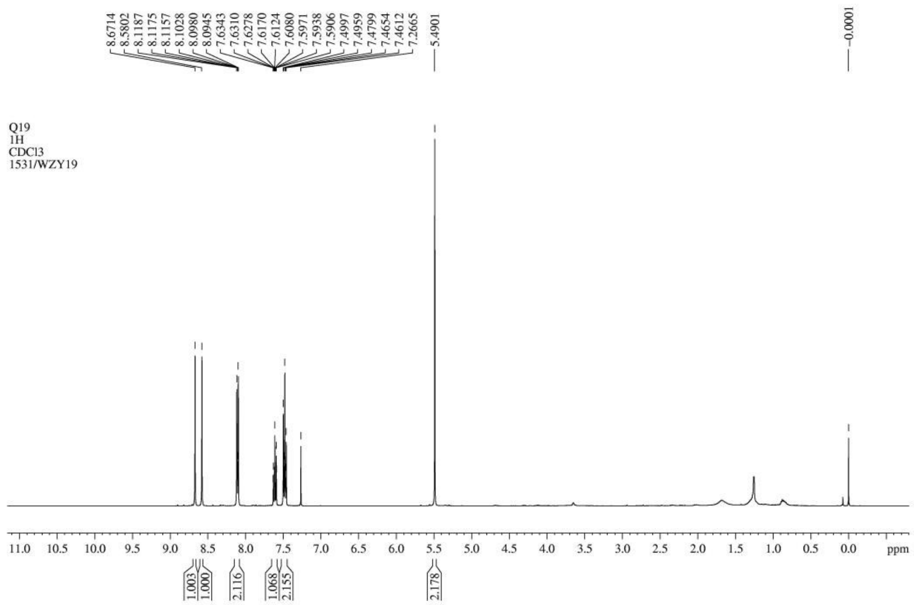

^{1}H NMR spectrum of compound **3d**

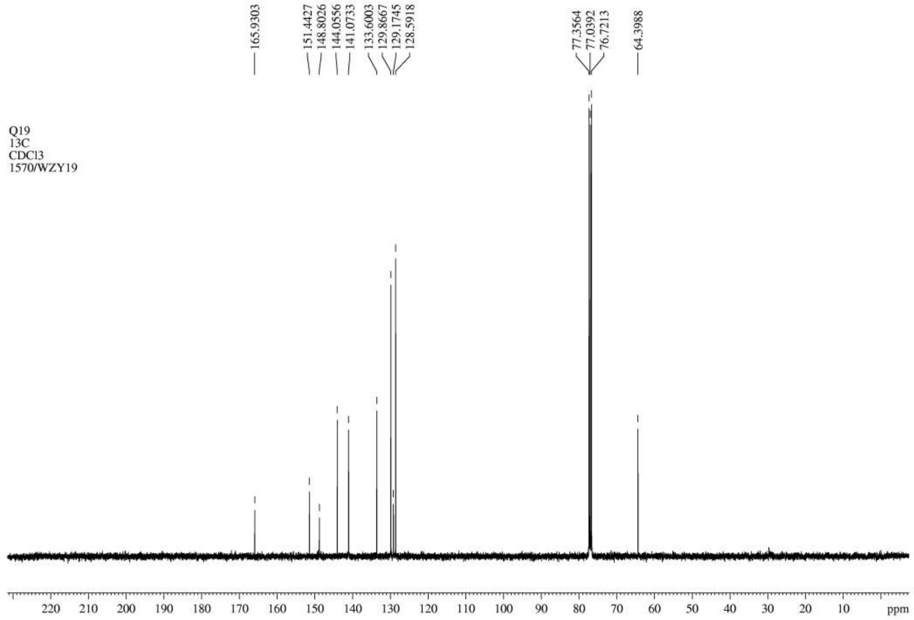

^{13}C NMR spectrum of compound **3d**

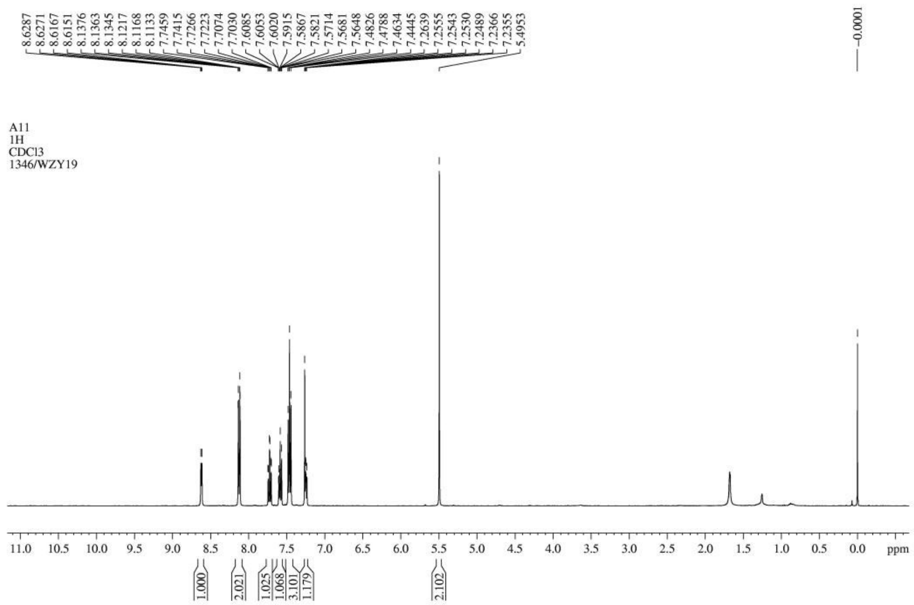

^{1}H NMR spectrum of compound **3e**

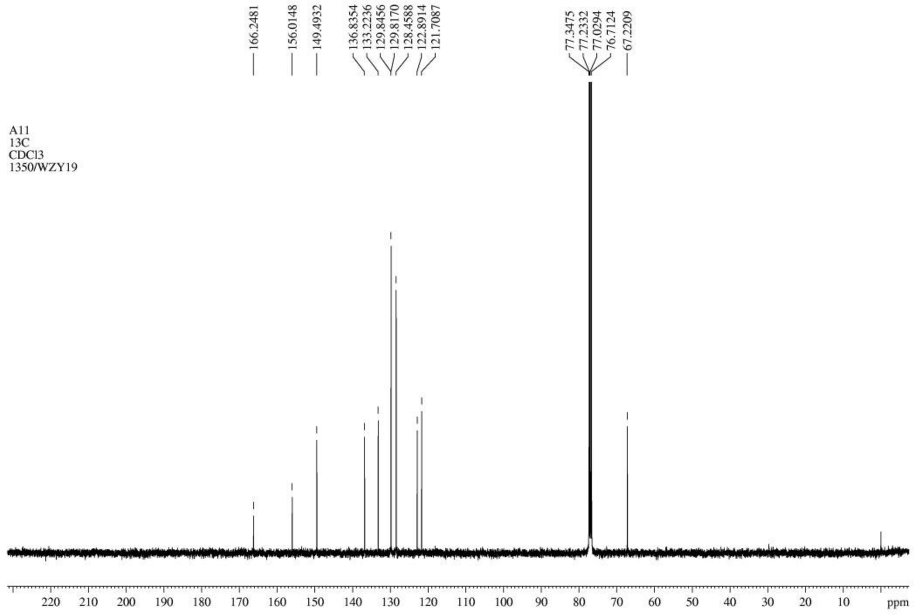

^{13}C NMR spectrum of compound **3e**

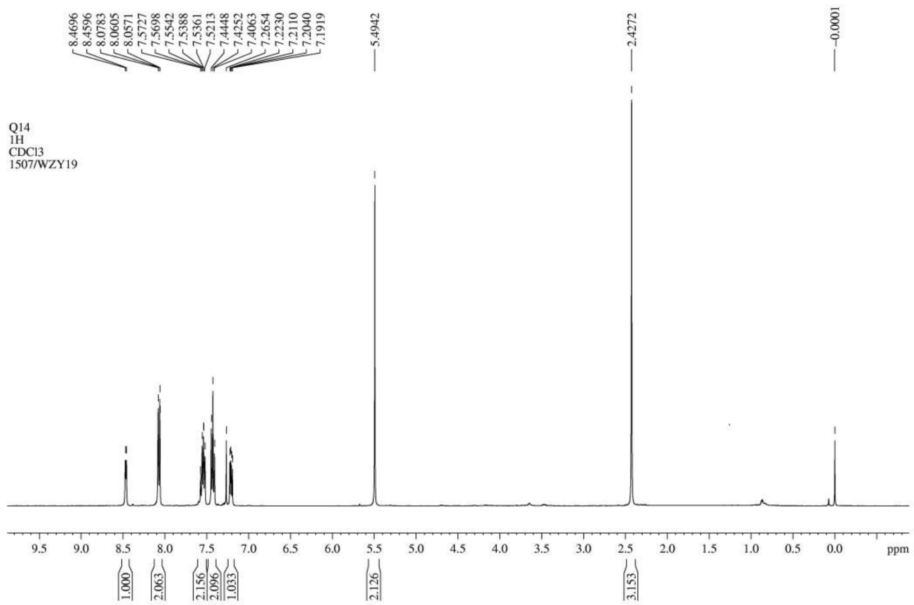

^{1}H NMR spectrum of compound **3f**

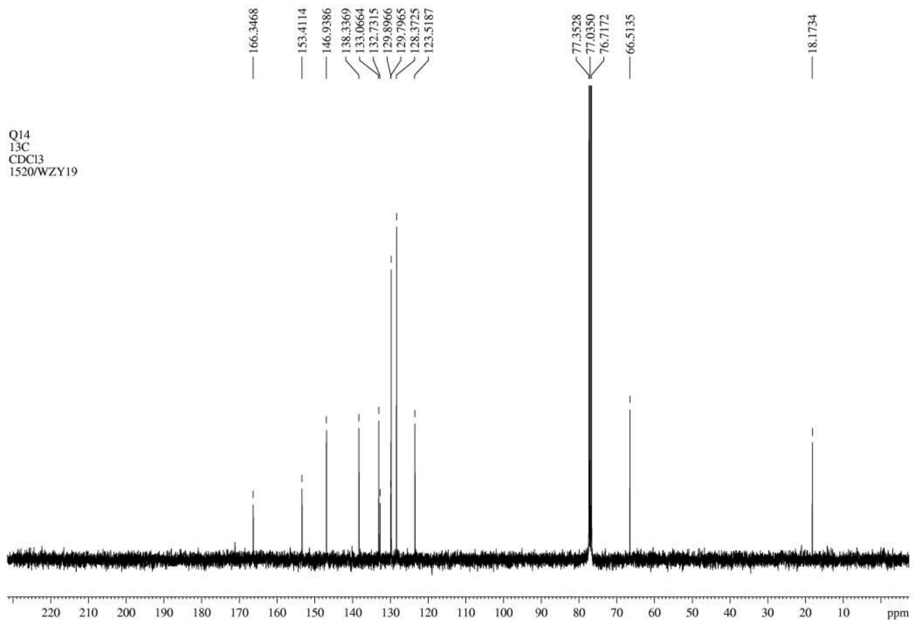

^{13}C NMR spectrum of compound **3f**

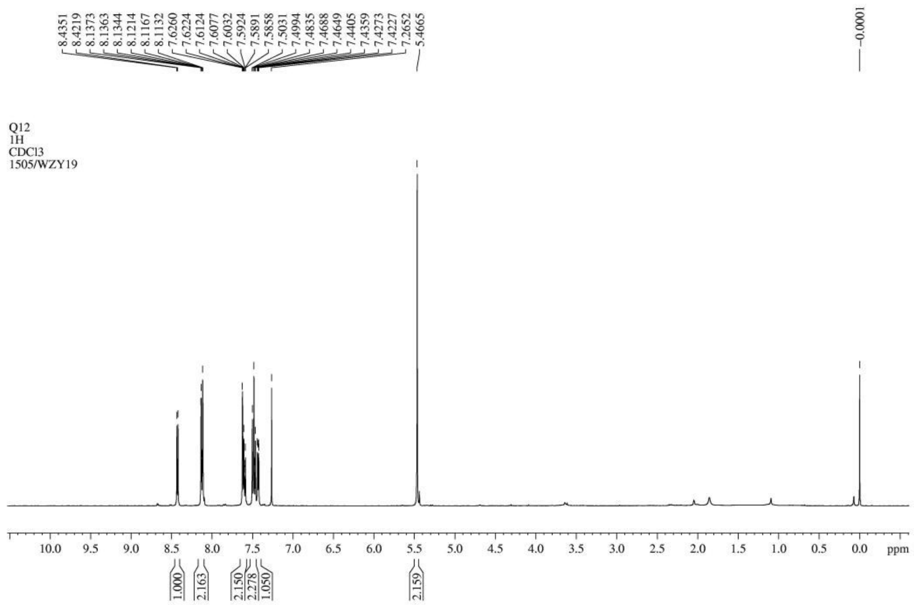

^{1}H NMR spectrum of compound **3g**

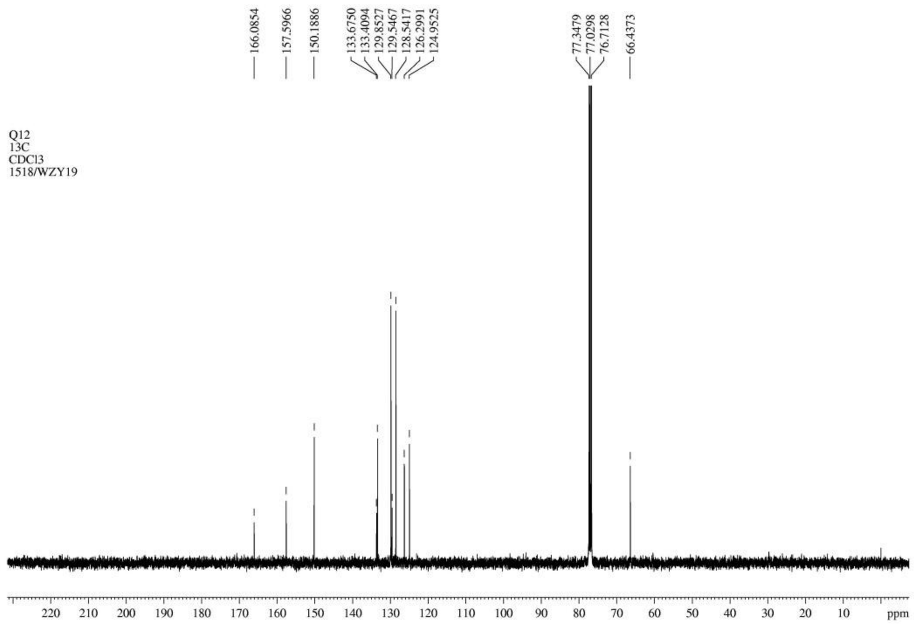

^{13}C NMR spectrum of compound **3g**

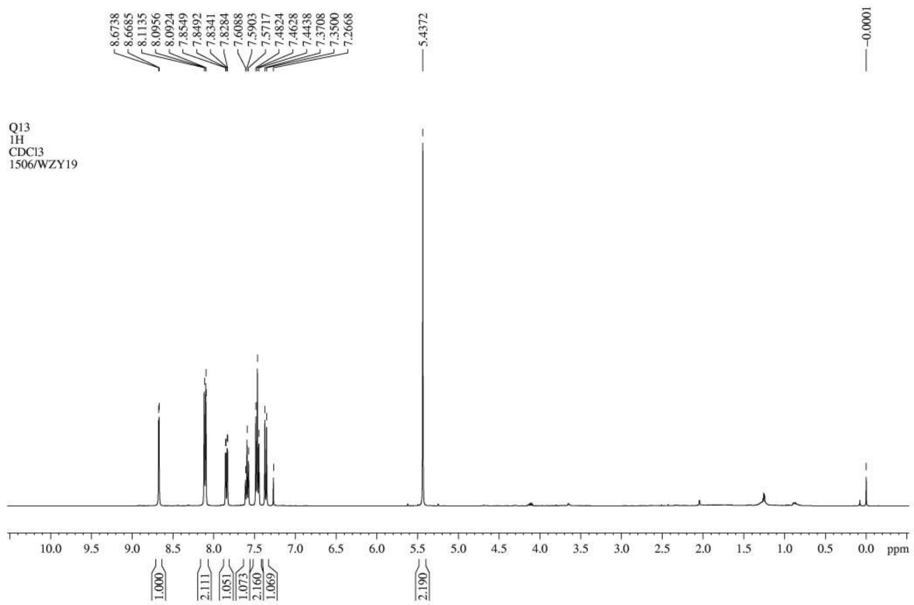

^{1}H NMR spectrum of compound **3h**

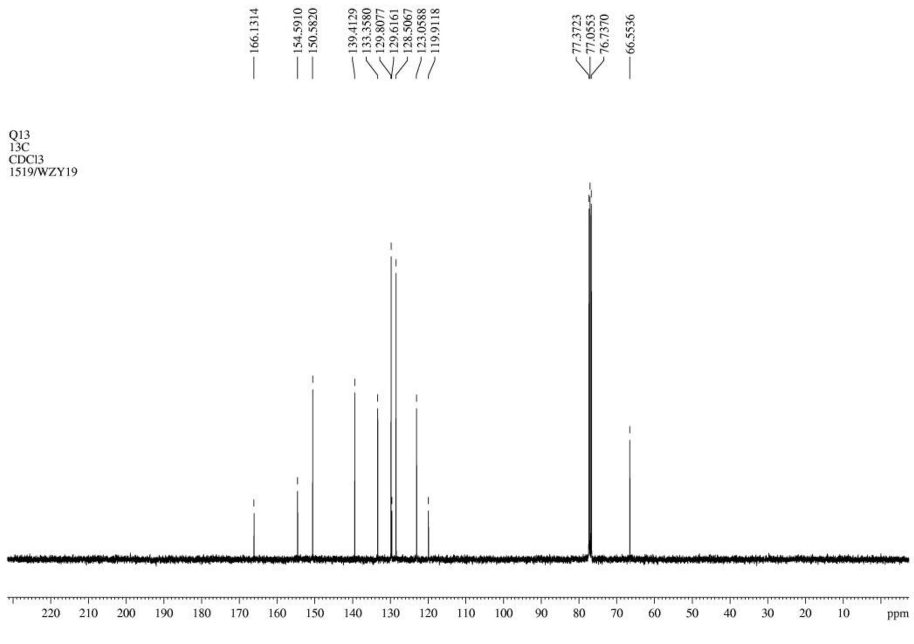

^{13}C NMR spectrum of compound **3h**

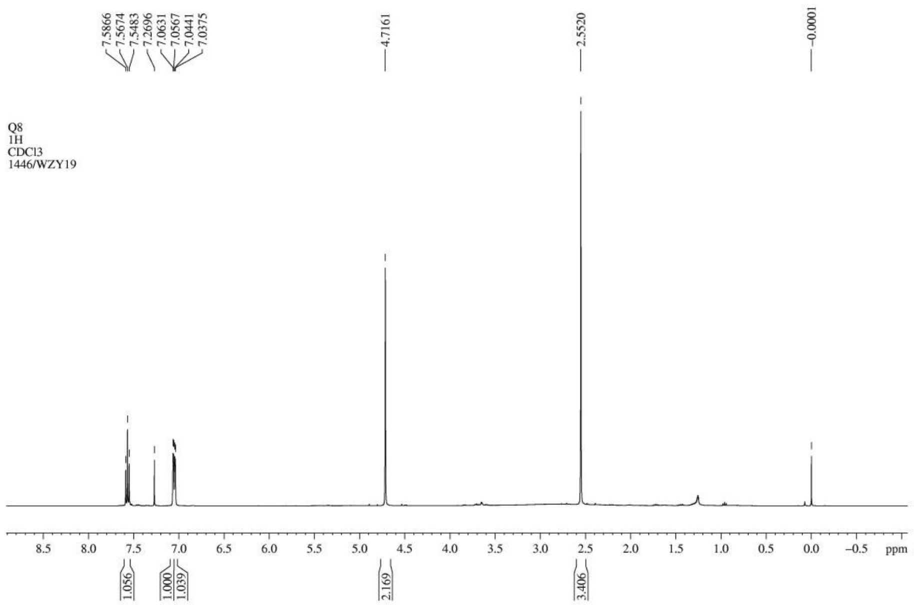

^{1}H NMR spectrum of compound **3i**

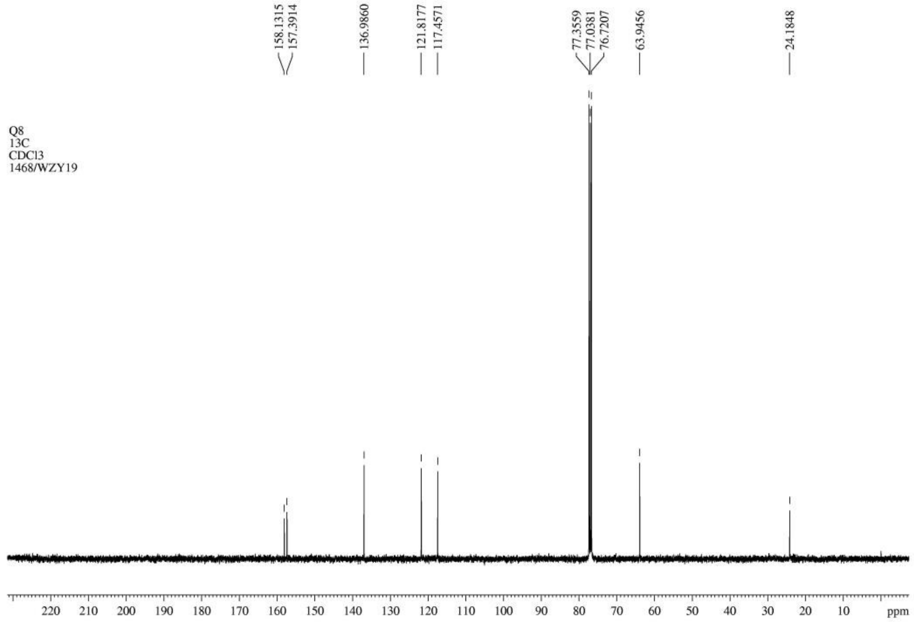

^{13}C NMR spectrum of compound **3i**

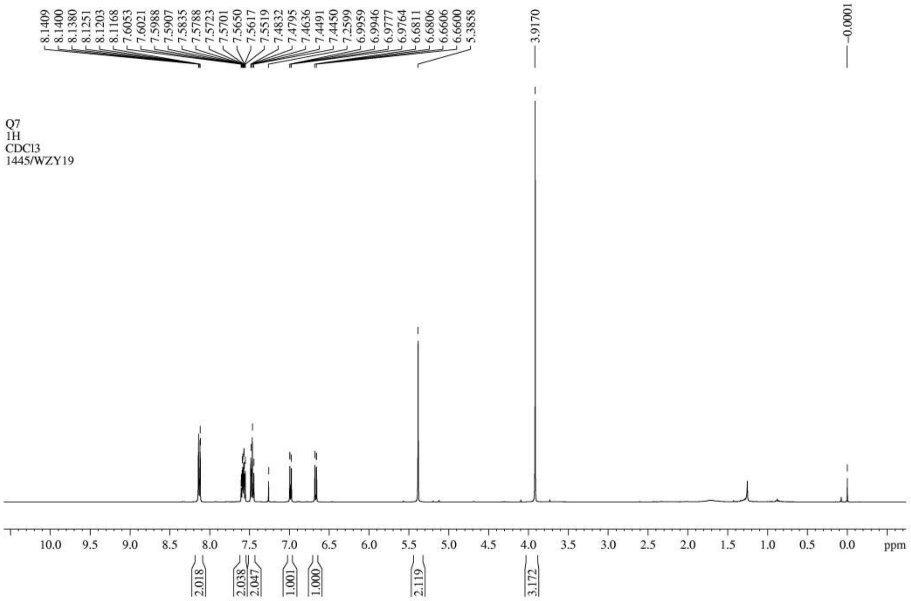

^{1}H NMR spectrum of compound **3j**

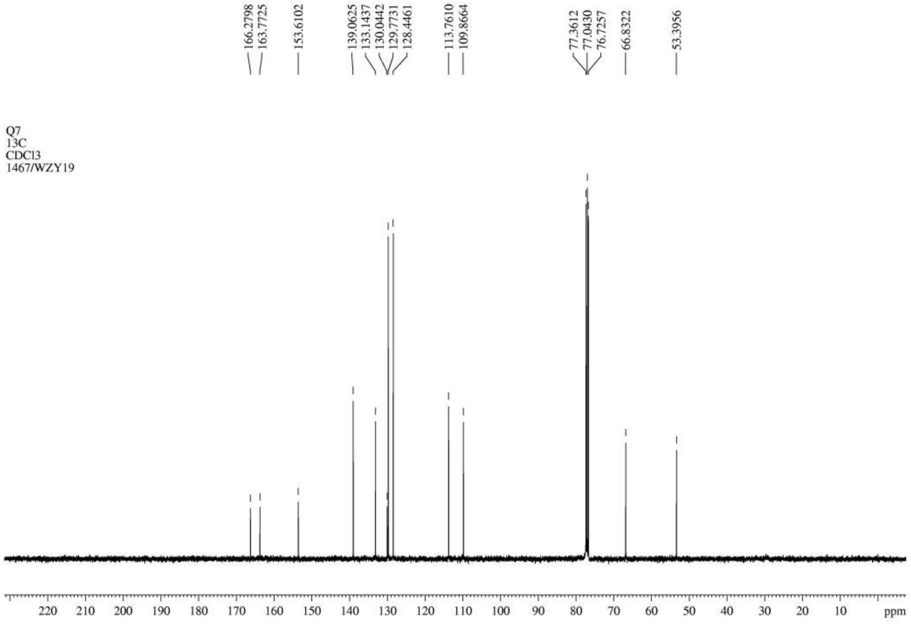

^{13}C NMR spectrum of compound **3j**

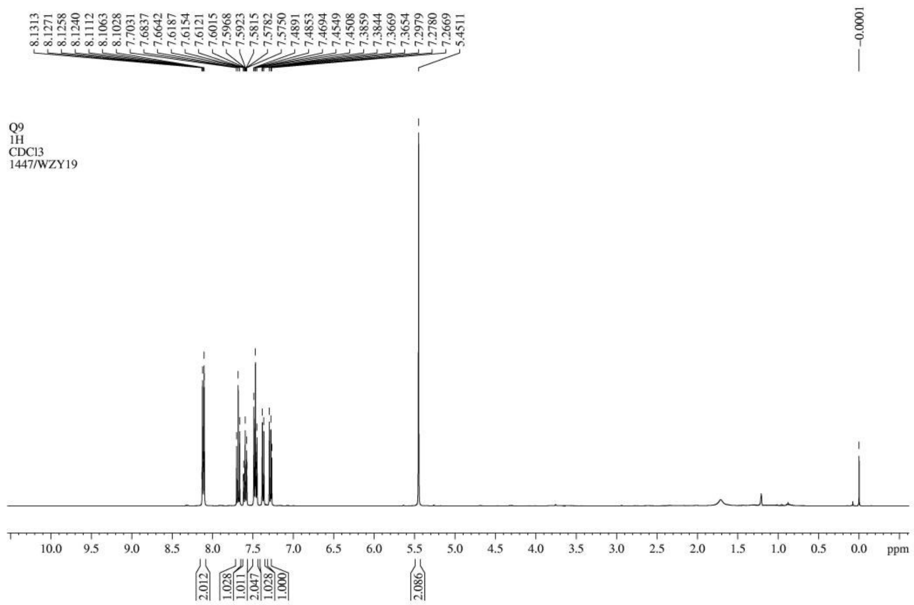

^{1}H NMR spectrum of compound **3k**

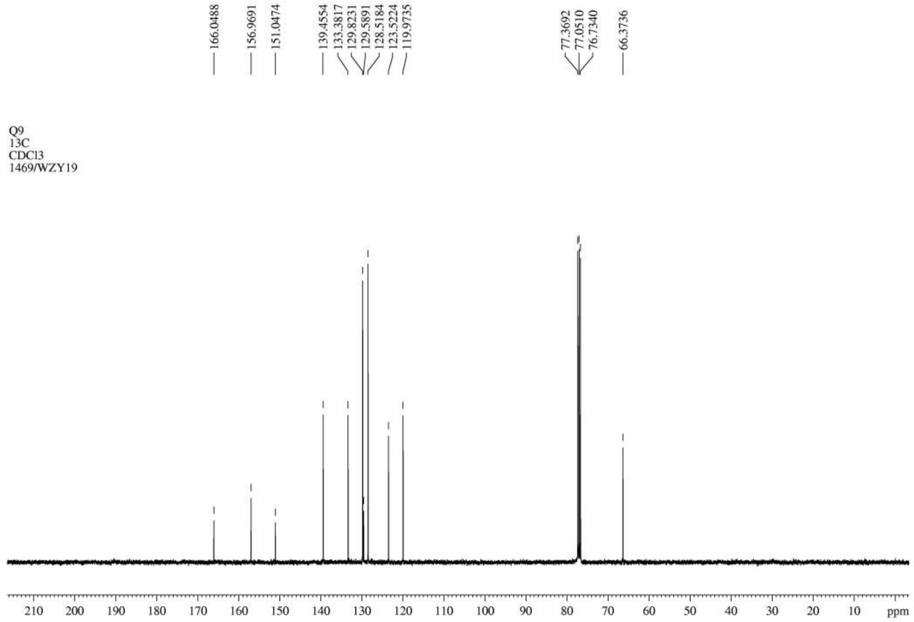

^{13}C NMR spectrum of compound **3k**

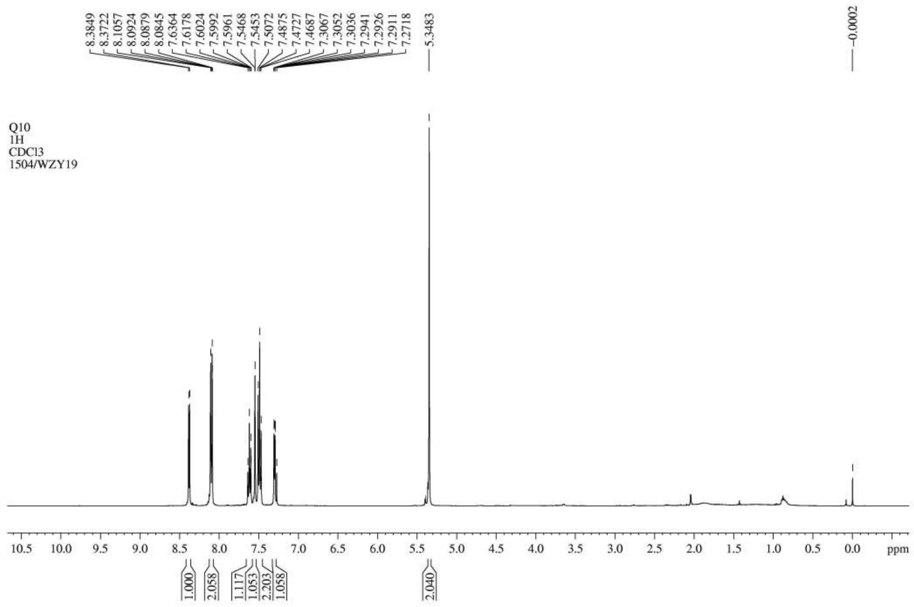

^{1}H NMR spectrum of compound **3l**

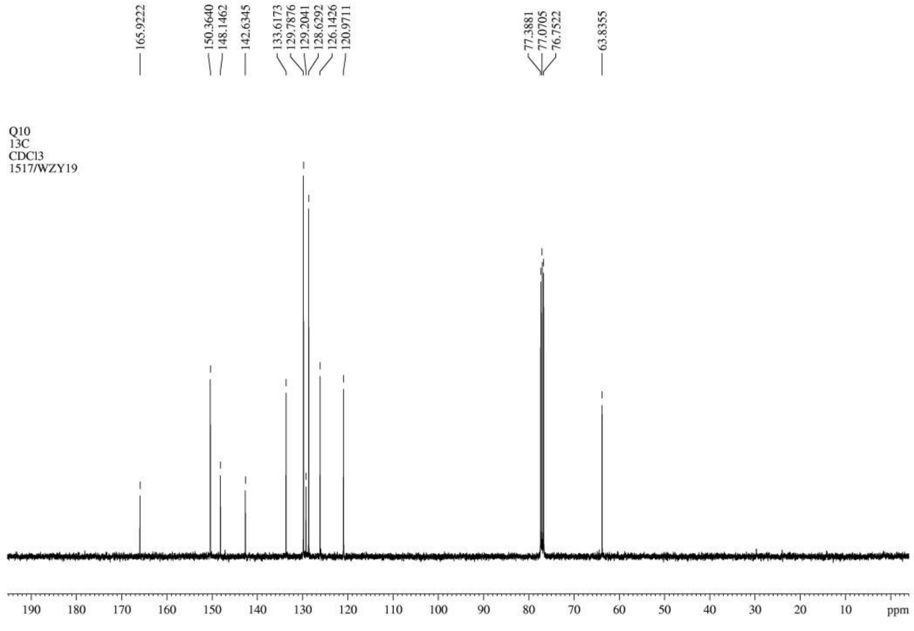

^{13}C NMR spectrum of compound **3l**

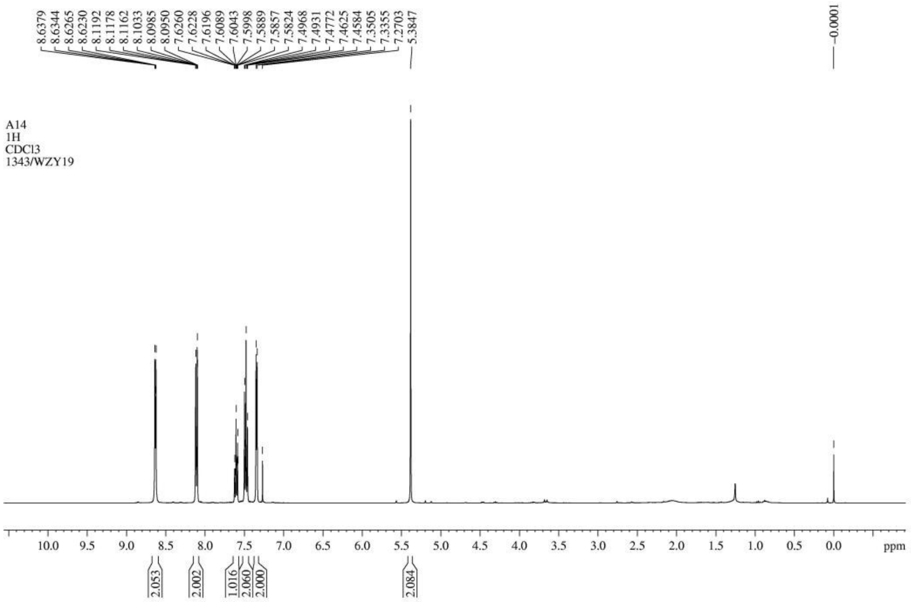

^{1}H NMR spectrum of compound **3n**

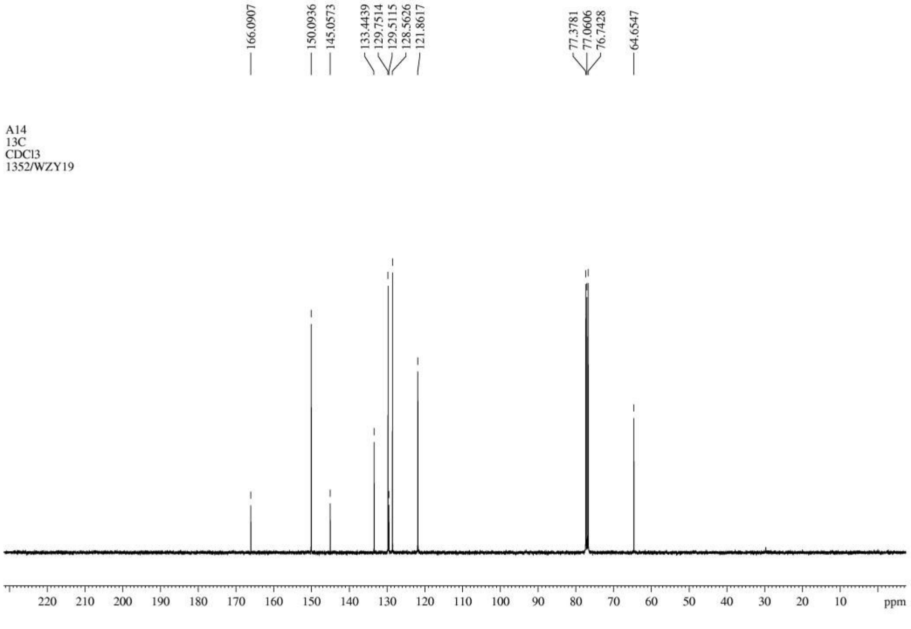

^{13}C NMR spectrum of compound **3n**

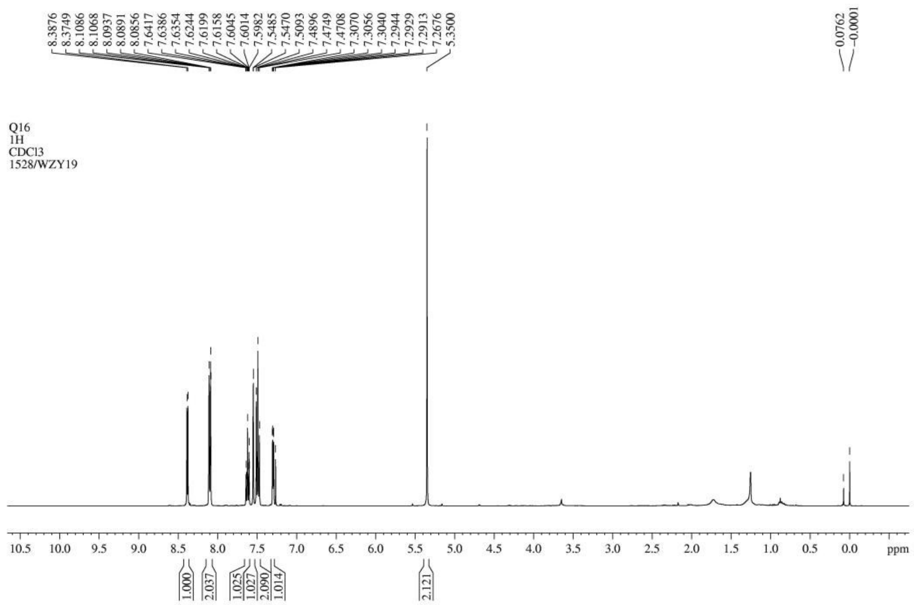

^{1}H NMR spectrum of compound **3o**

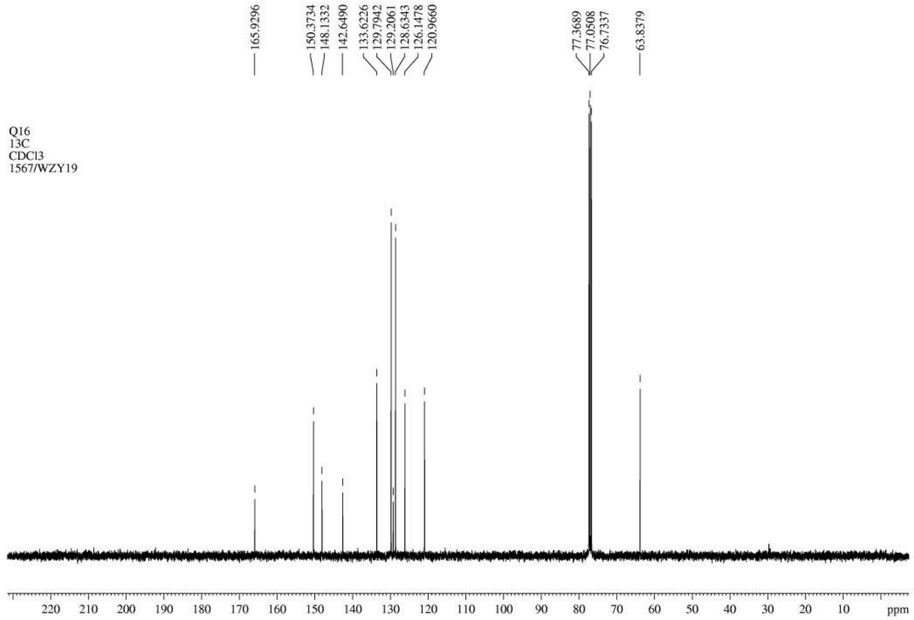

^{13}C NMR spectrum of compound **3o**

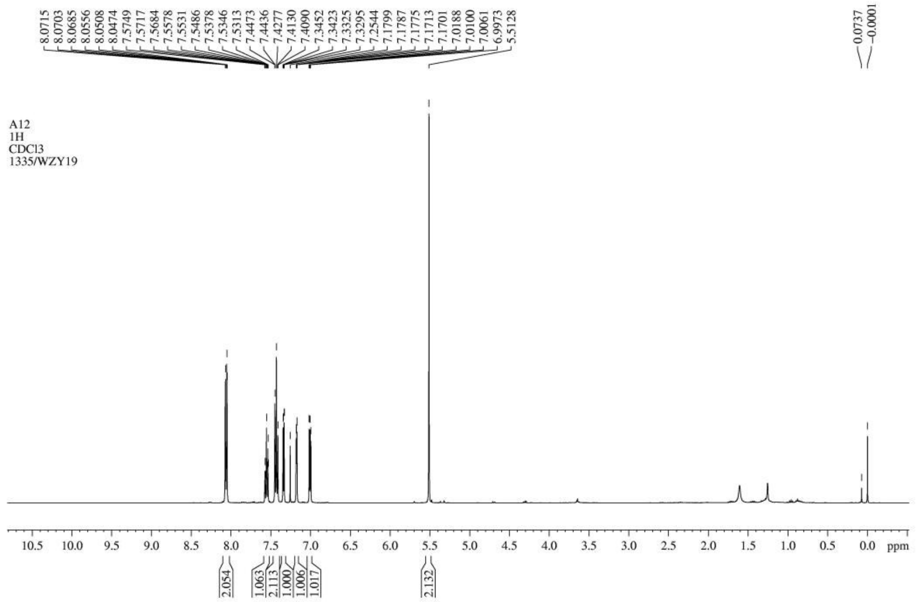

^{1}H NMR spectrum of compound **3p**

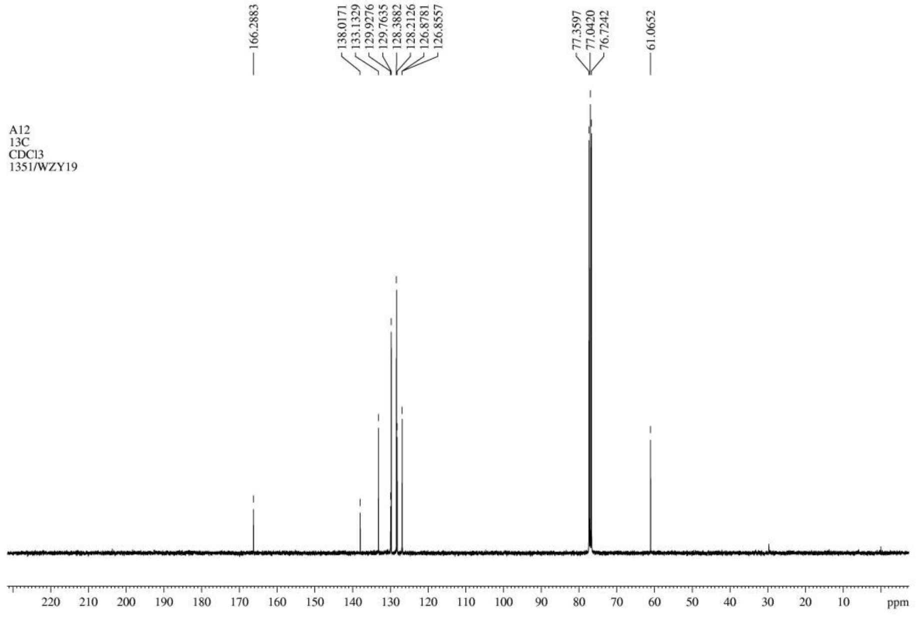

^{13}C NMR spectrum of compound **3p**

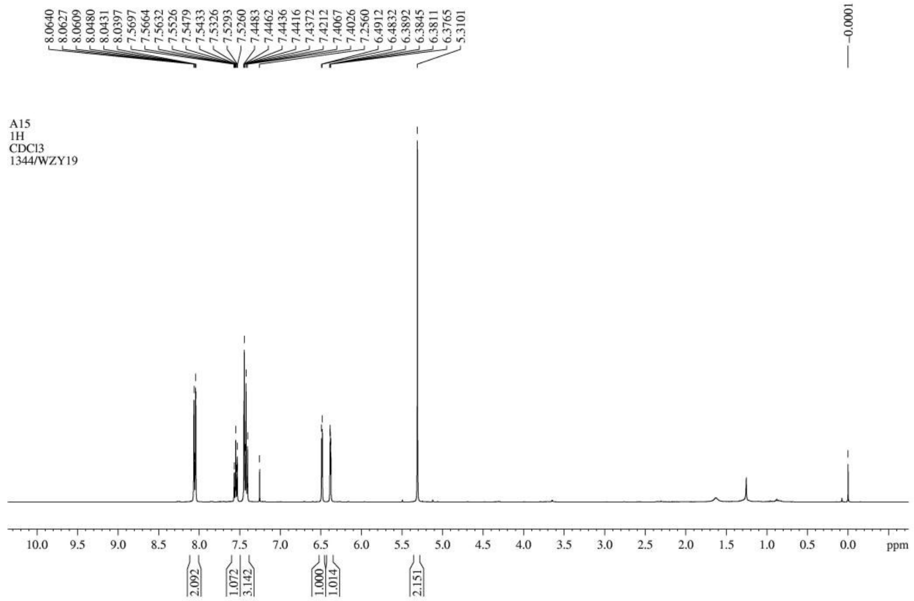

^{1}H NMR spectrum of compound **3q**

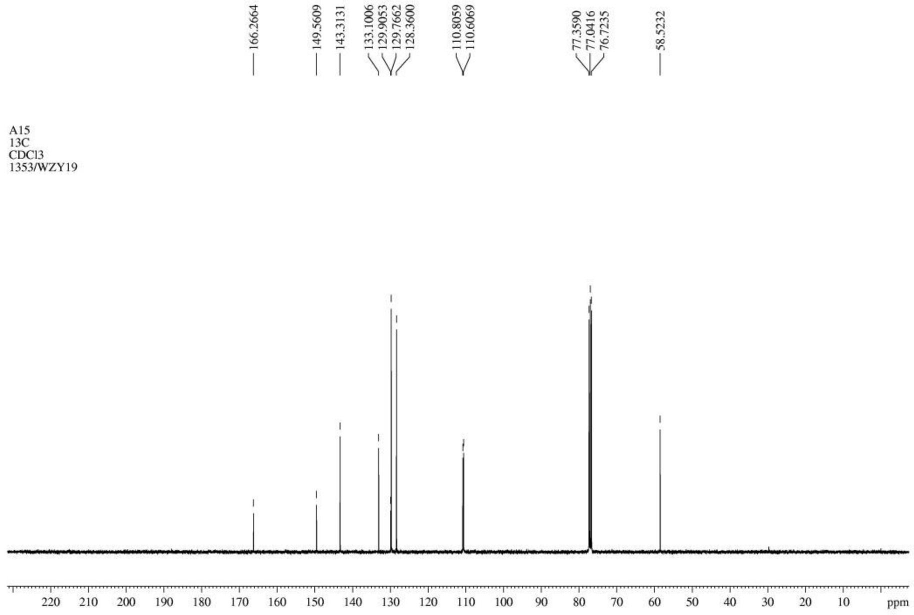

^{13}C NMR spectrum of compound **3q**

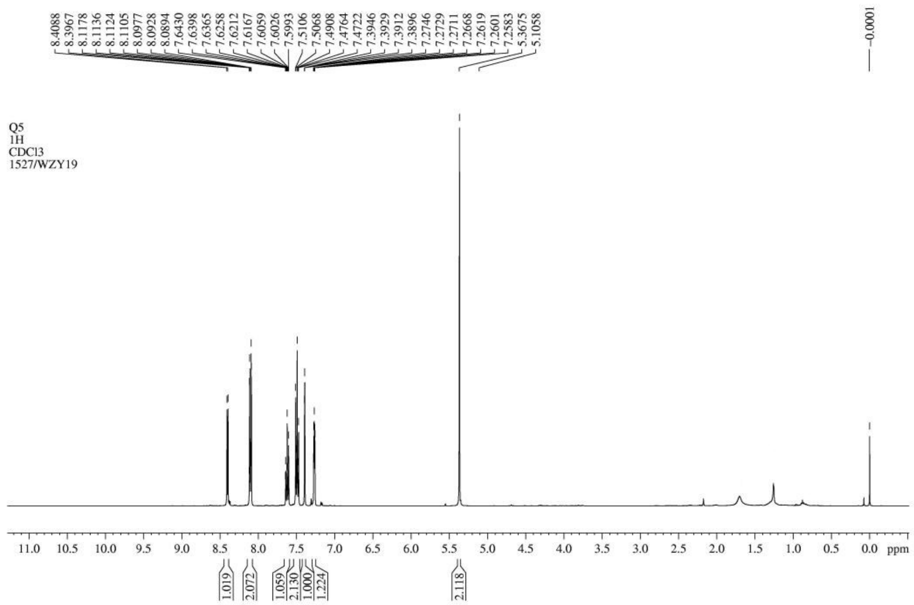

^{1}H NMR spectrum of compound **3r**

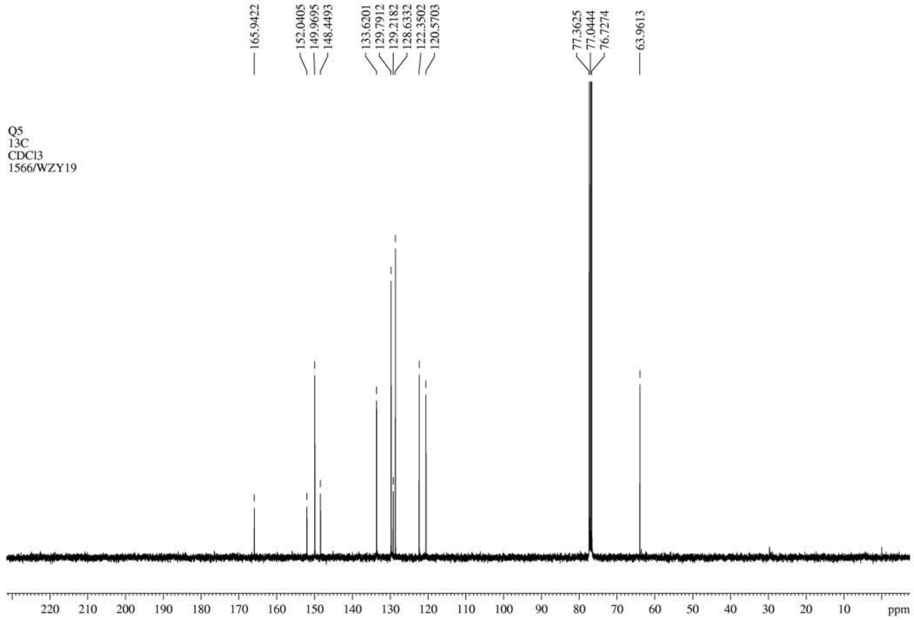

^{13}C NMR spectrum of compound **3r**

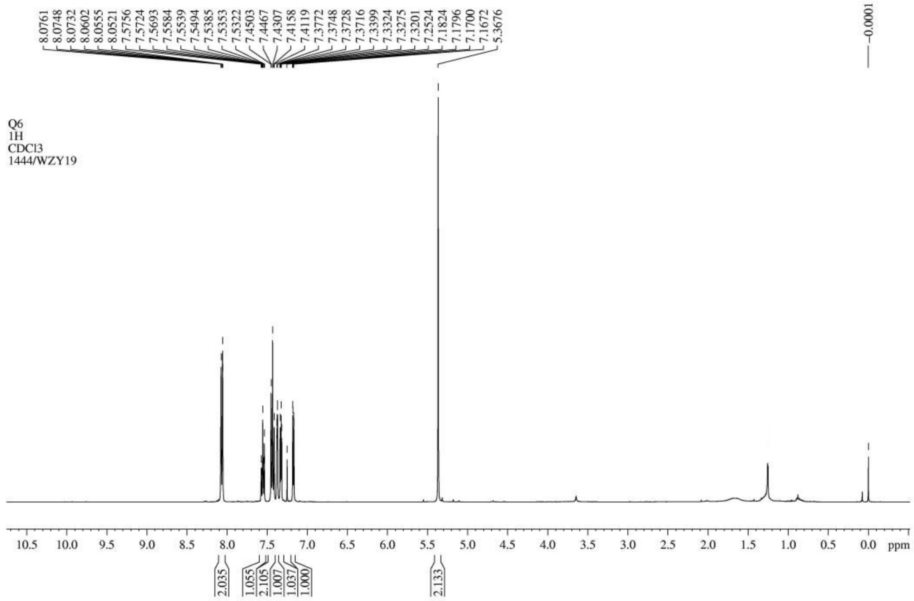

^{1}H NMR spectrum of compound **3s**

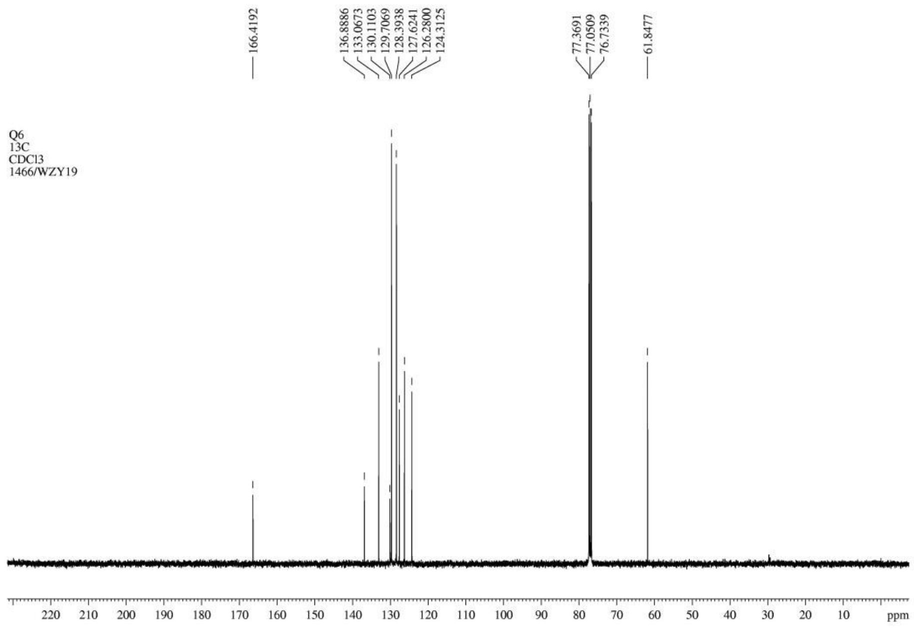

^{13}C NMR spectrum of compound **3s**

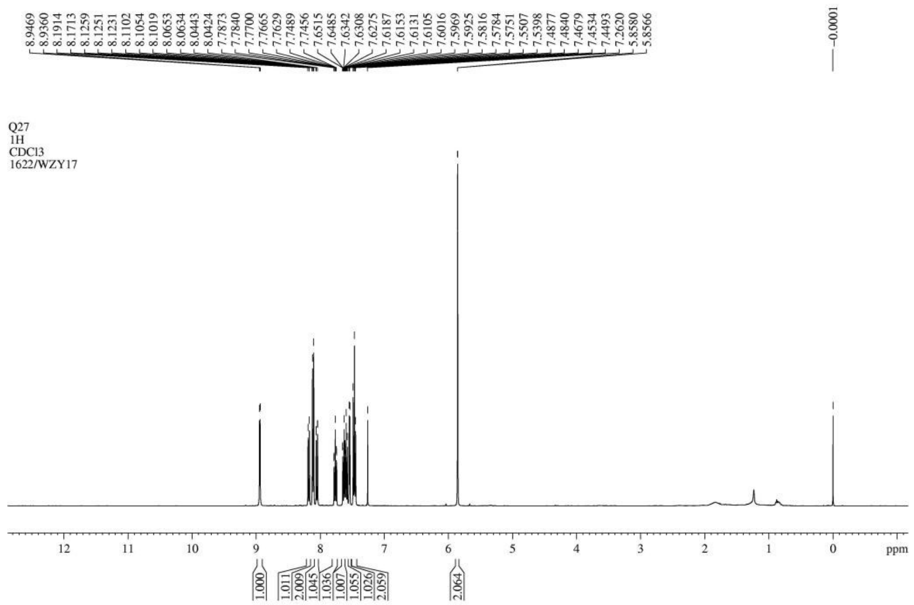

^{1}H NMR spectrum of compound **3t**

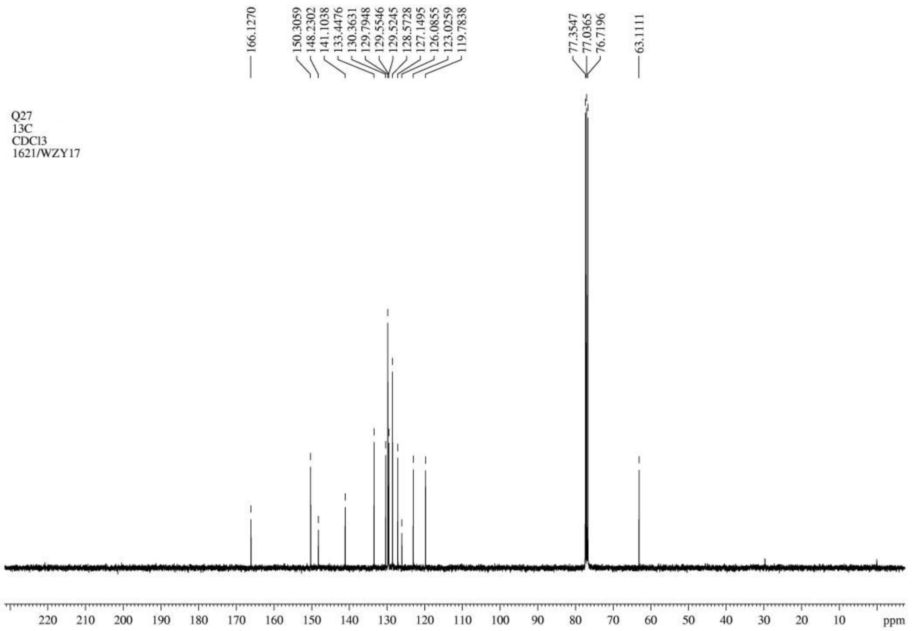

^{13}C NMR spectrum of compound **3t**

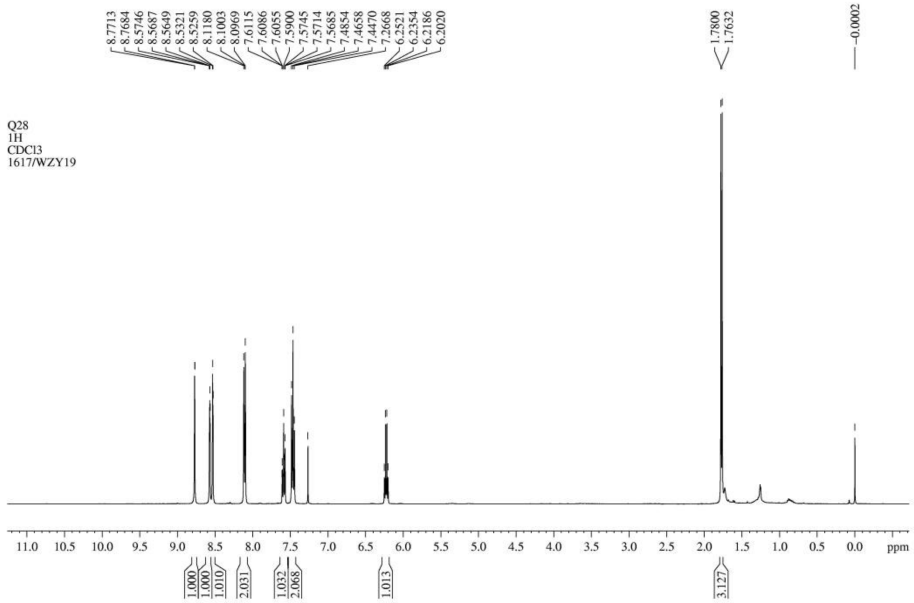

^{1}H NMR spectrum of compound **3u**

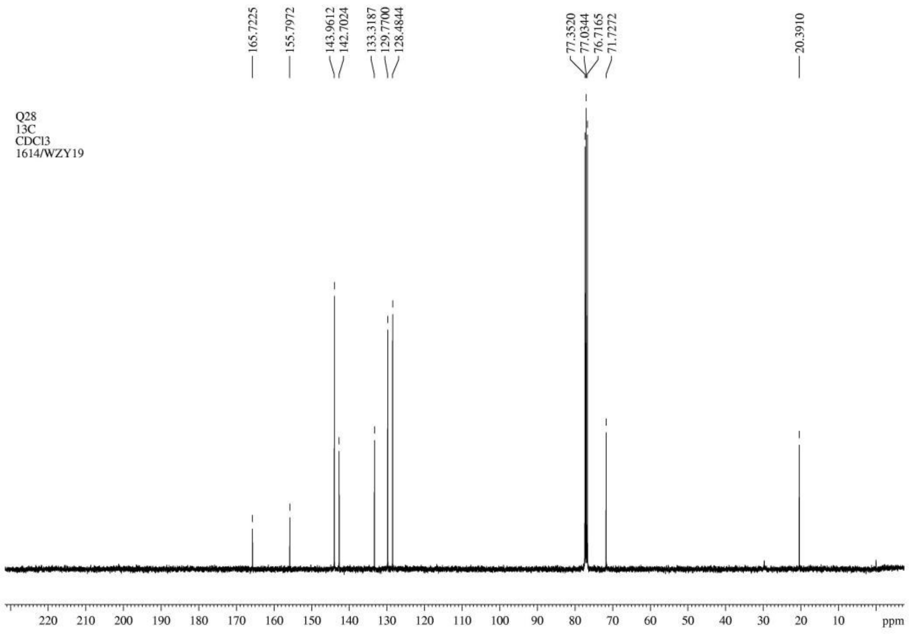

^{13}C NMR spectrum of compound **3u**

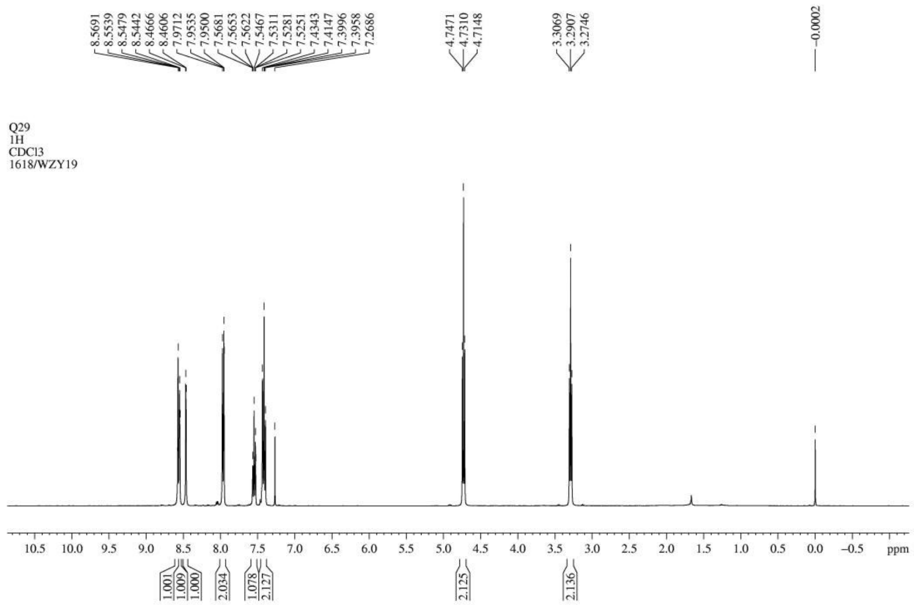

^{1}H NMR spectrum of compound **3v**

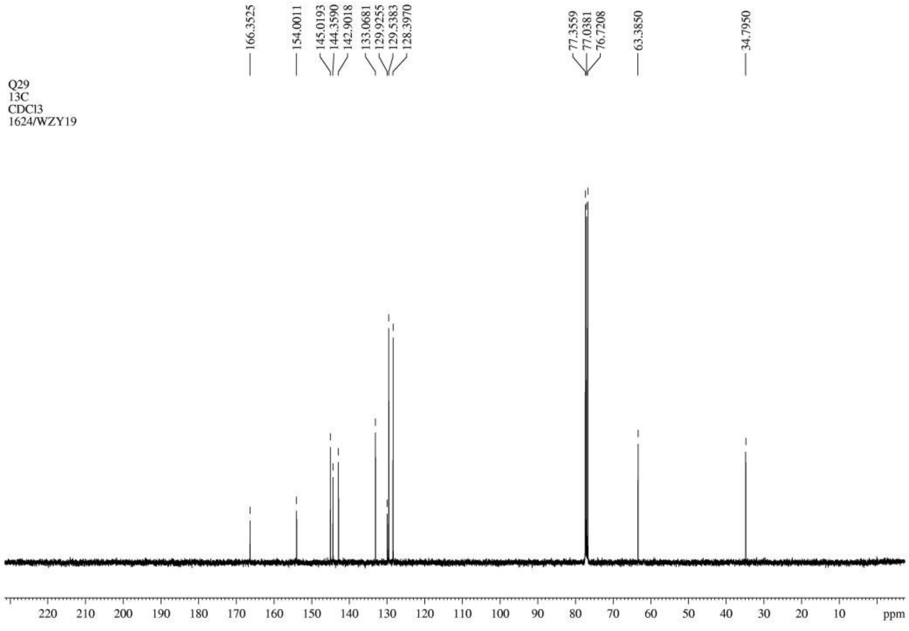

^{13}C NMR spectrum of compound **3v**

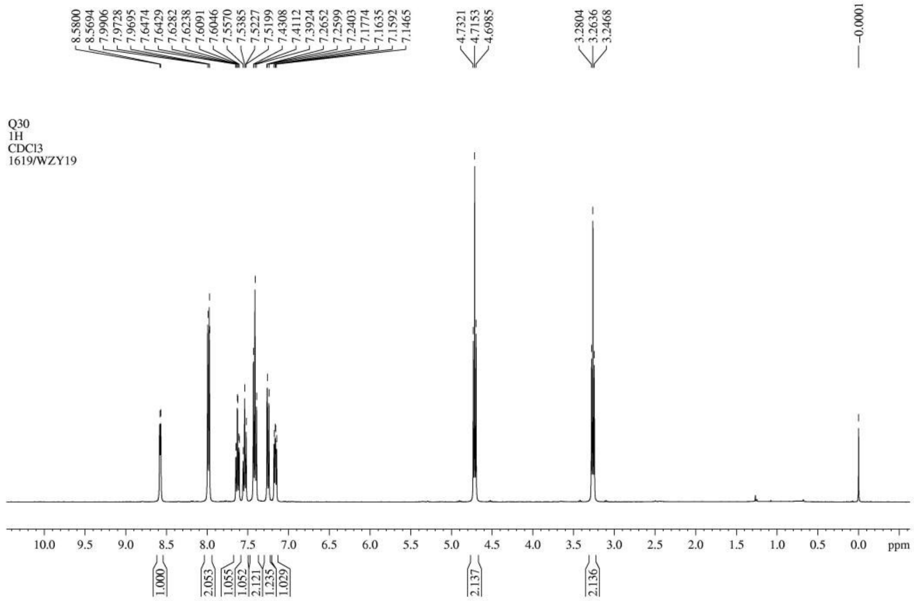

^{1}H NMR spectrum of compound **3w**

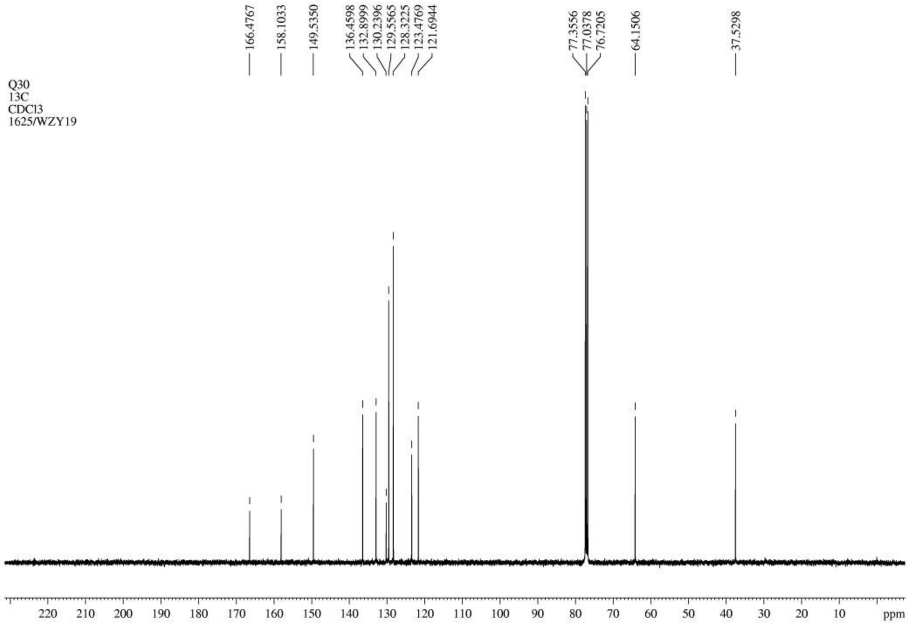

^{13}C NMR spectrum of compound **3w**

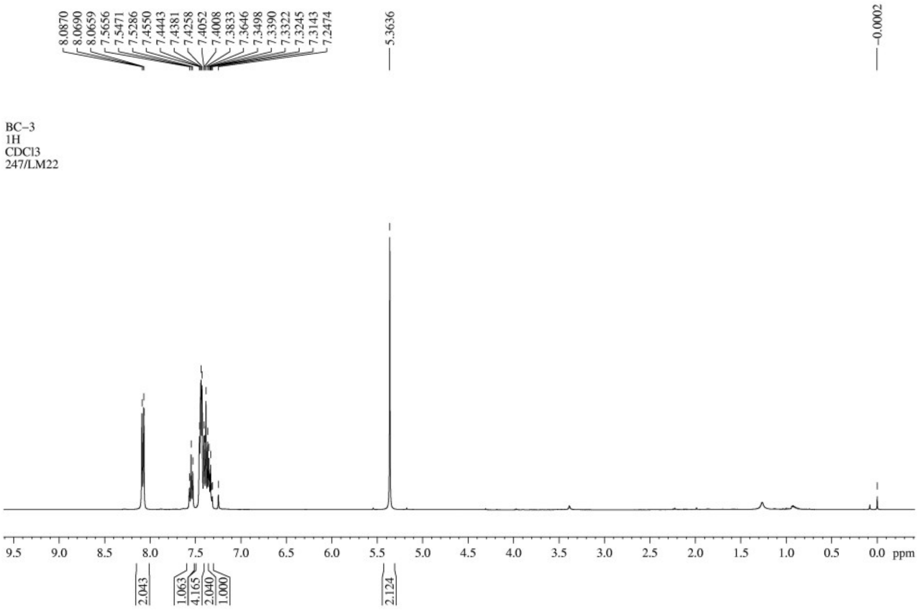

^{1}H NMR spectrum of compound **3x**

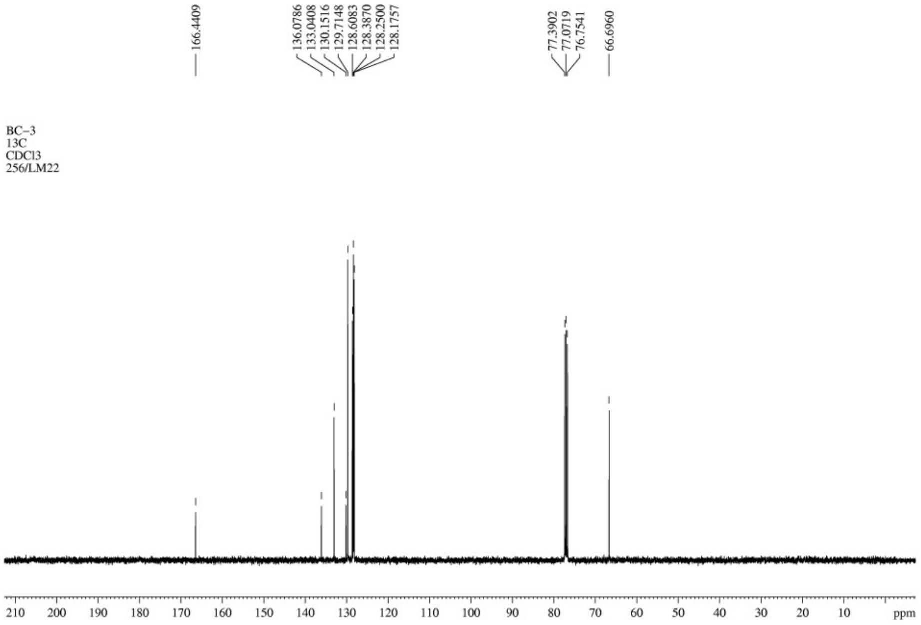

^{13}C NMR spectrum of compound **3x**

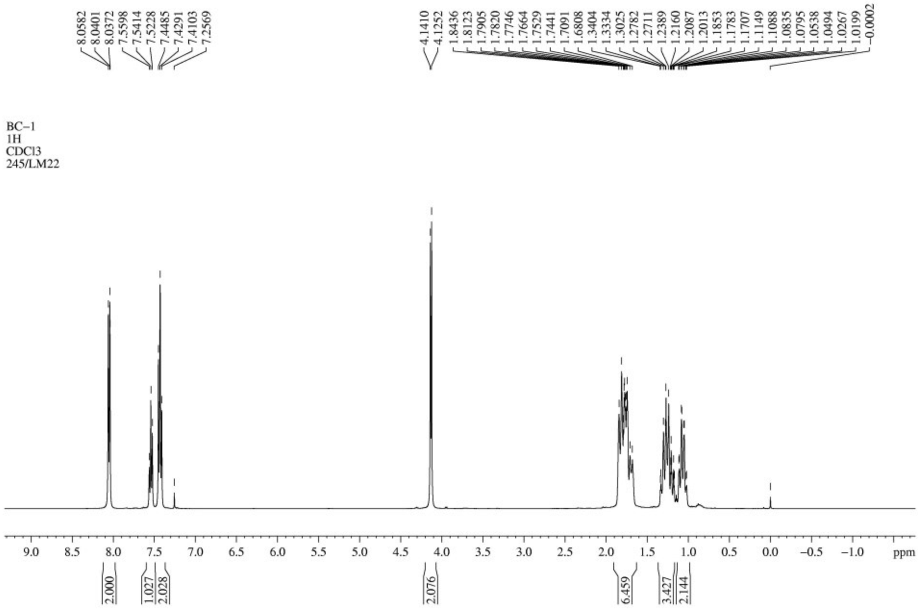

^{1}H NMR spectrum of compound **3y**

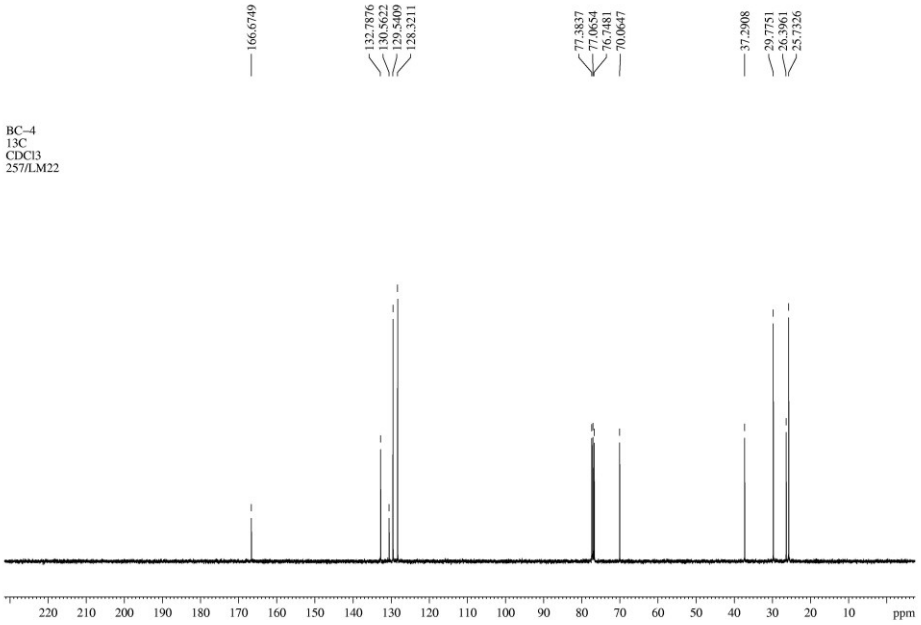

^{13}C NMR spectrum of compound **3y**

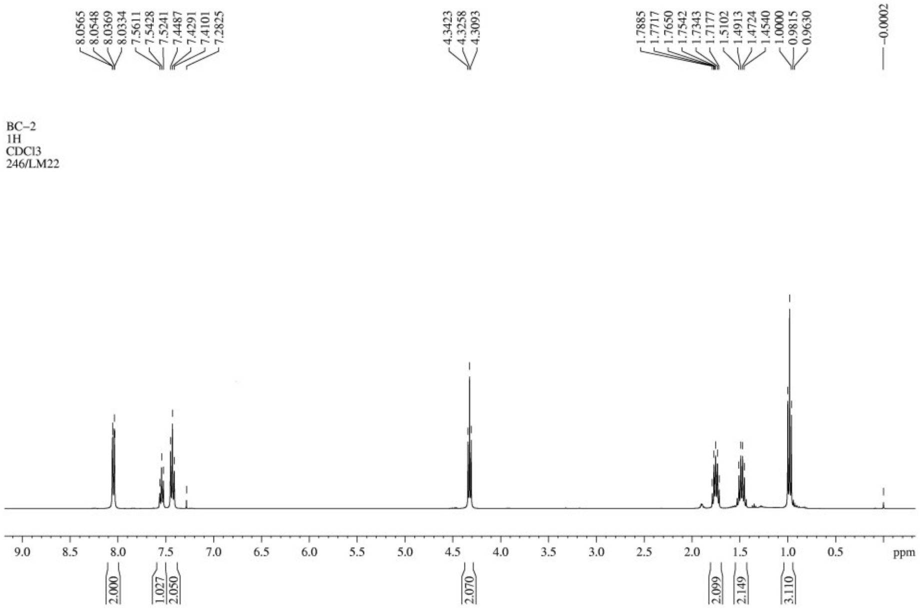

^{1}H NMR spectrum of compound **3z**

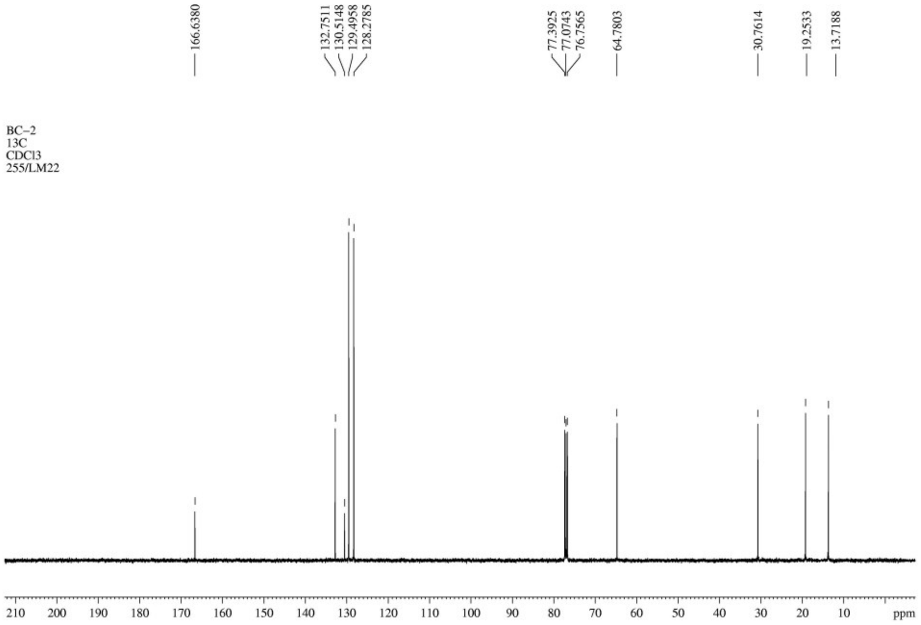

^{13}C NMR spectrum of compound **3z**

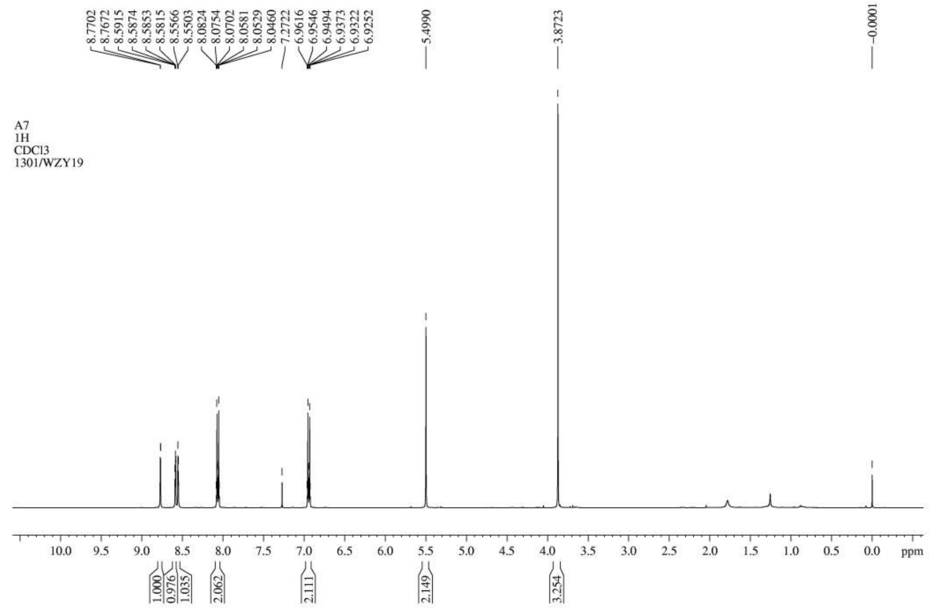

^1H NMR spectrum of compound **4a**

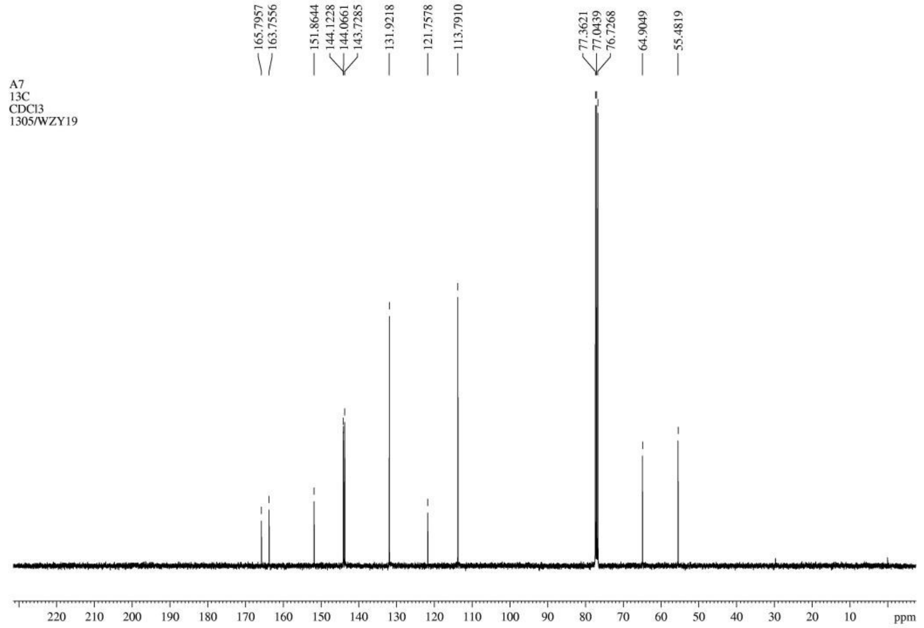

^{13}C NMR spectrum of compound **4a**

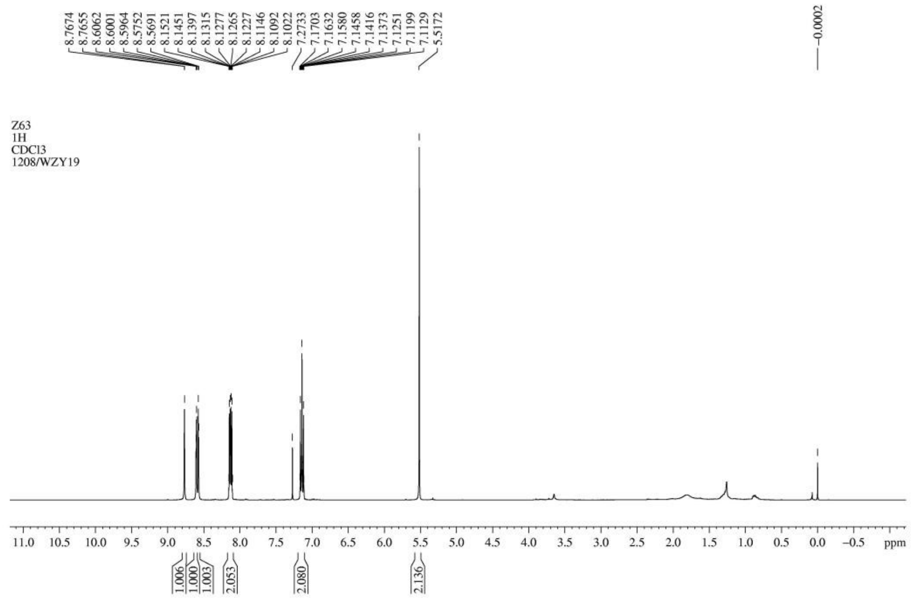

^{1}H NMR spectrum of compound **4b**

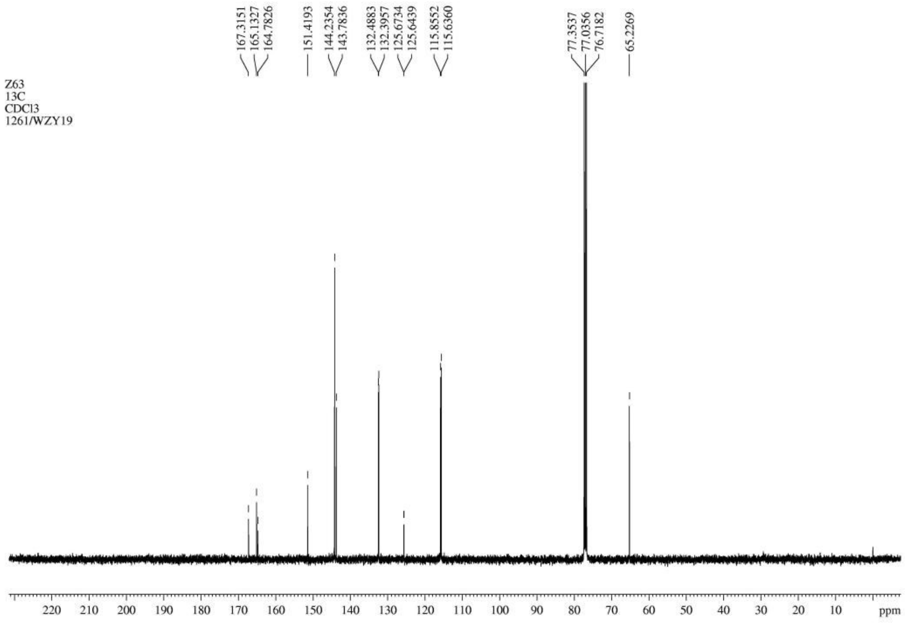

^{13}C NMR spectrum of compound **4b**

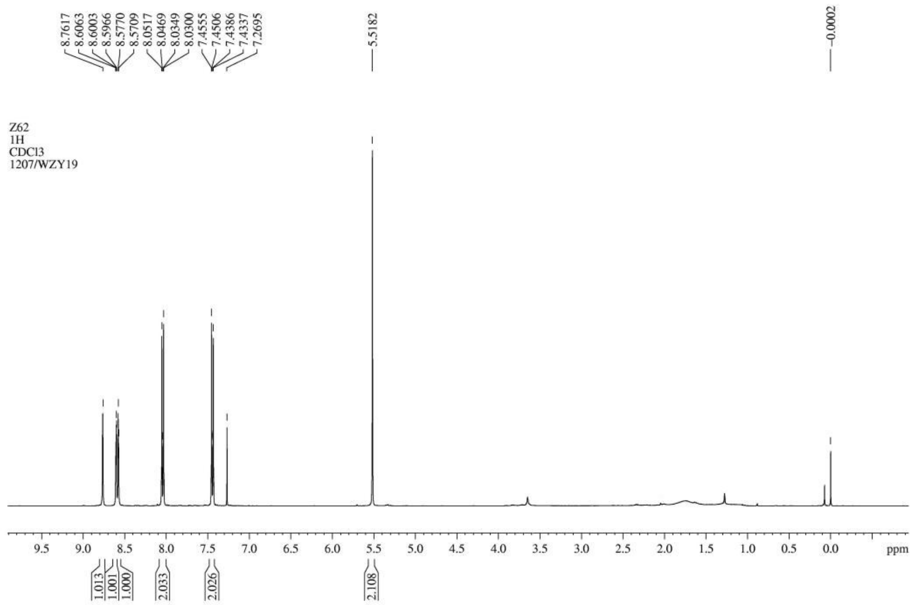

^{1}H NMR spectrum of compound **4c**

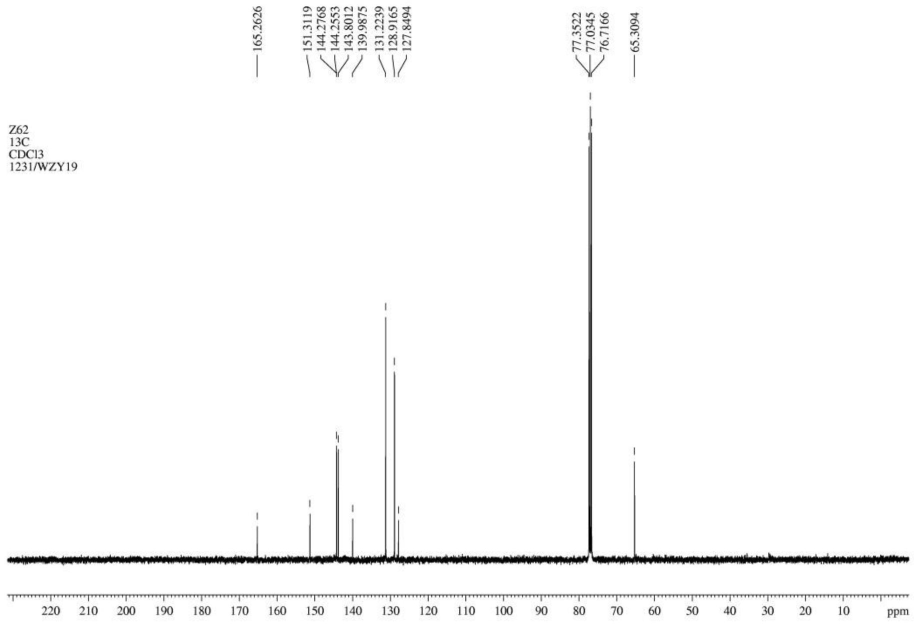

^{13}C NMR spectrum of compound **4c**

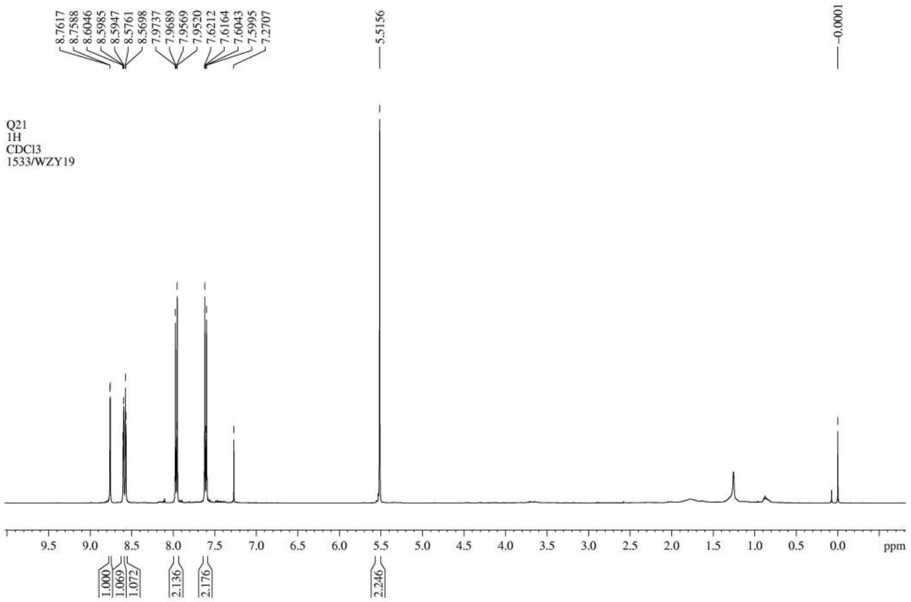

^{1}H NMR spectrum of compound **4d**

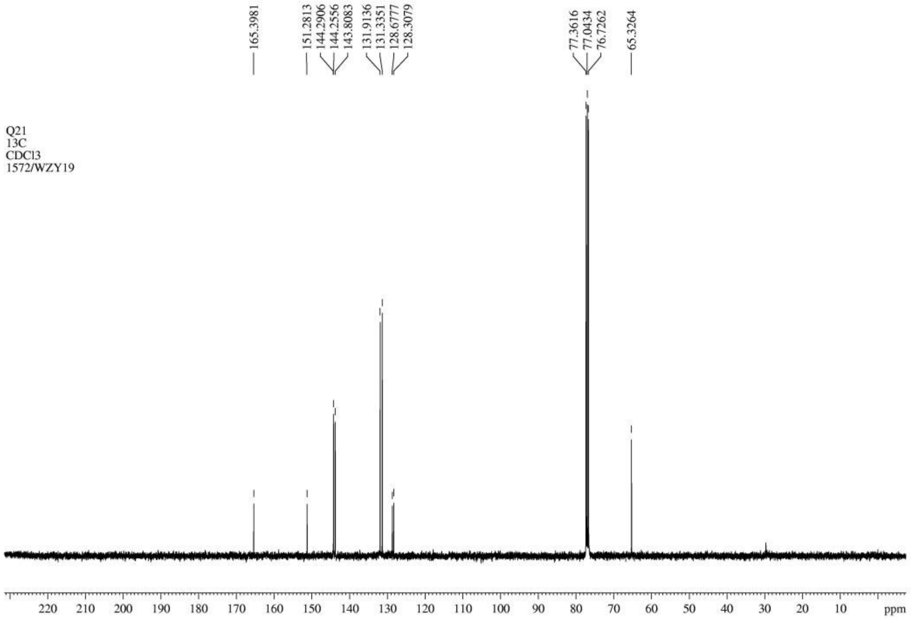

^{13}C NMR spectrum of compound **4d**

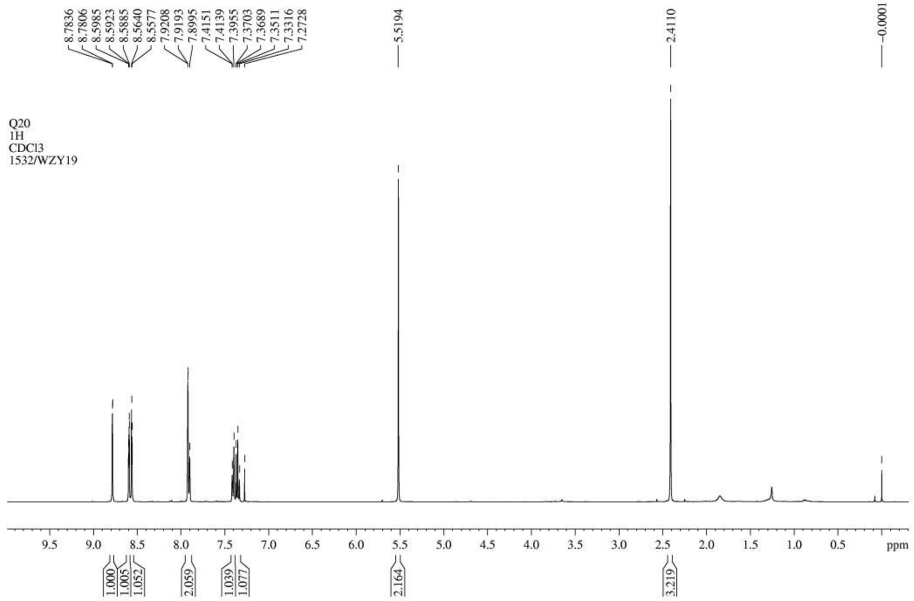

^{1}H NMR spectrum of compound **4e**

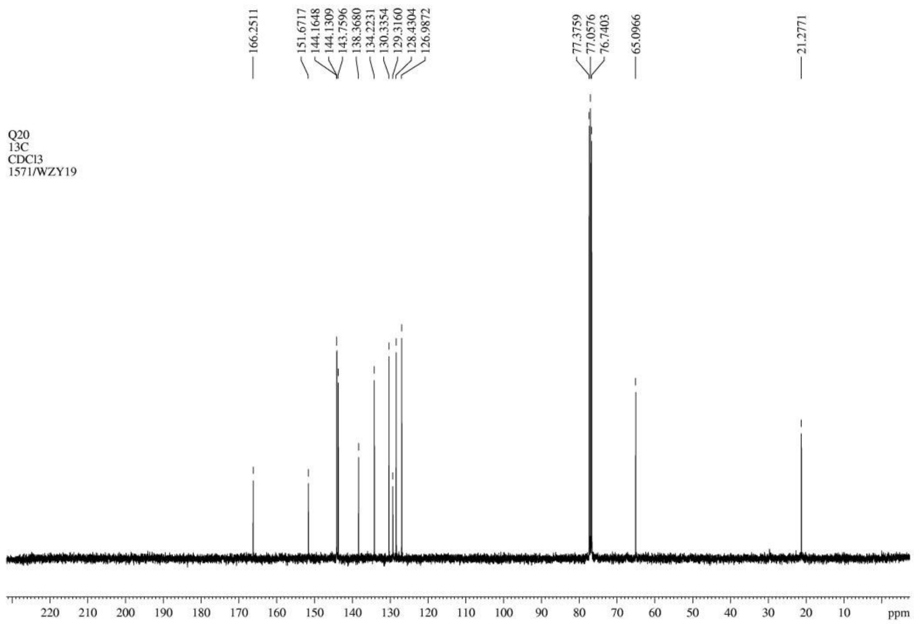

^{13}C NMR spectrum of compound **4e**

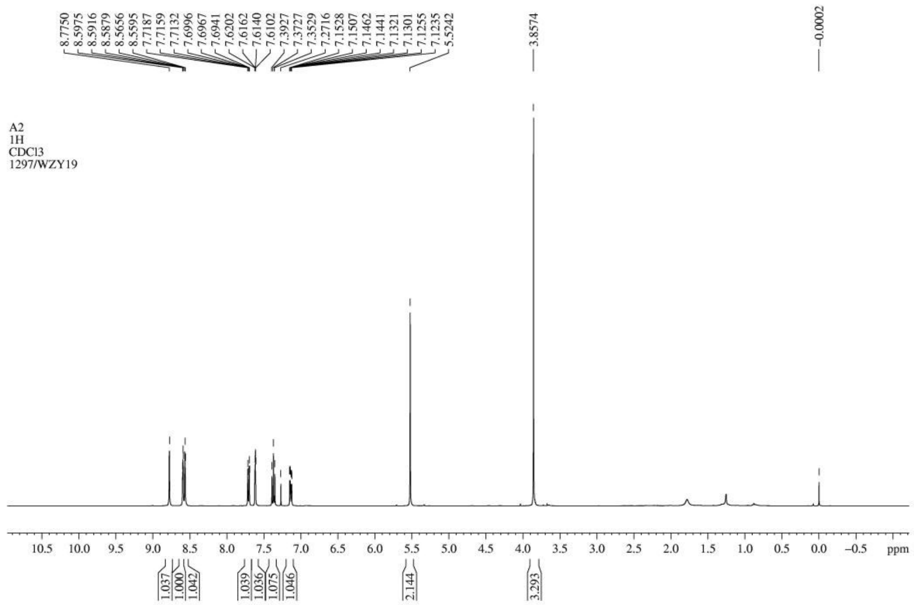

^{1}H NMR spectrum of compound **4f**

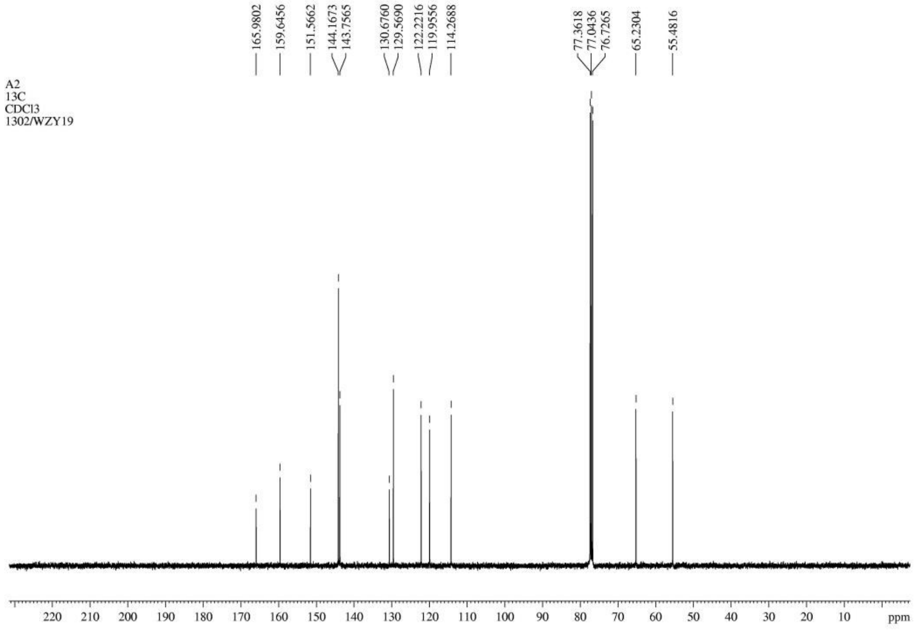

^{13}C NMR spectrum of compound **4f**

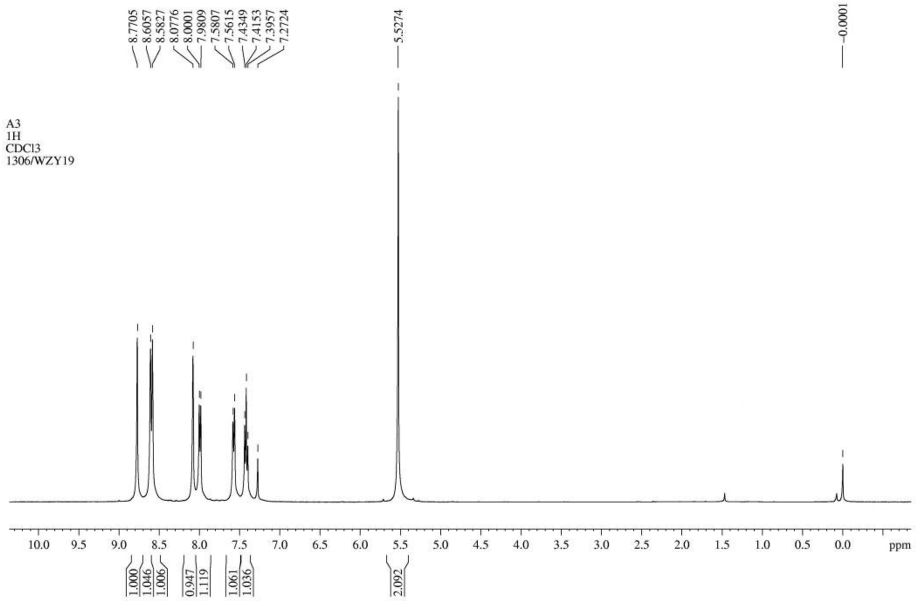

^{1}H NMR spectrum of compound **4g**

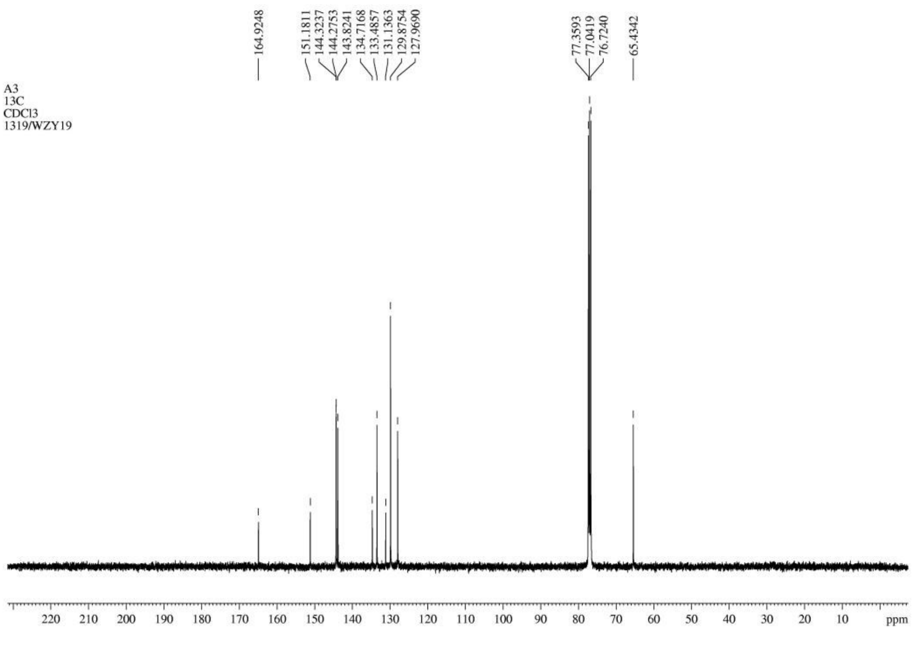

^{13}C NMR spectrum of compound **4g**

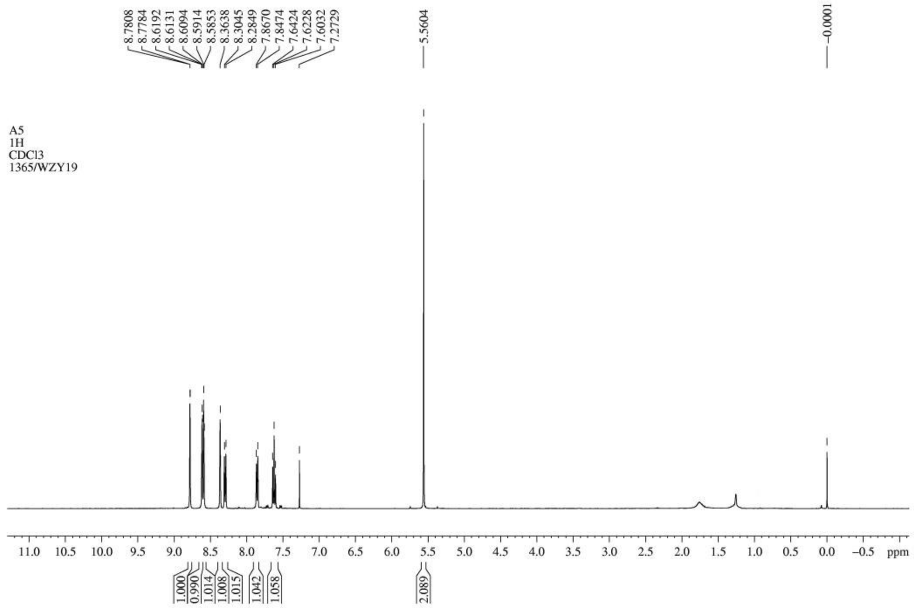

^{1}H NMR spectrum of compound **4h**

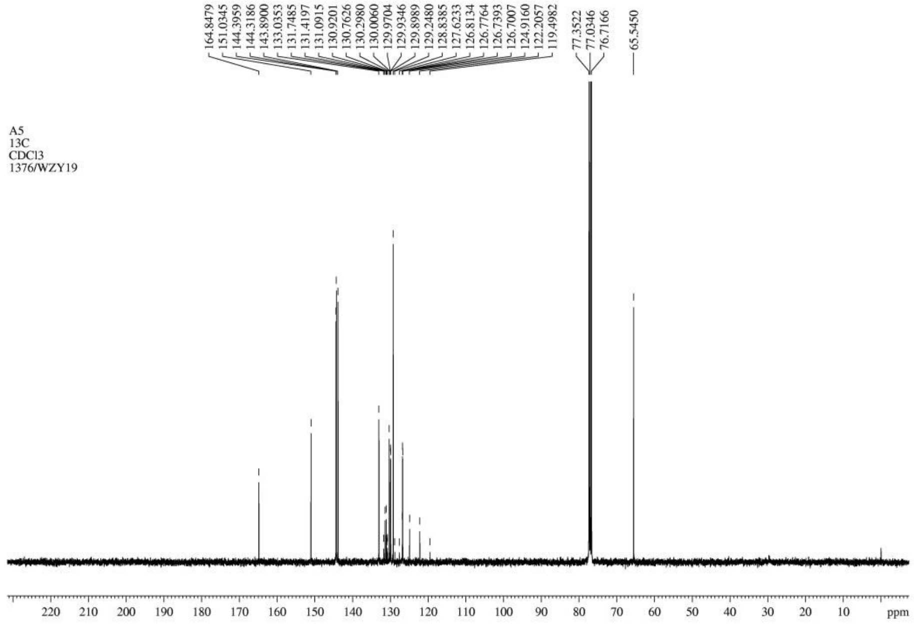

^{13}C NMR spectrum of compound **4h**

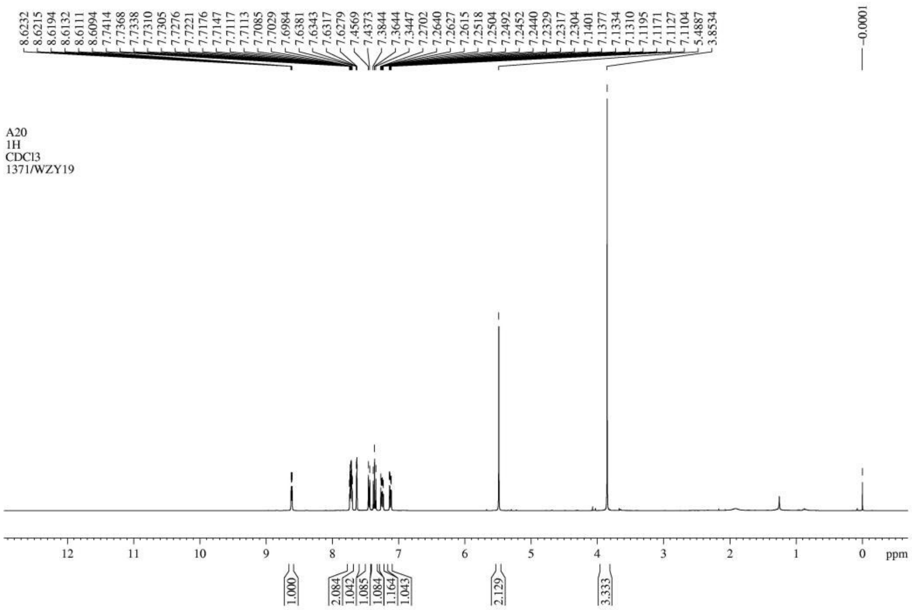

^{1}H NMR spectrum of compound **4i**

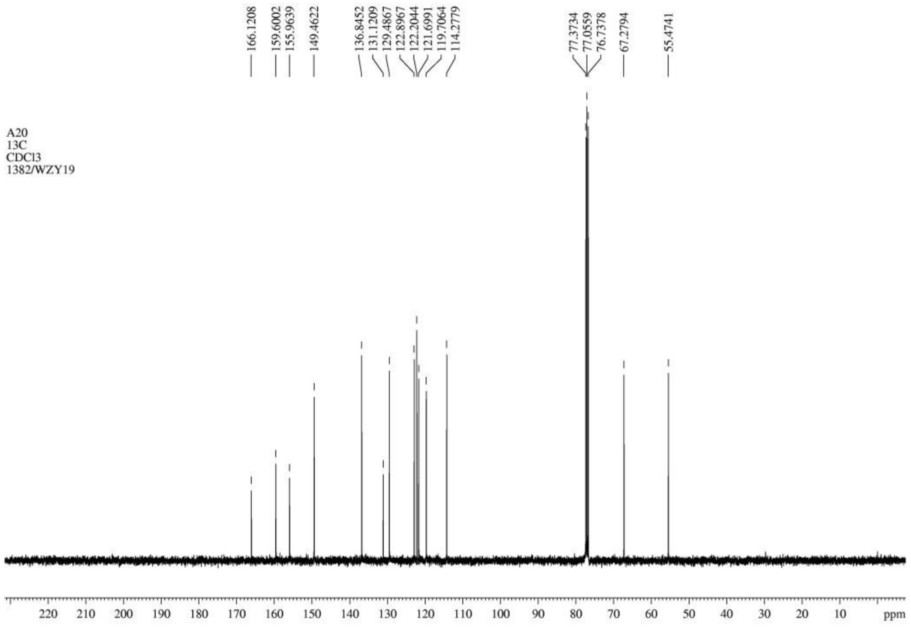

^{13}C NMR spectrum of compound **4i**

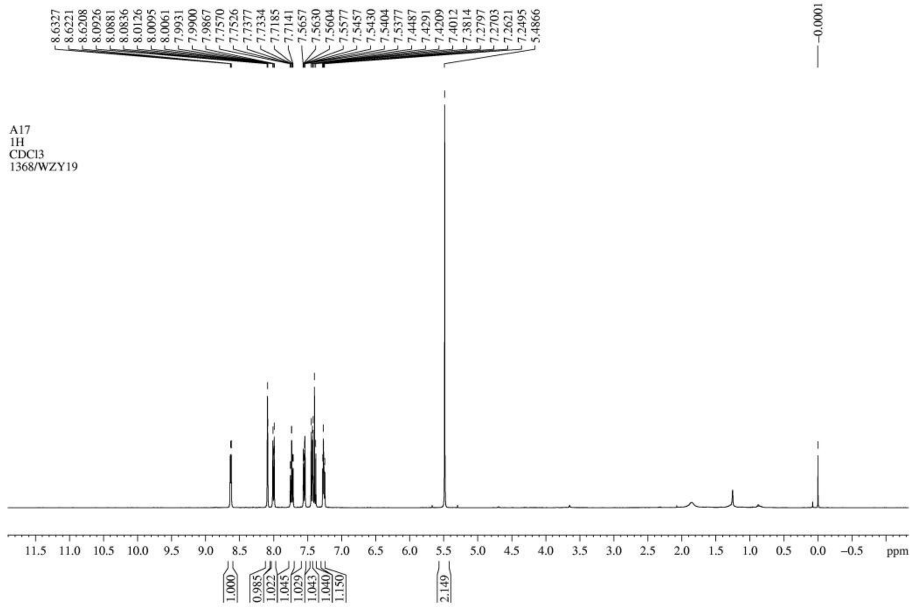

^{1}H NMR spectrum of compound **4j**

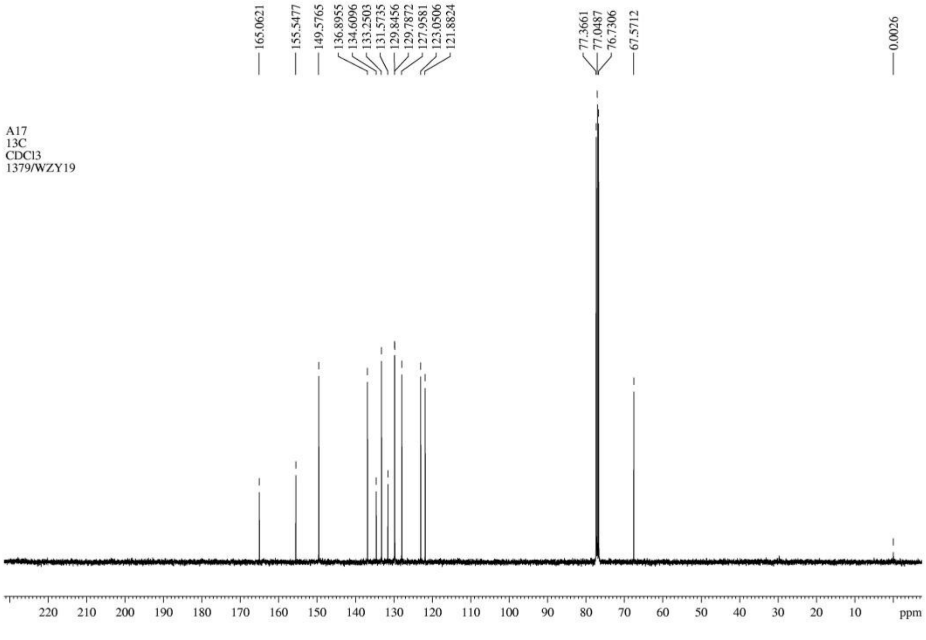

^{13}C NMR spectrum of compound **4j**

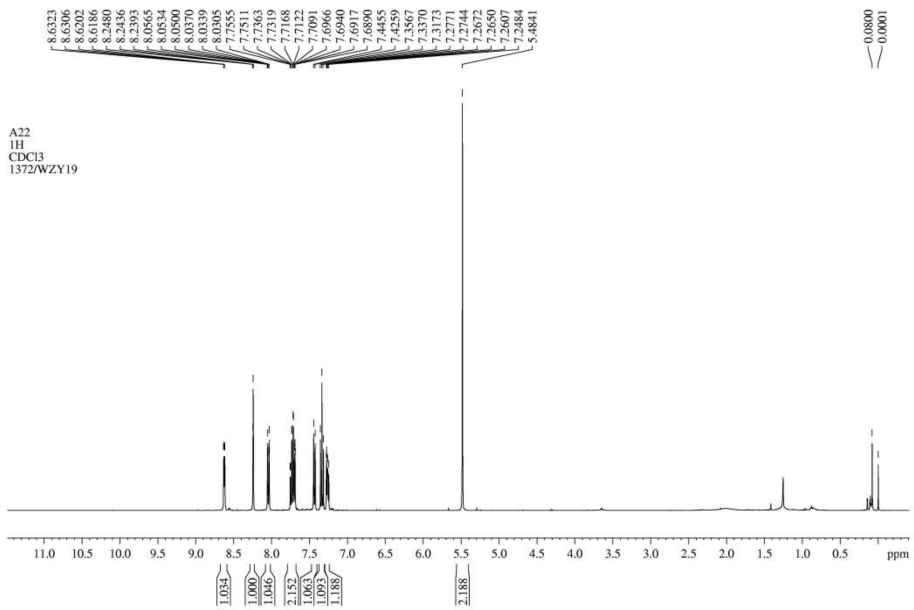

^{1}H NMR spectrum of compound **4k**

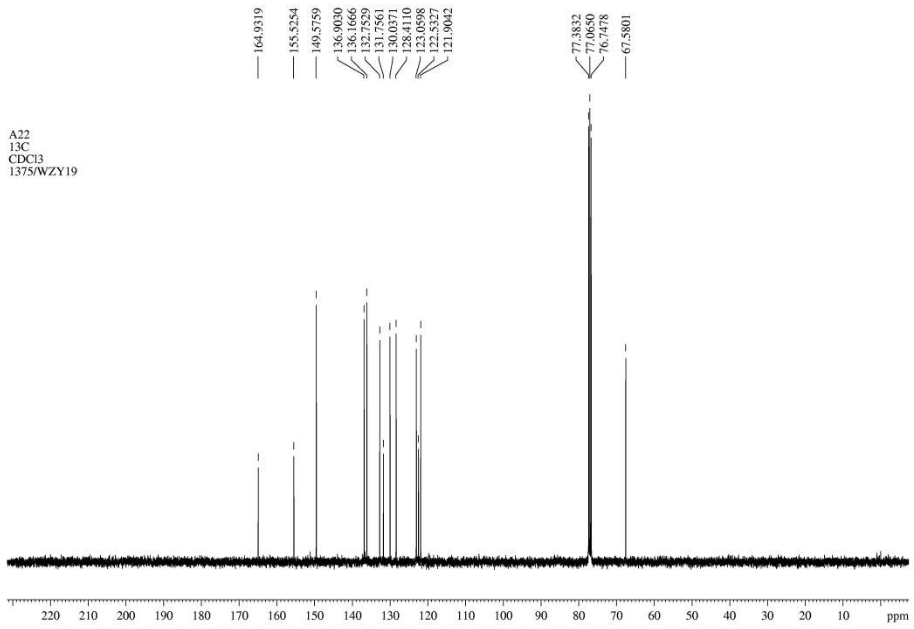

^{13}C NMR spectrum of compound **4k**

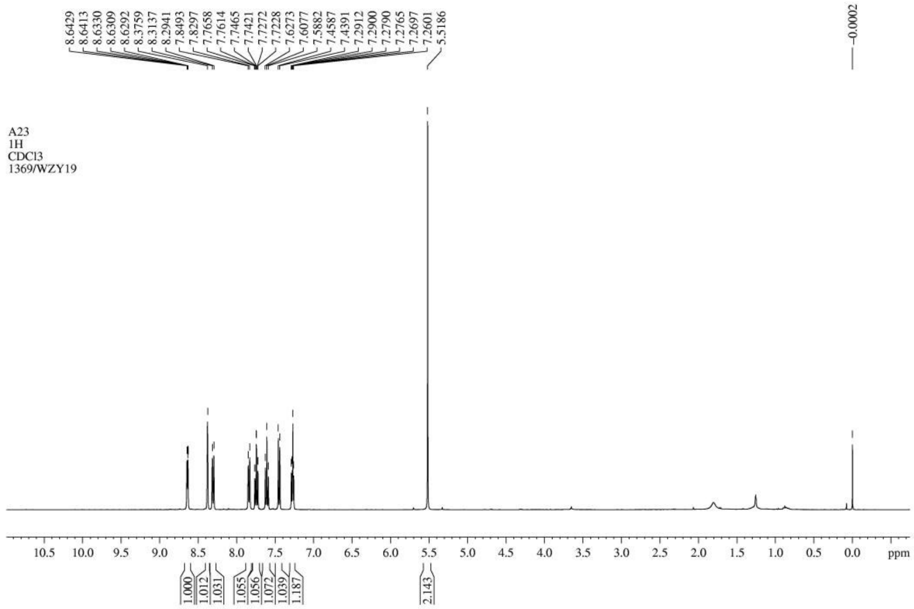

^{1}H NMR spectrum of compound **4l**

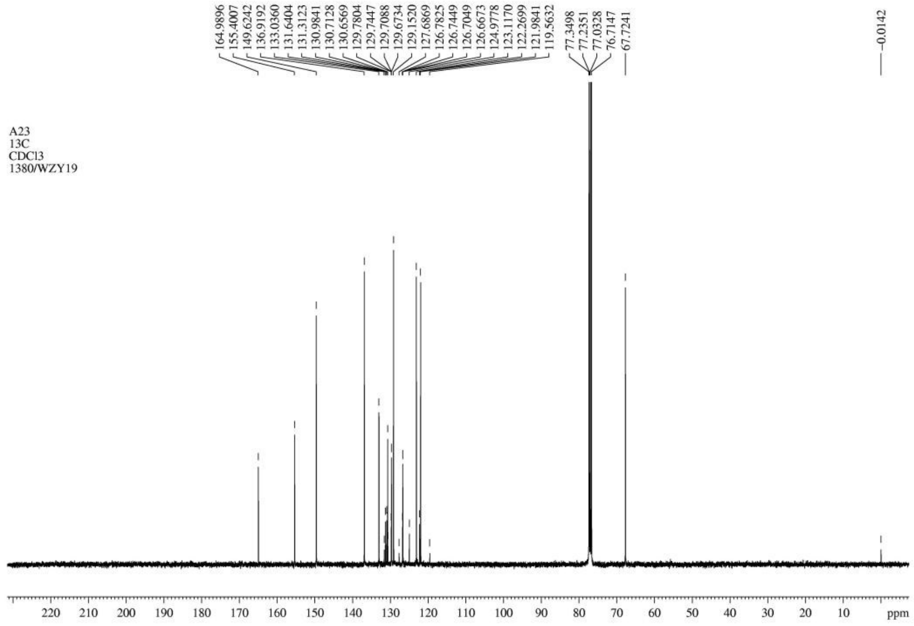

^{13}C NMR spectrum of compound **4l**

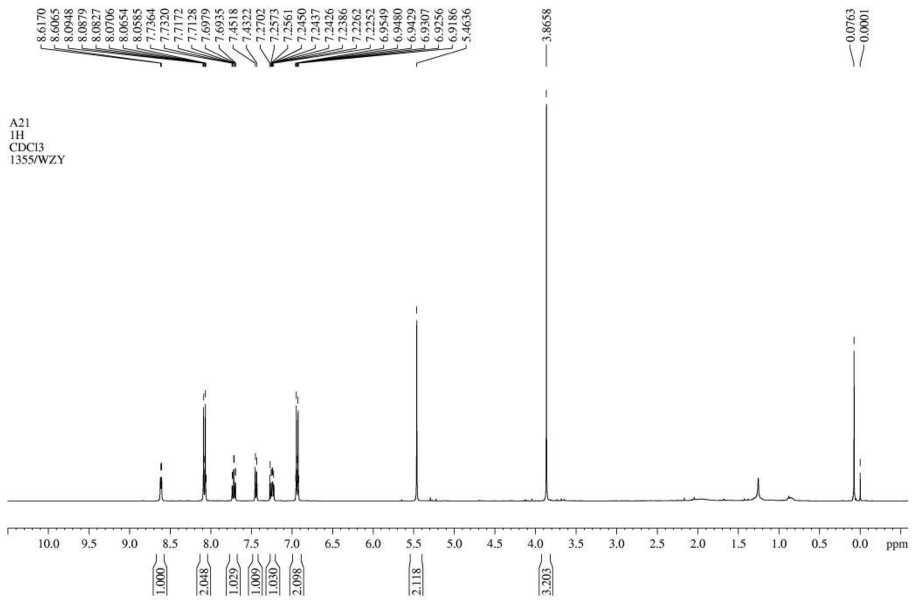

^{1}H NMR spectrum of compound **4m**

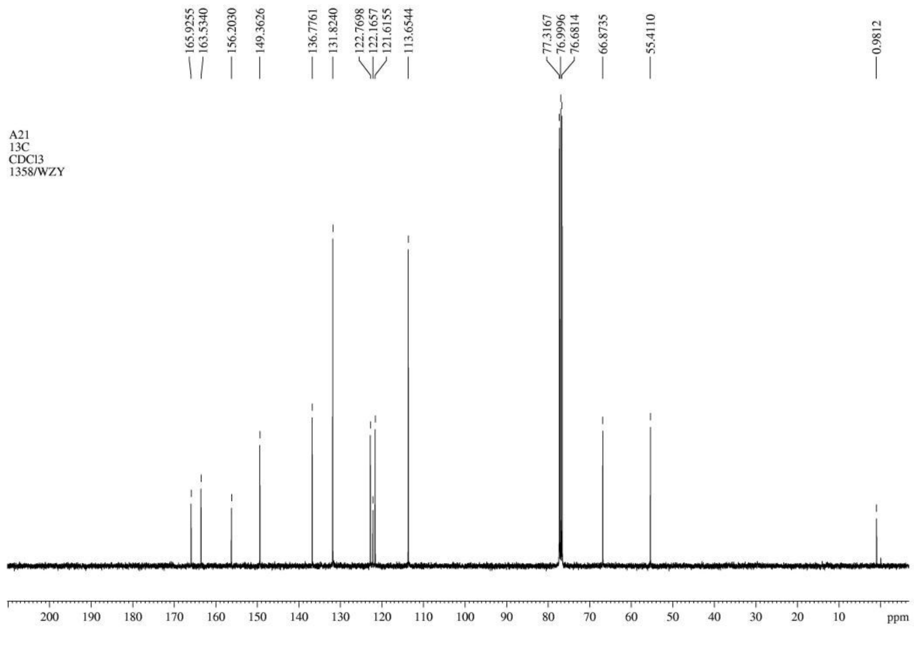

^{13}C NMR spectrum of compound **4m**

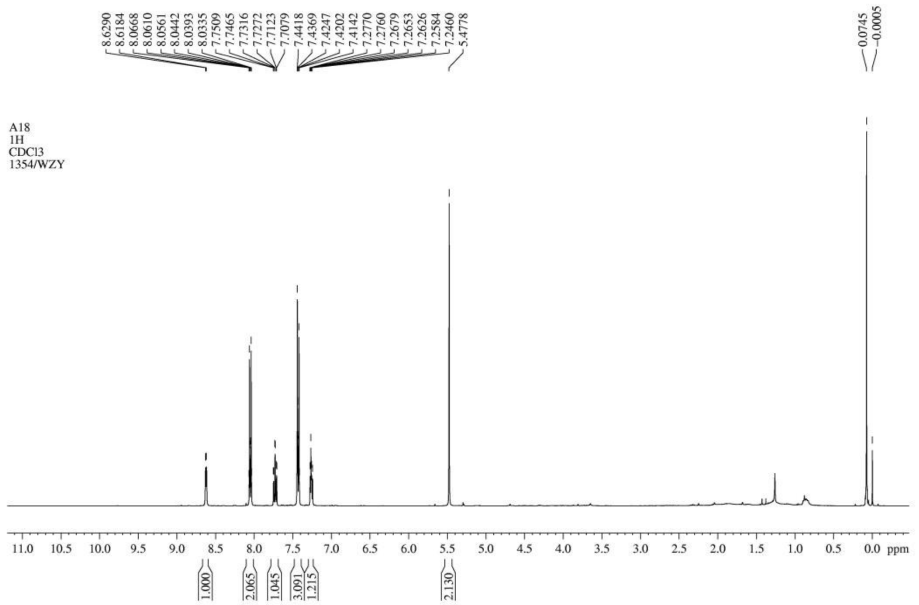

^{1}H NMR spectrum of compound **4n**

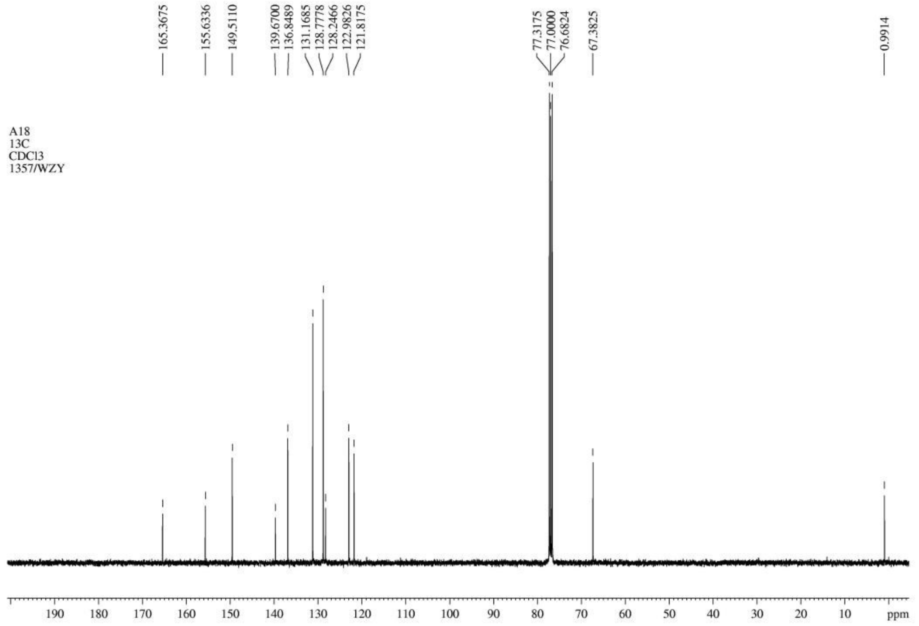

^{13}C NMR spectrum of compound **4n**

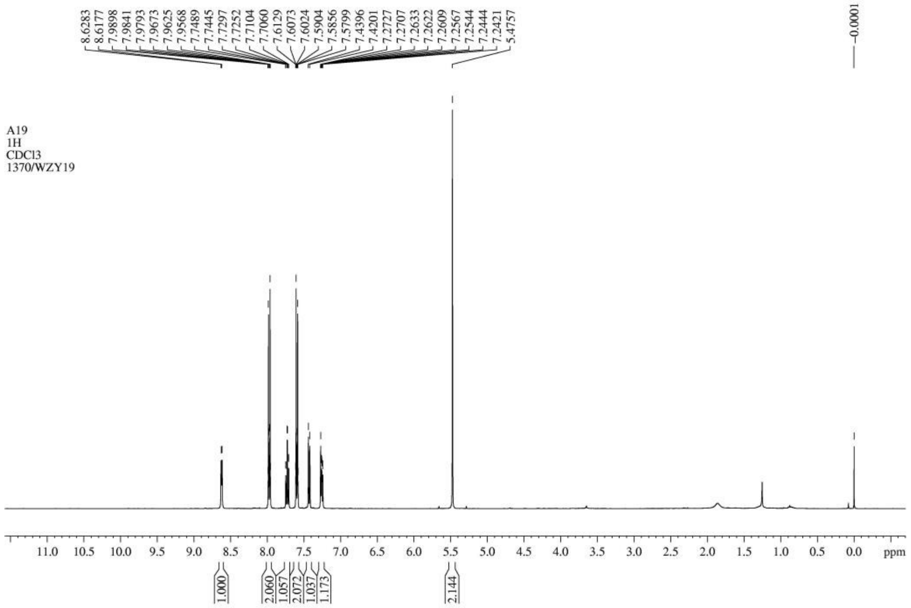

^{1}H NMR spectrum of compound **4o**

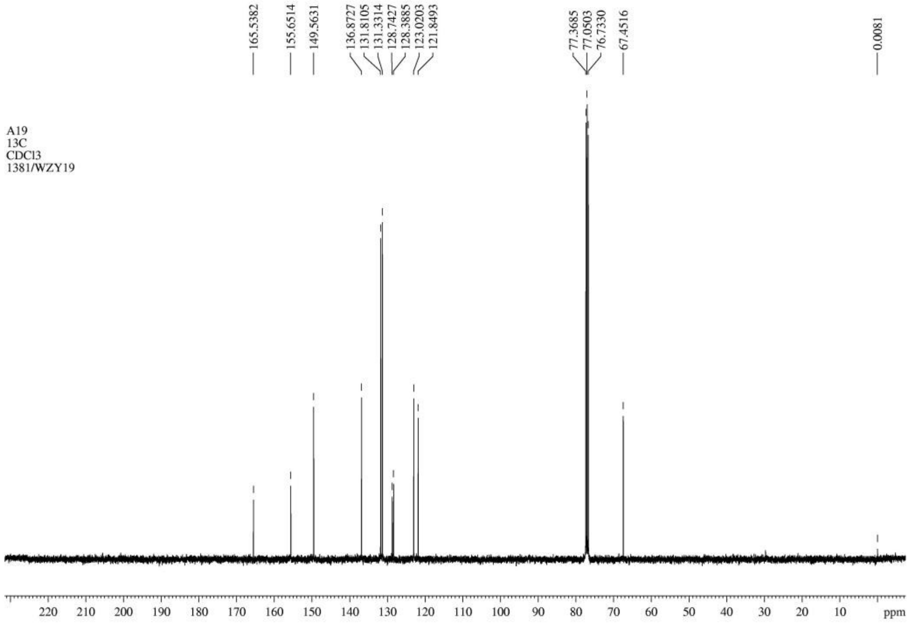

^{13}C NMR spectrum of compound **4o**

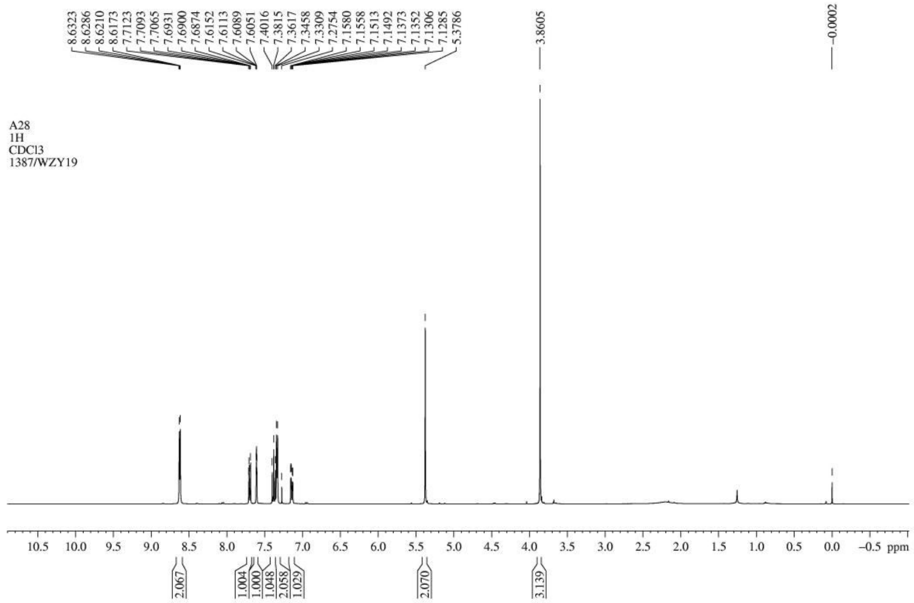

^{1}H NMR spectrum of compound **4p**

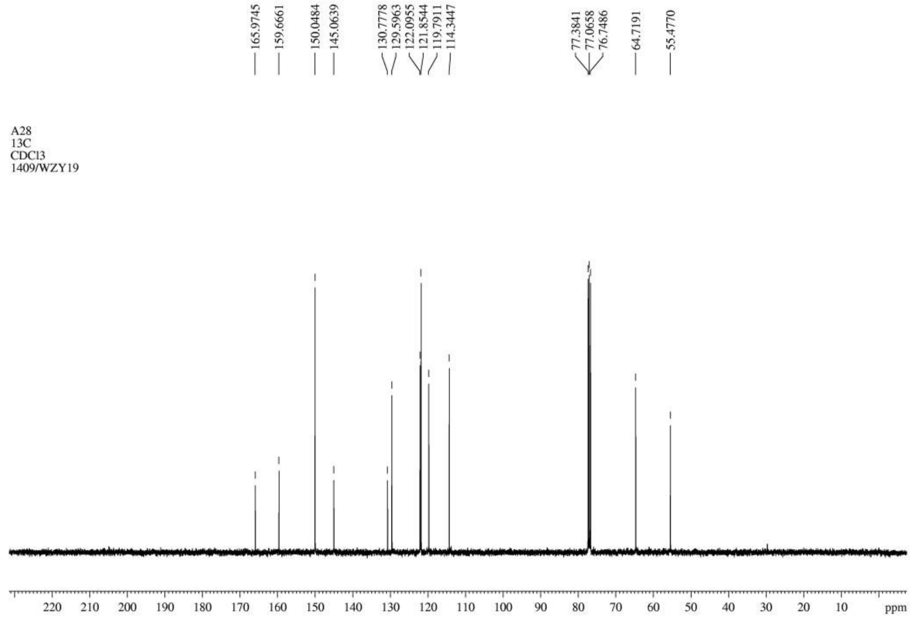

^{13}C NMR spectrum of compound **4p**

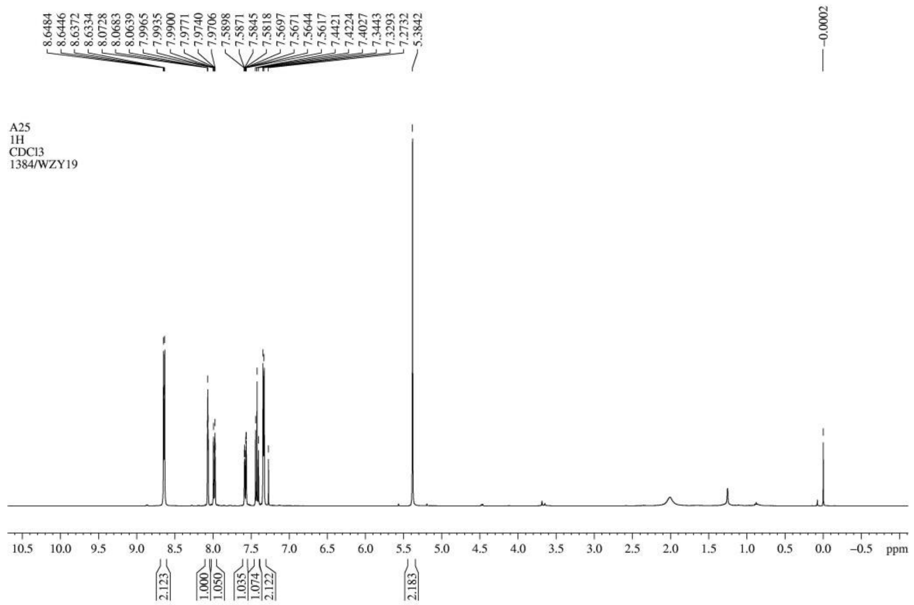

^{1}H NMR spectrum of compound **4q**

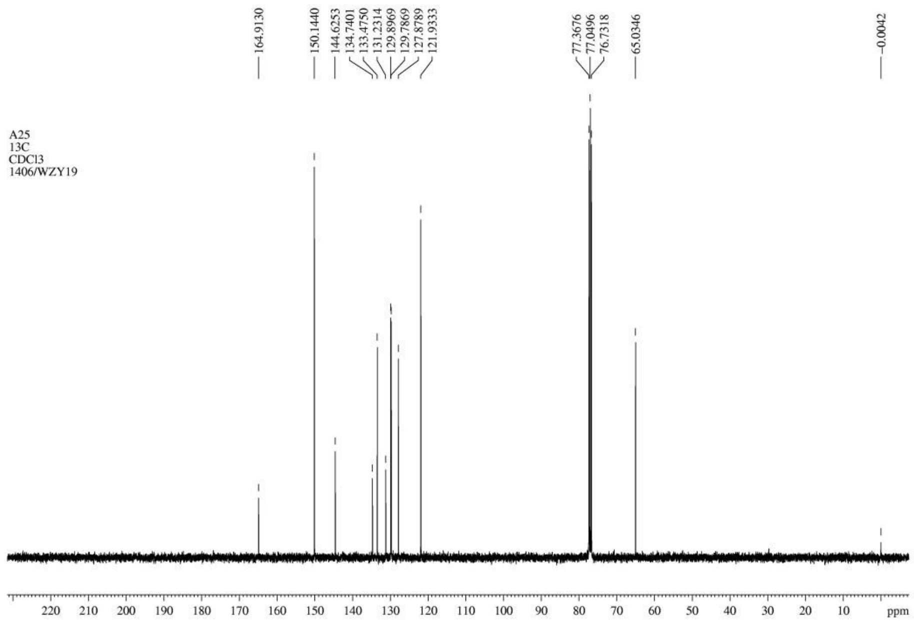

^{13}C NMR spectrum of compound **4q**

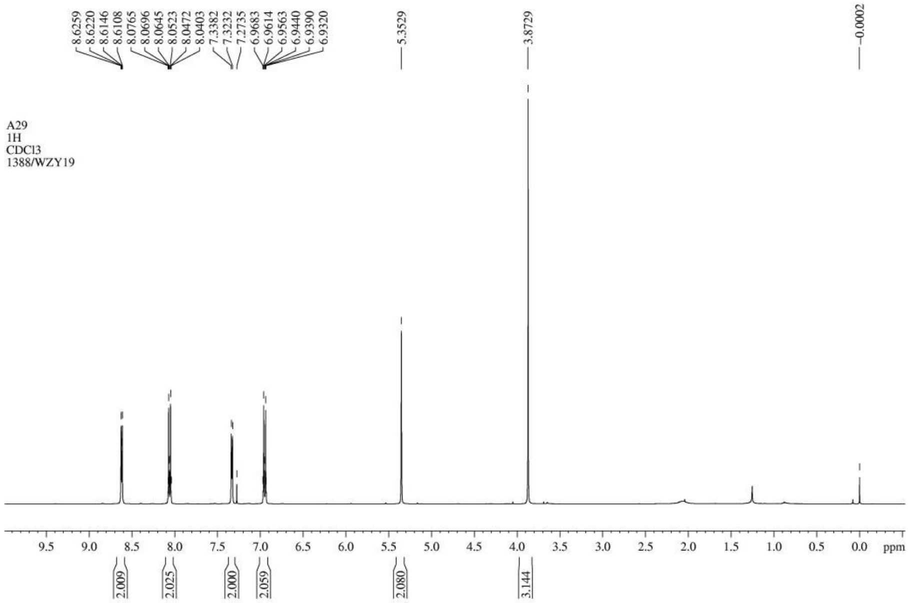

^{1}H NMR spectrum of compound **4r**

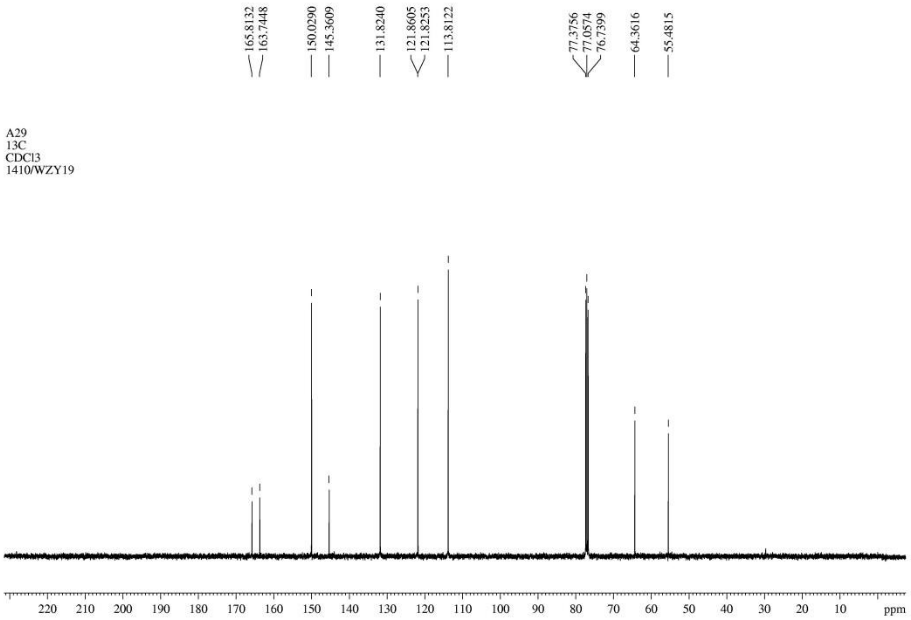

^{13}C NMR spectrum of compound **4r**

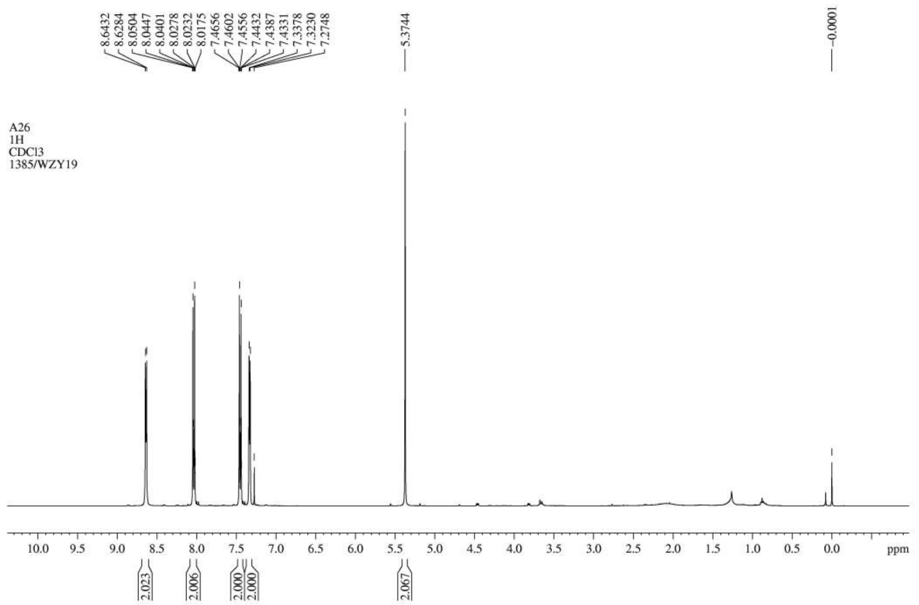

^{1}H NMR spectrum of compound **4s**

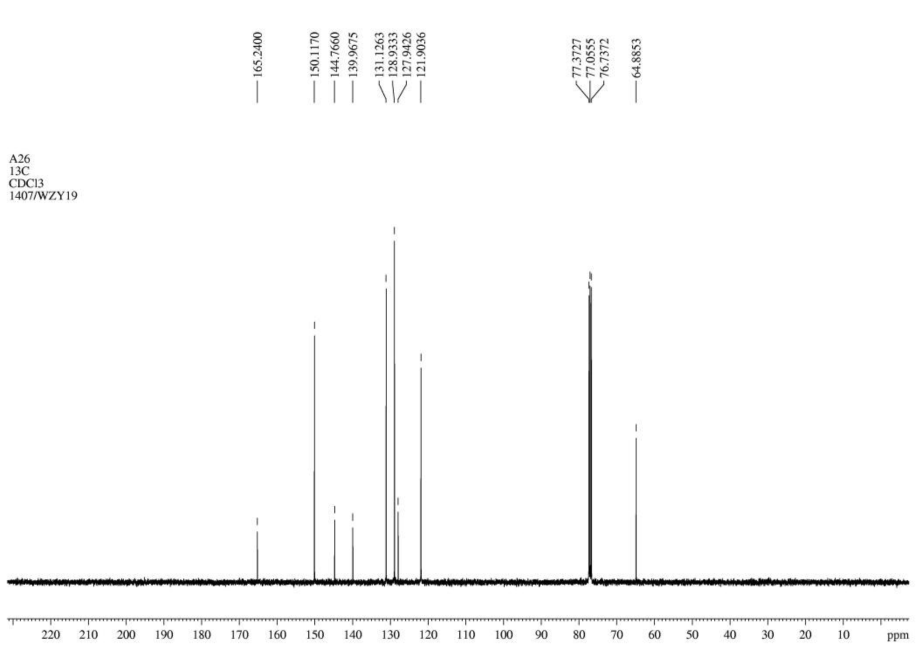

^{13}C NMR spectrum of compound **4s**

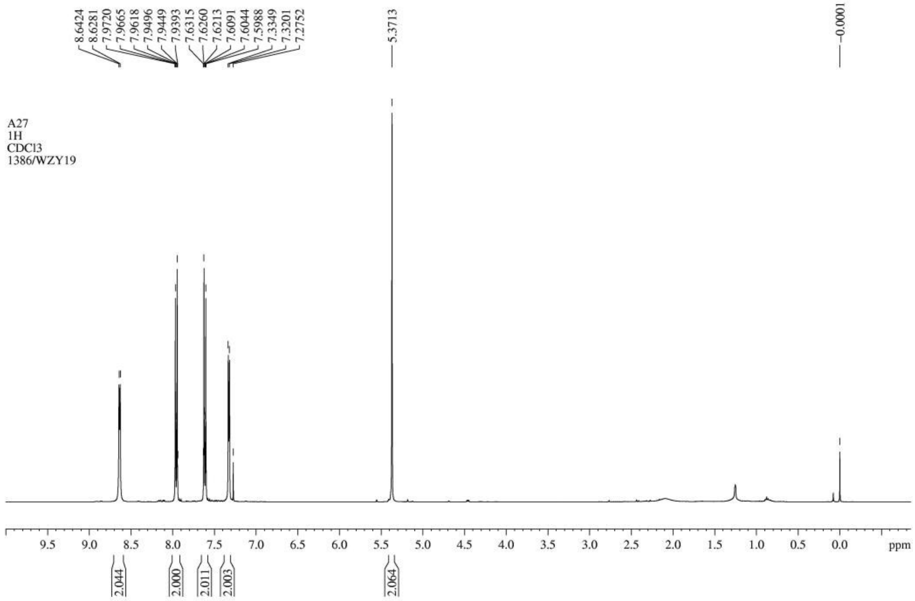

^{1}H NMR spectrum of compound **4t**

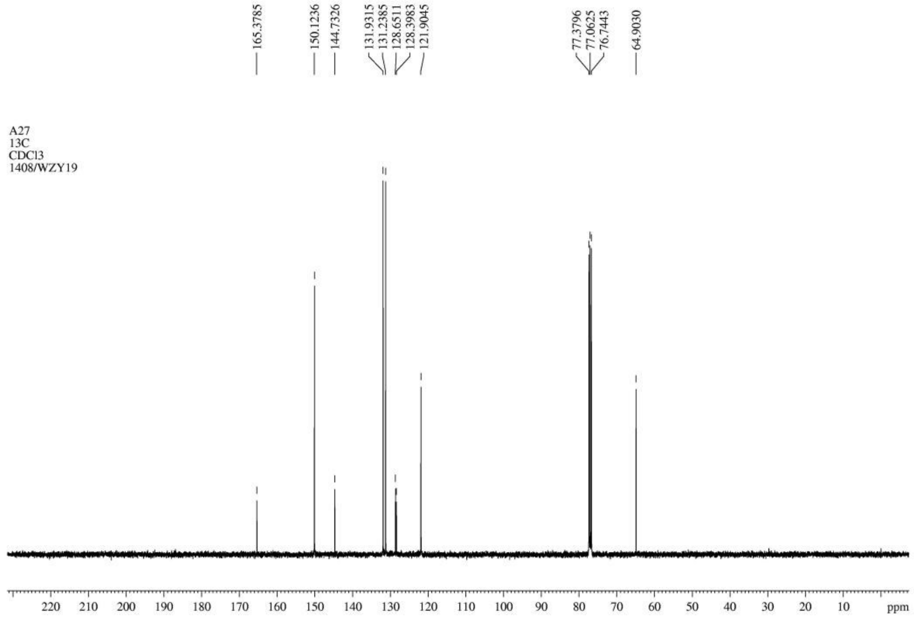

^{13}C NMR spectrum of compound **4t**

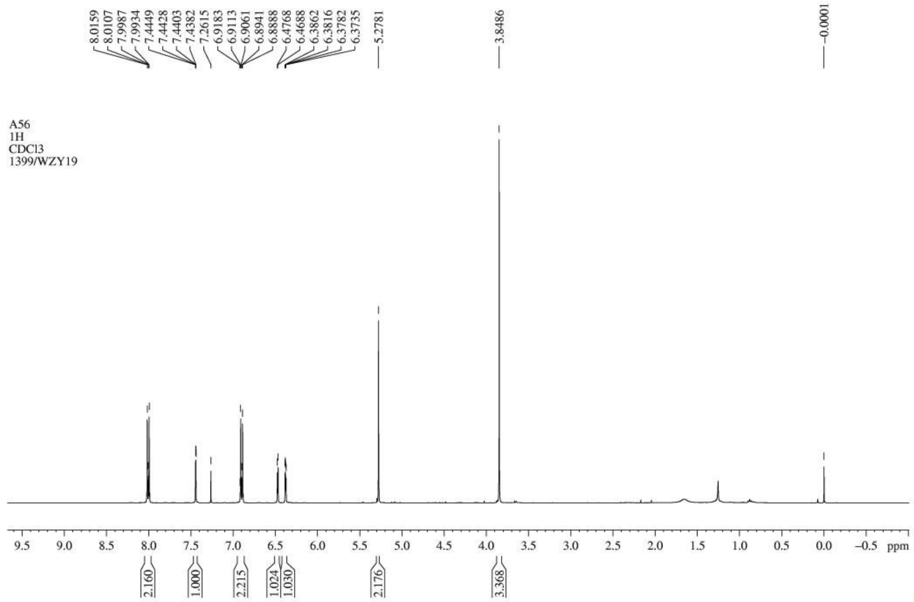

^{1}H NMR spectrum of compound **4u**

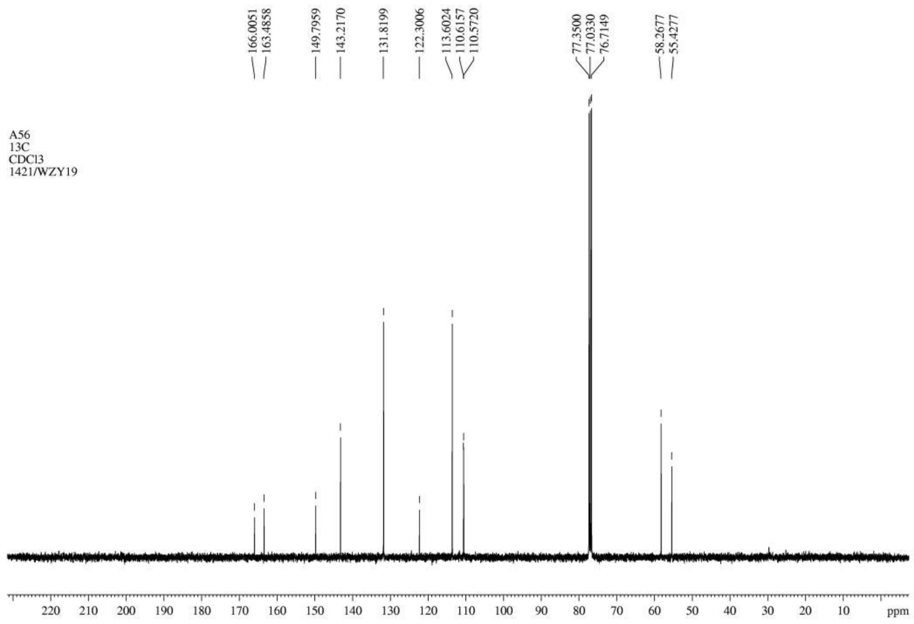

^{13}C NMR spectrum of compound **4u**

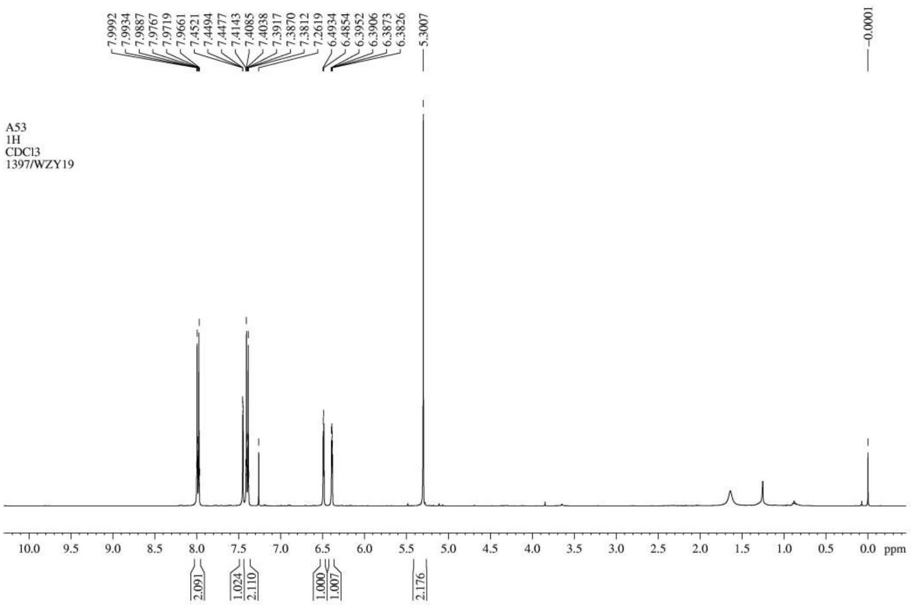

^1H NMR spectrum of compound **4v**

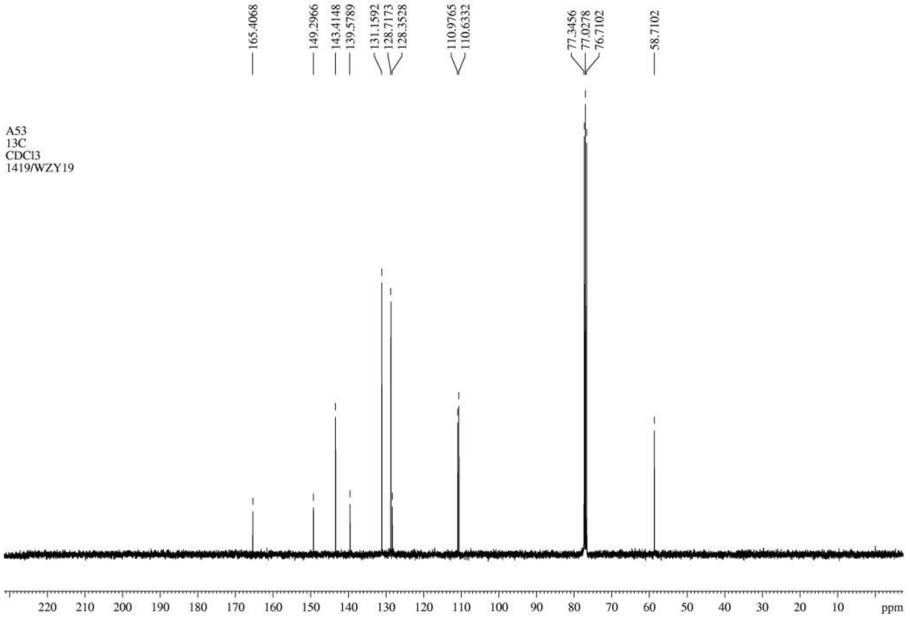

^{13}C NMR spectrum of compound **4v**

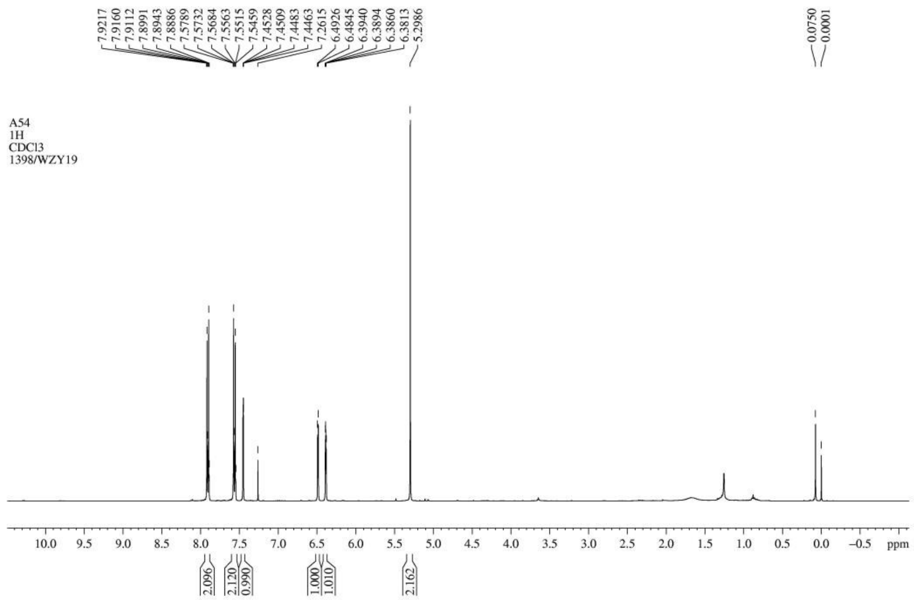

^{1}H NMR spectrum of compound **4w**

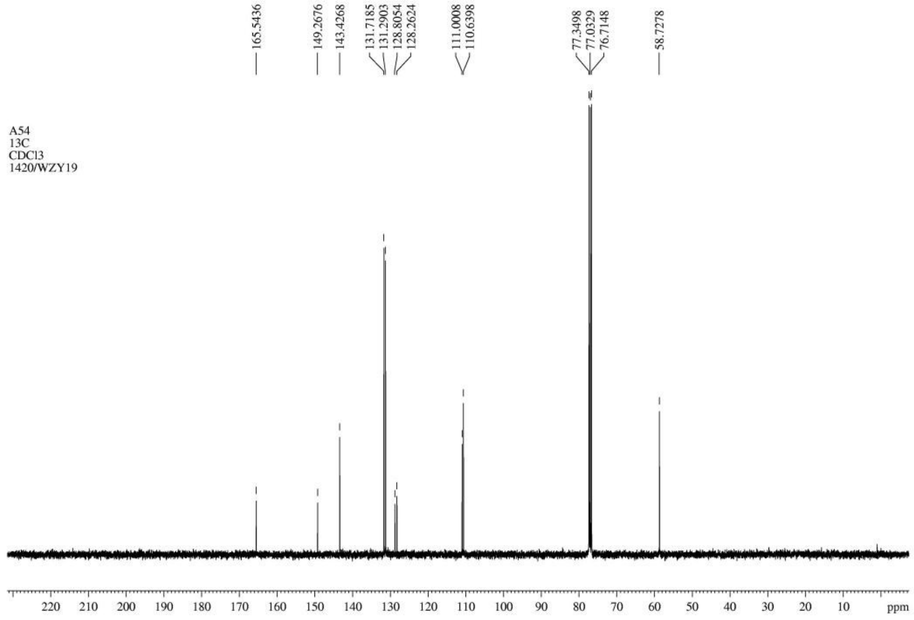

^{13}C NMR spectrum of compound **4w**

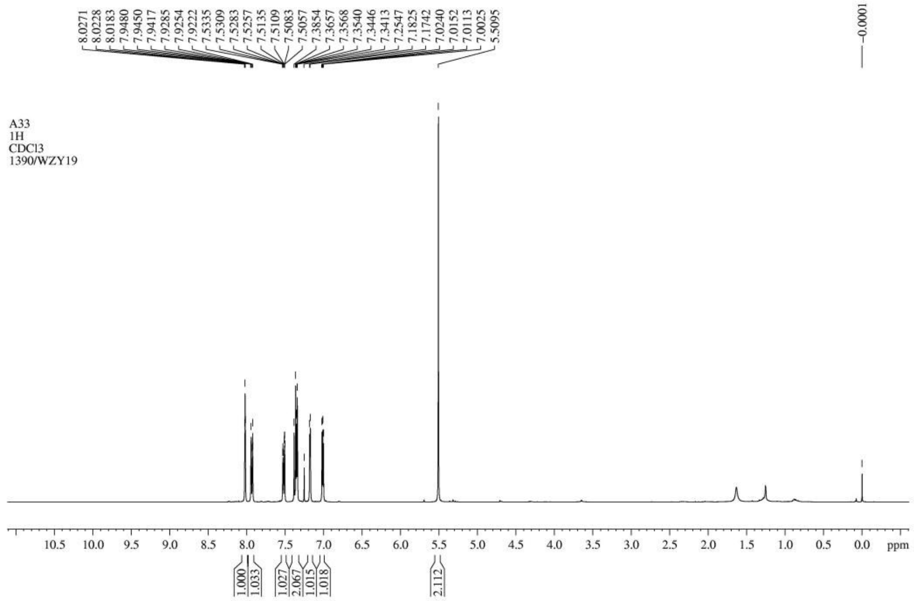

^{1}H NMR spectrum of compound **4x**

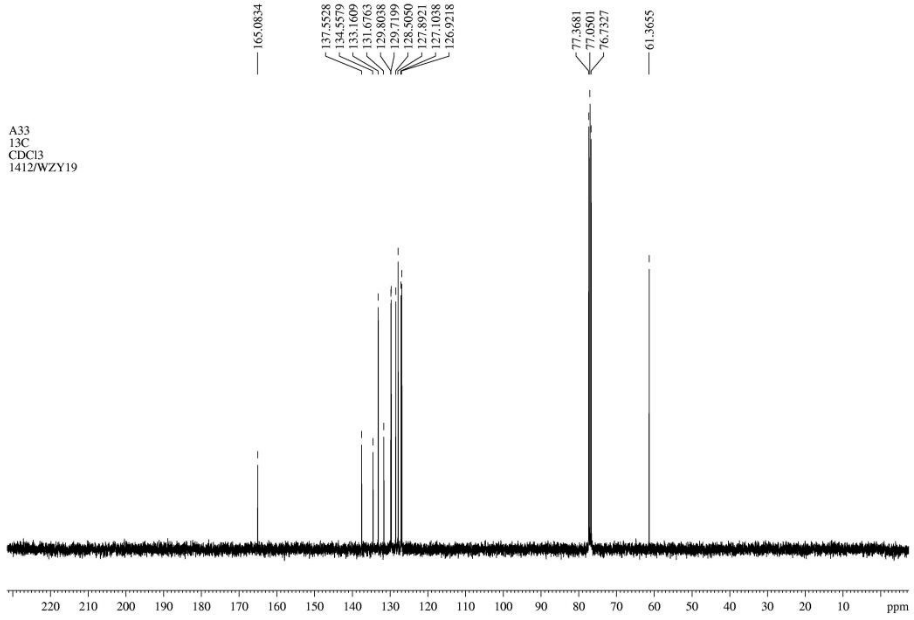

^{13}C NMR spectrum of compound **4x**

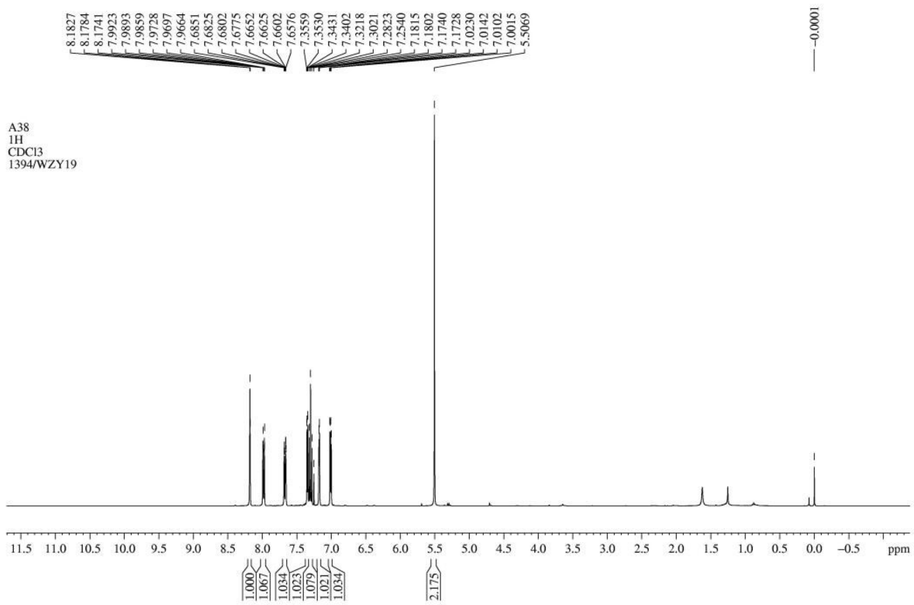

^{1}H NMR spectrum of compound **4y**

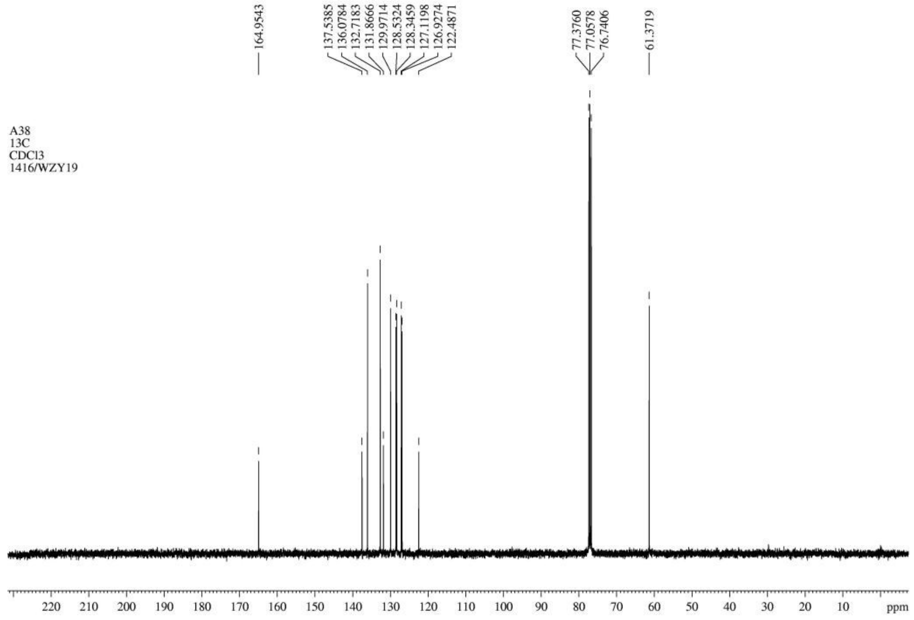

^{13}C NMR spectrum of compound **4y**

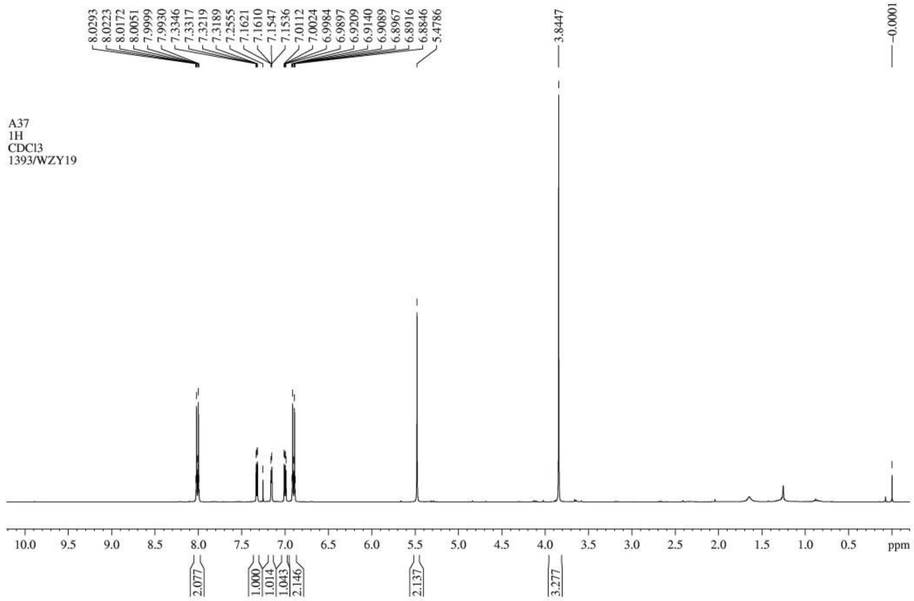

^{1}H NMR spectrum of compound **4z**

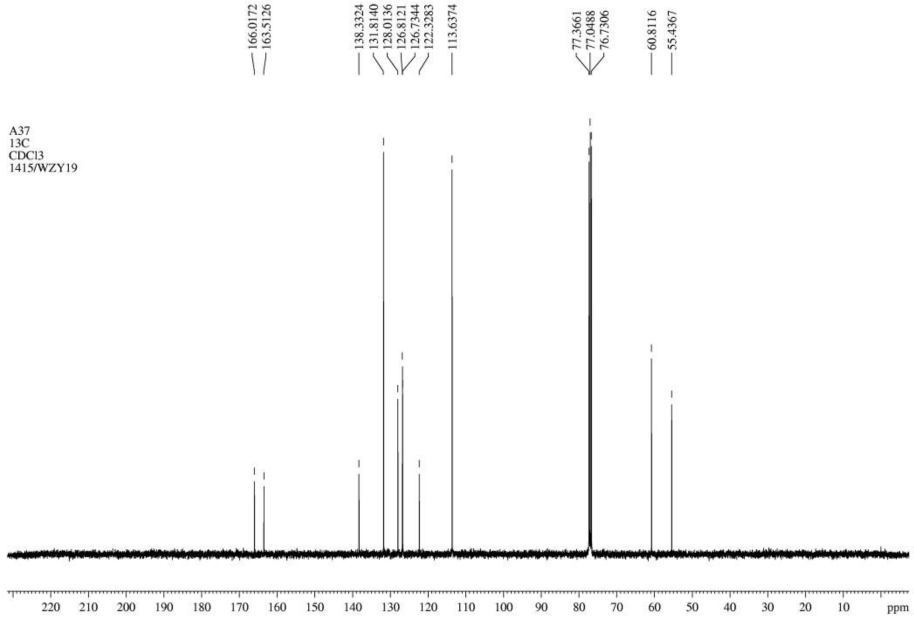

^{13}C NMR spectrum of compound **4z**

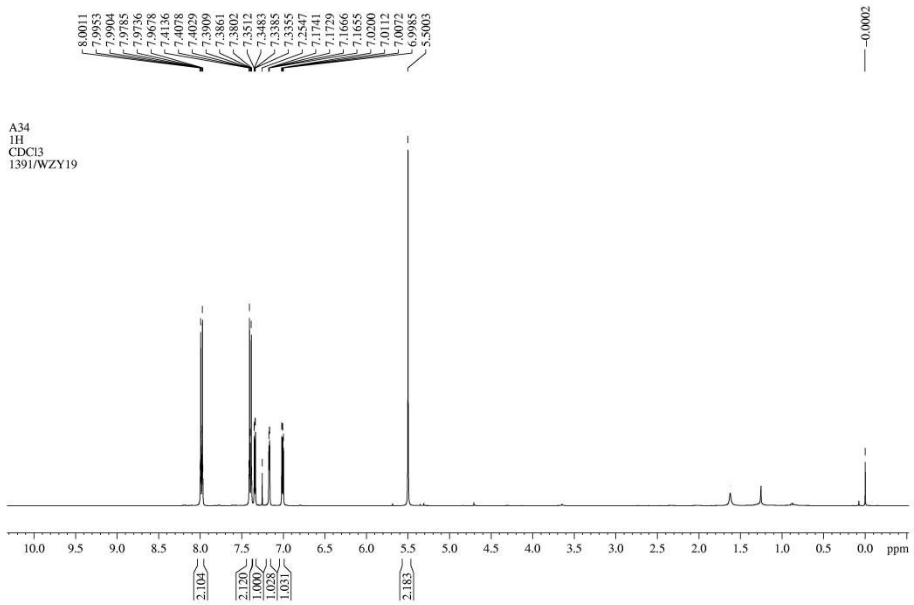

^{1}H NMR spectrum of compound **4a'**

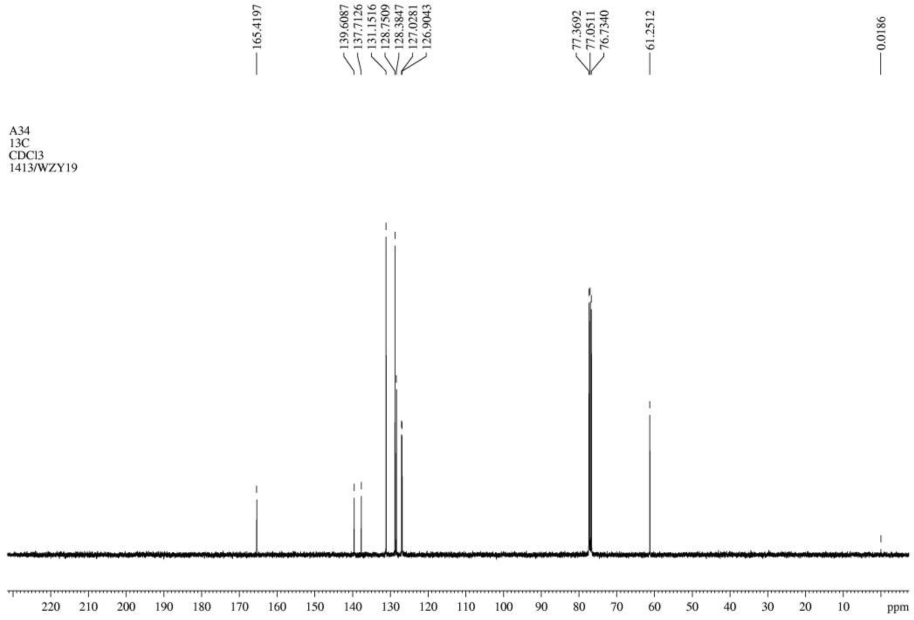

^{13}C NMR spectrum of compound **4a'**

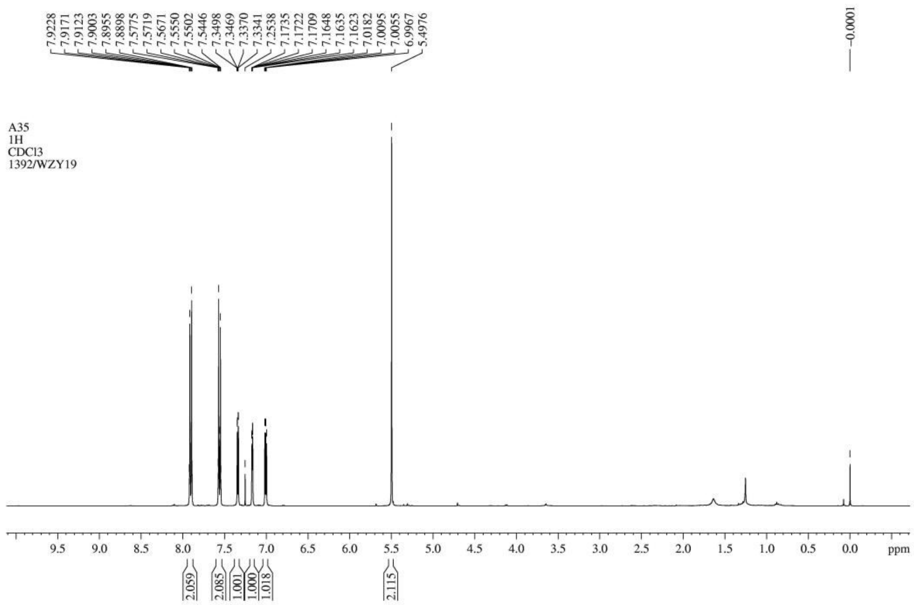

^{1}H NMR spectrum of compound **4b'**

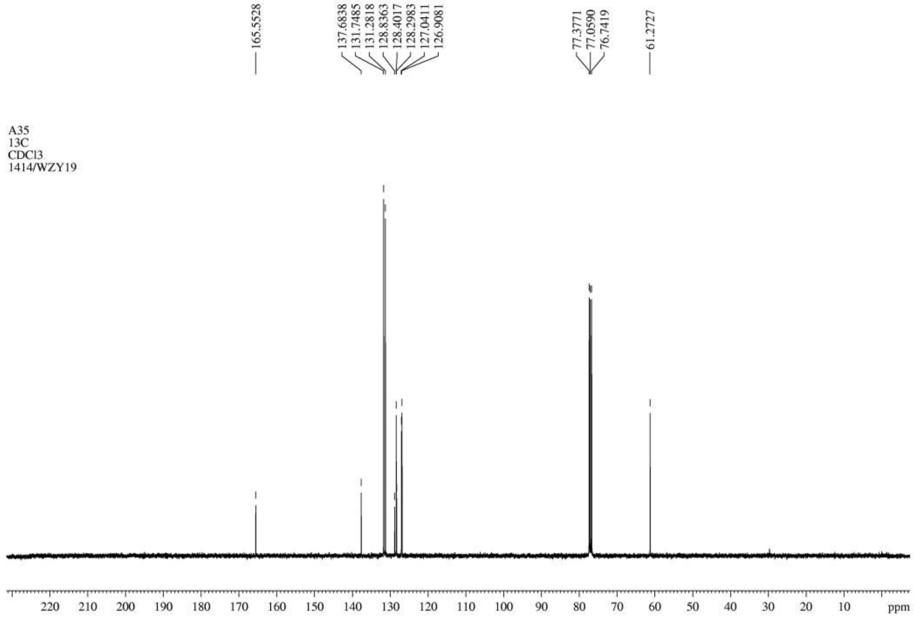

^{13}C NMR spectrum of compound **4b'**

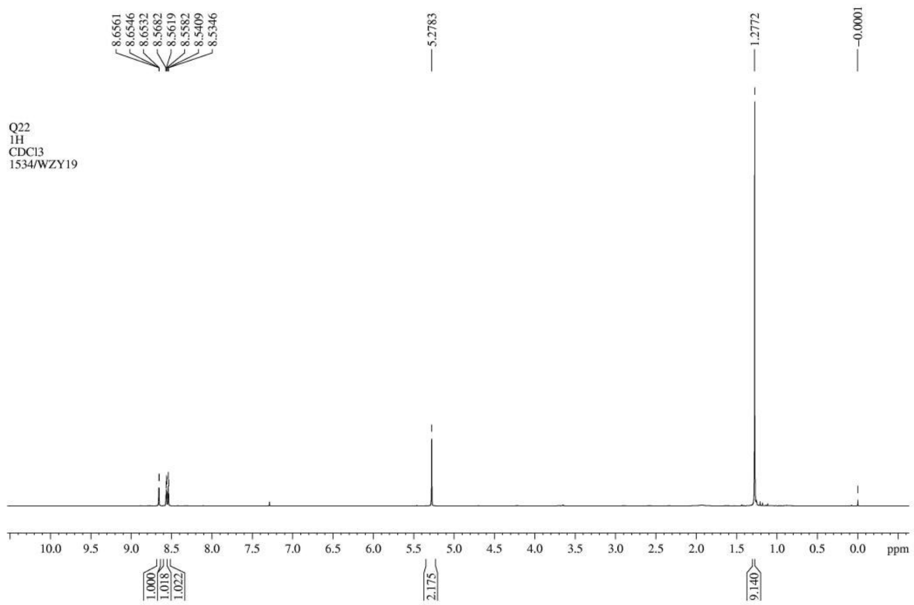

^{1}H NMR spectrum of compound **4c'**

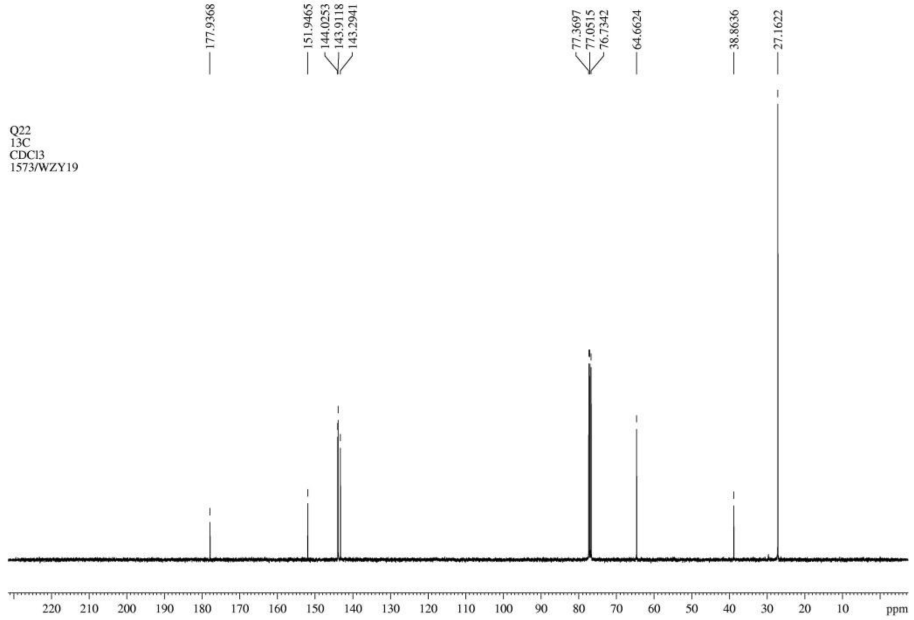

^{13}C NMR spectrum of compound **4c'**

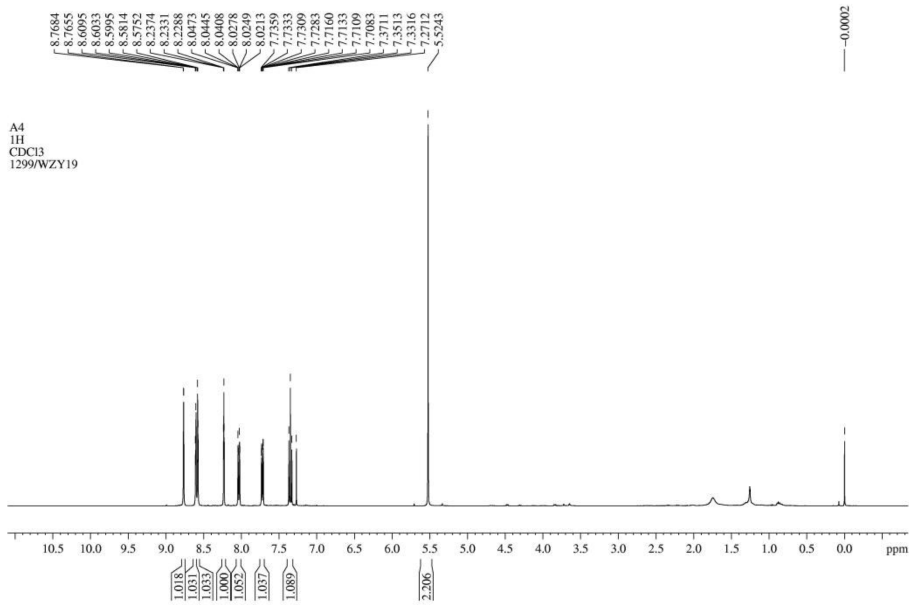

^{1}H NMR spectrum of compound **5a**

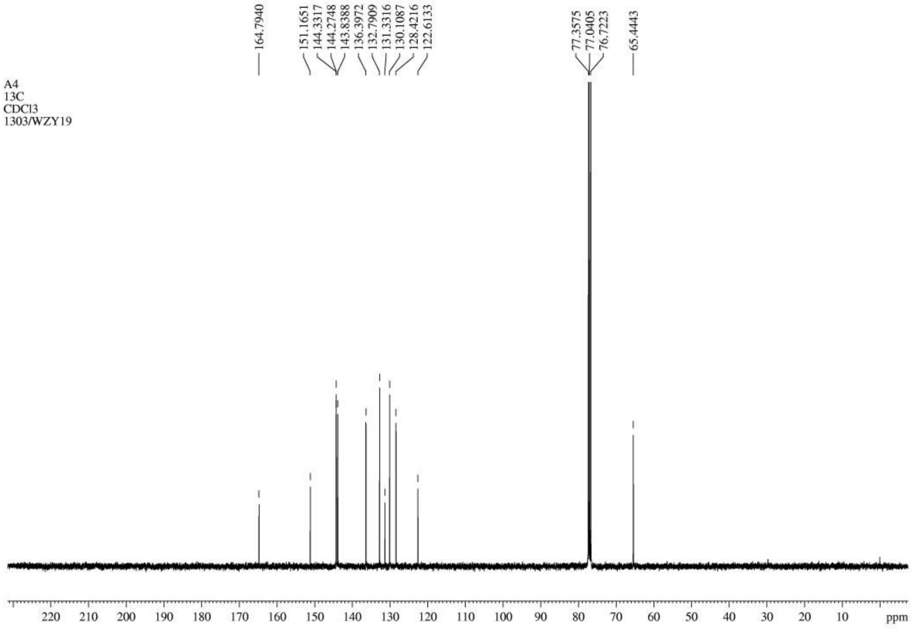

^{13}C NMR spectrum of compound **5a**

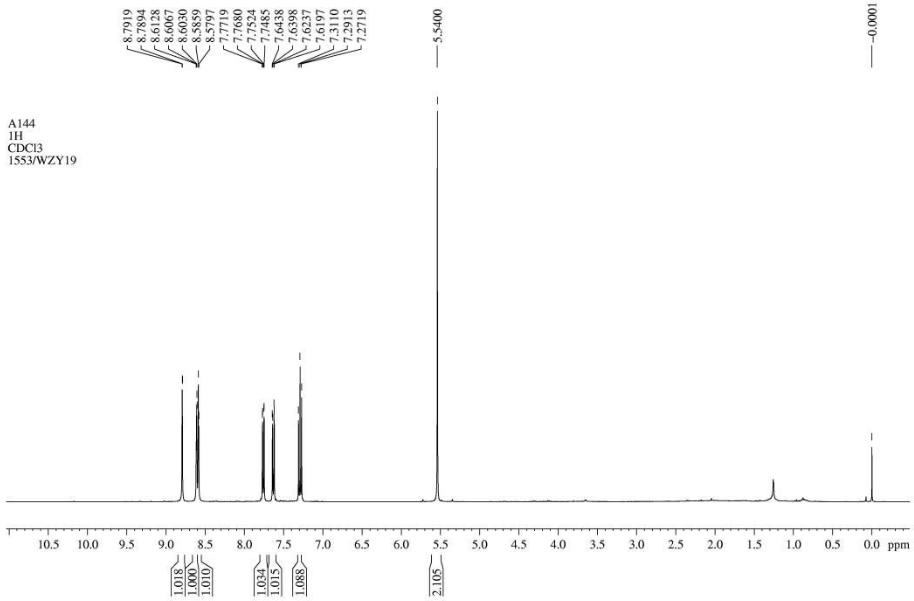

^{1}H NMR spectrum of compound **5b**

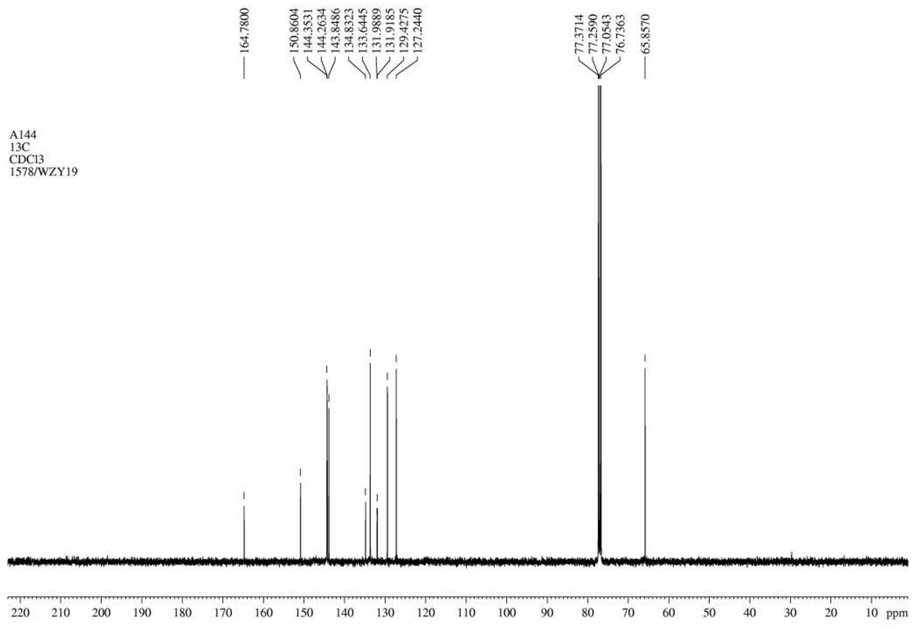

^{13}C NMR spectrum of compound **5b**

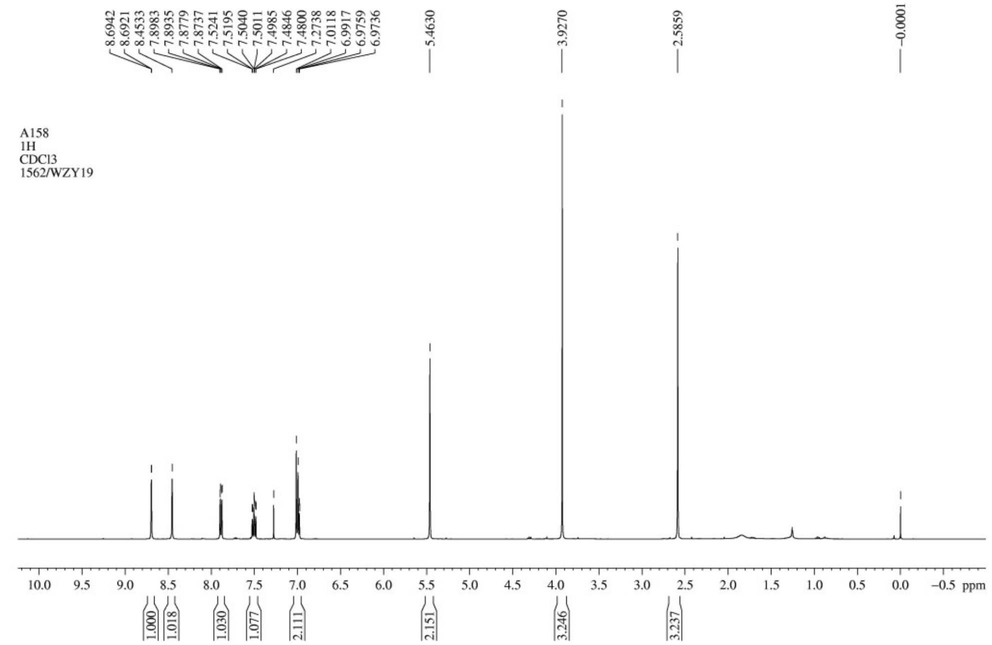

^{1}H NMR spectrum of compound **5c**

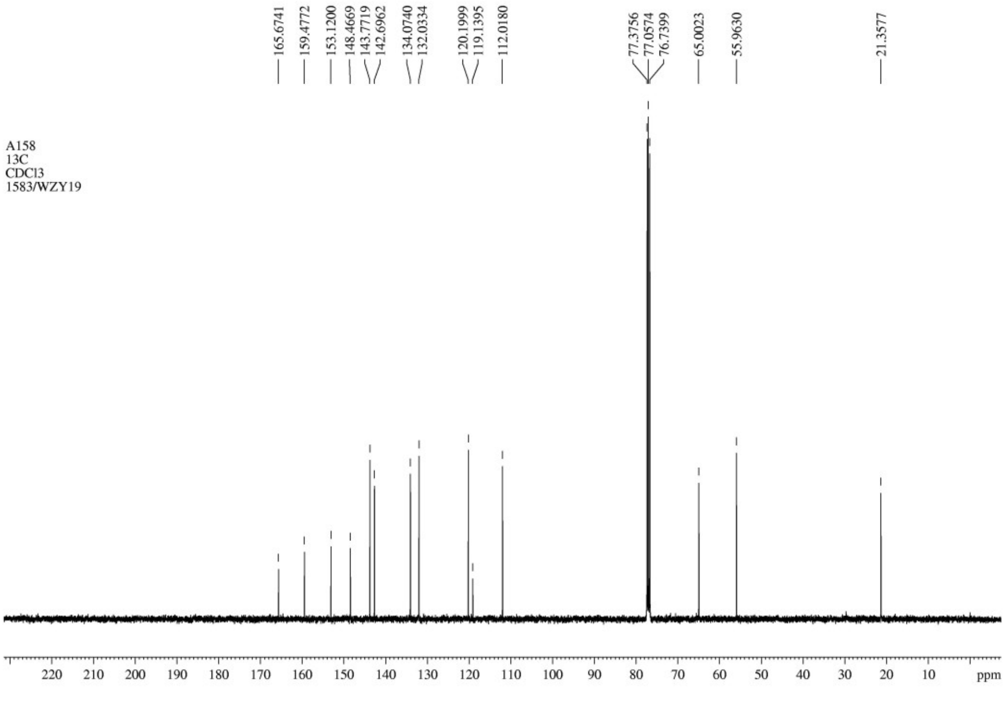

^{13}C NMR spectrum of compound **5c**

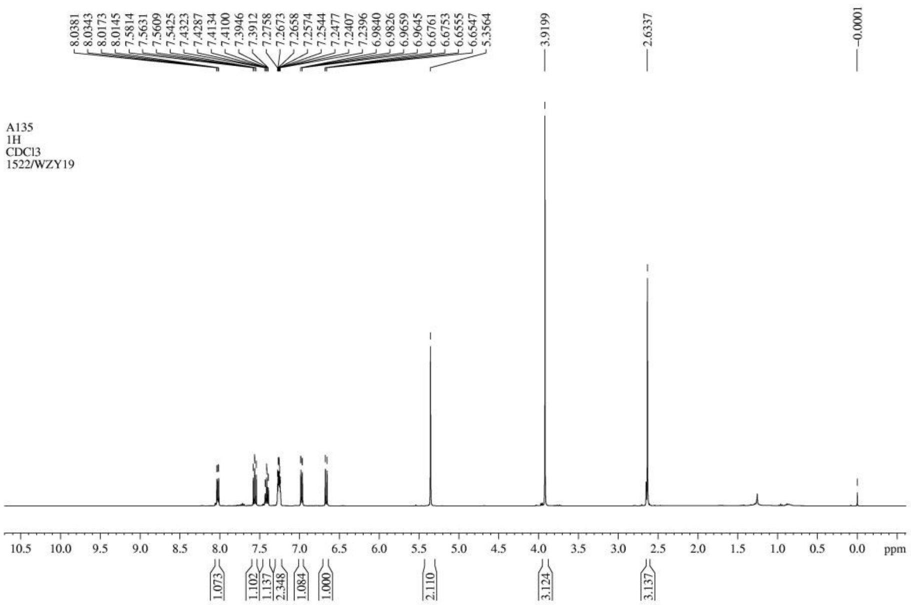

^{1}H NMR spectrum of compound **5d**

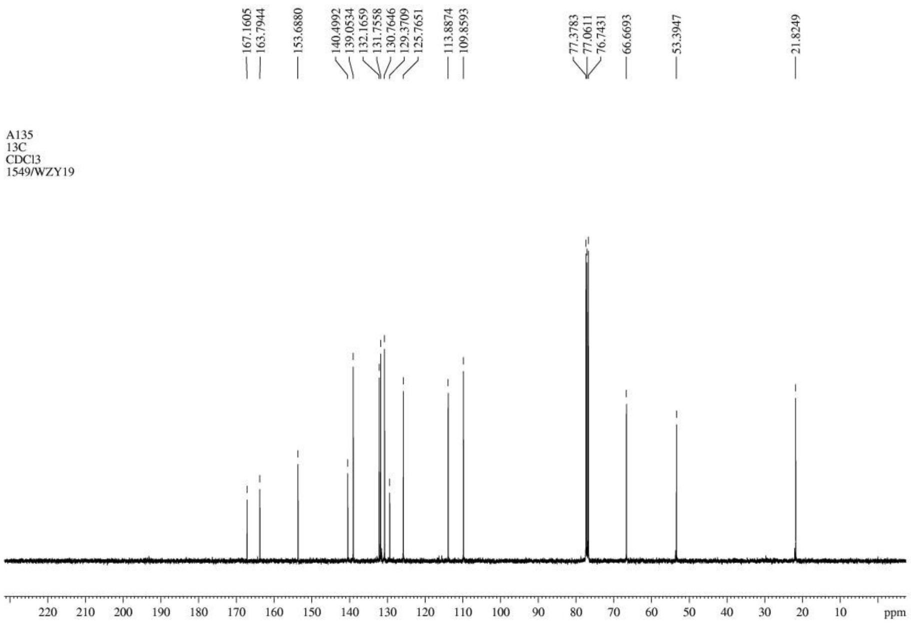

^{13}C NMR spectrum of compound **5d**

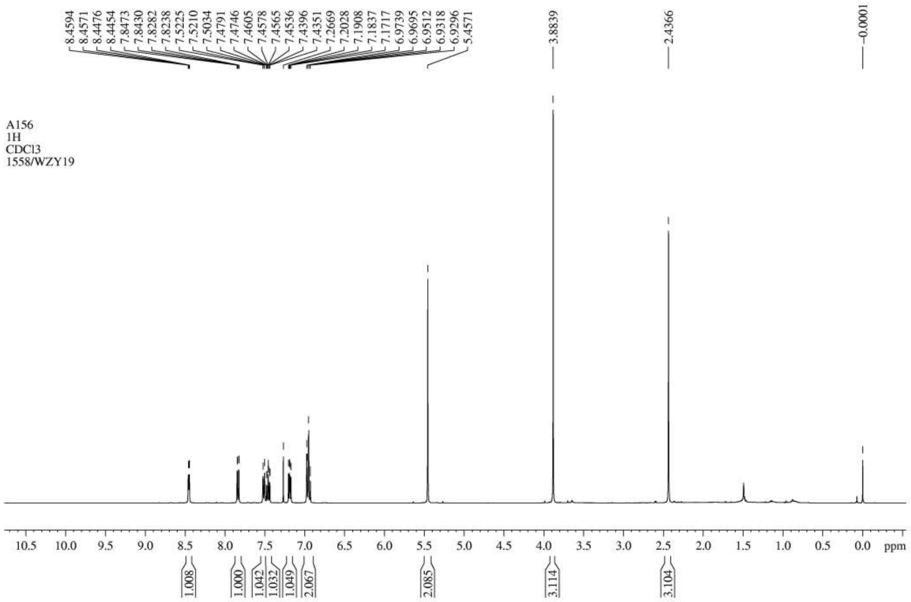

^{1}H NMR spectrum of compound **5e**

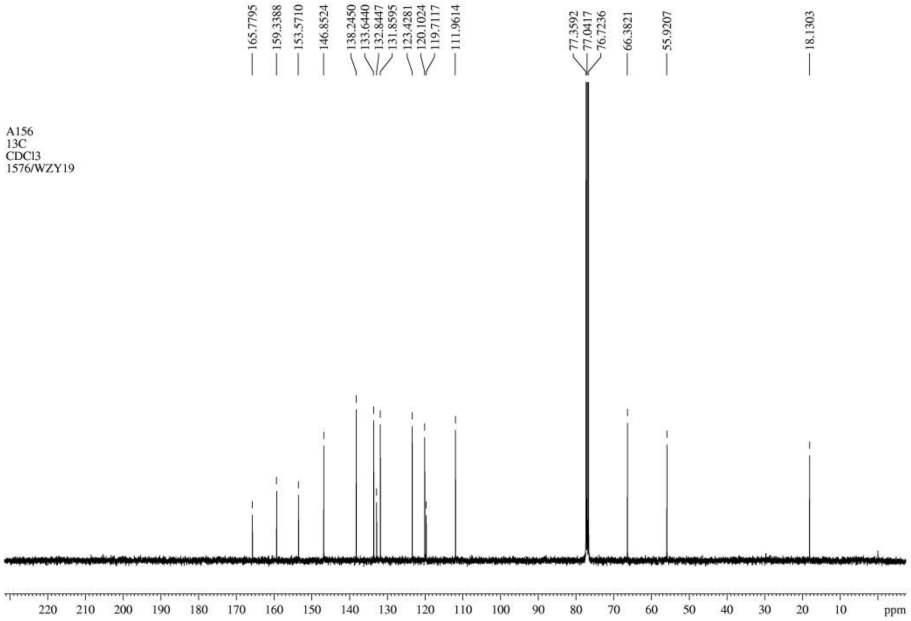

^{13}C NMR spectrum of compound **5e**

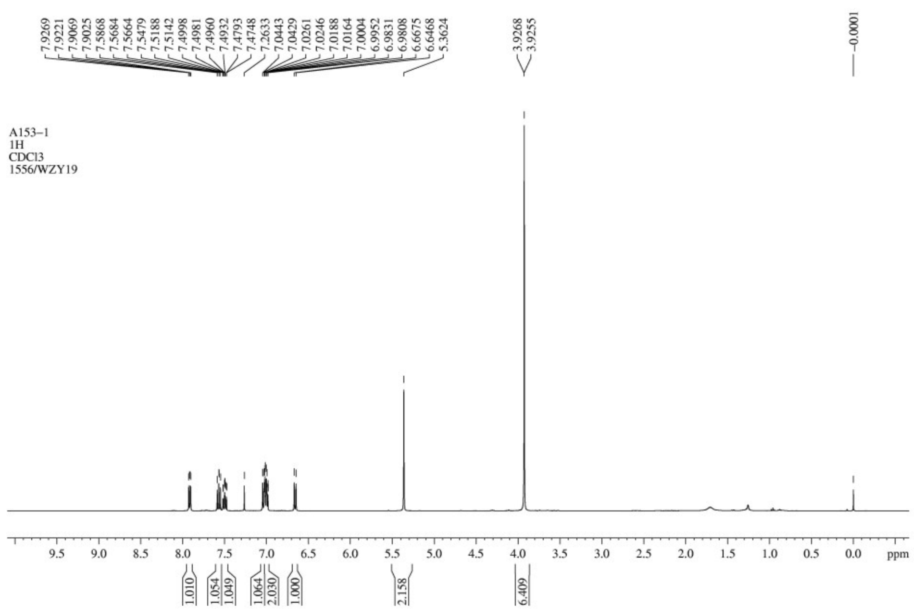

^{1}H NMR spectrum of compound **5f**

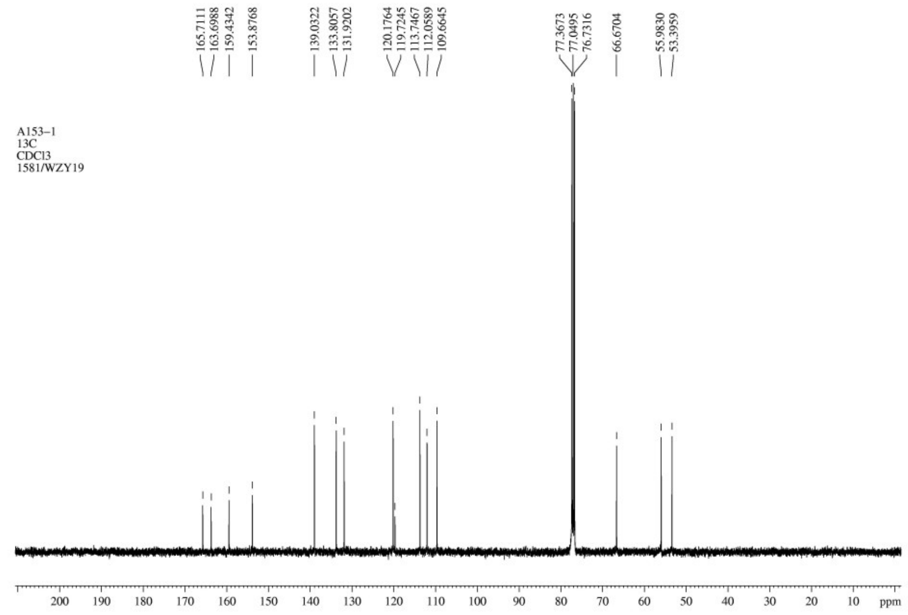

^{13}C NMR spectrum of compound **5f**

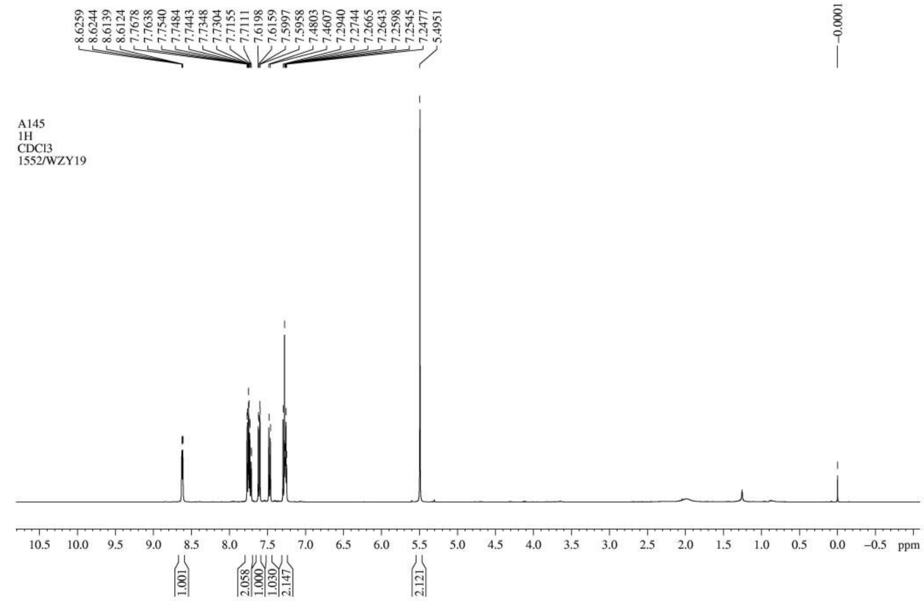

^{1}H NMR spectrum of compound **5g**

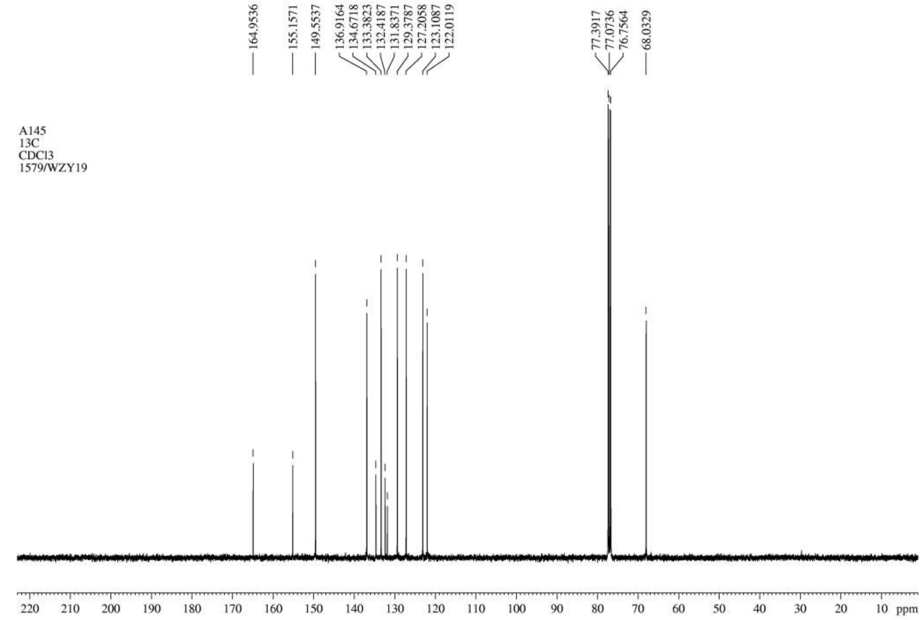

^{13}C NMR spectrum of compound **5g**

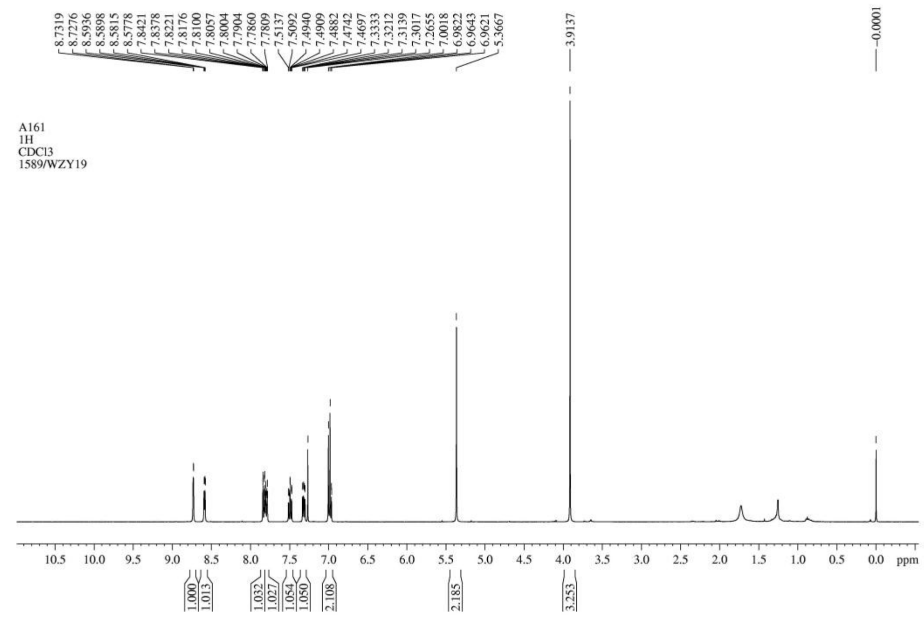

^{1}H NMR spectrum of compound **5h**

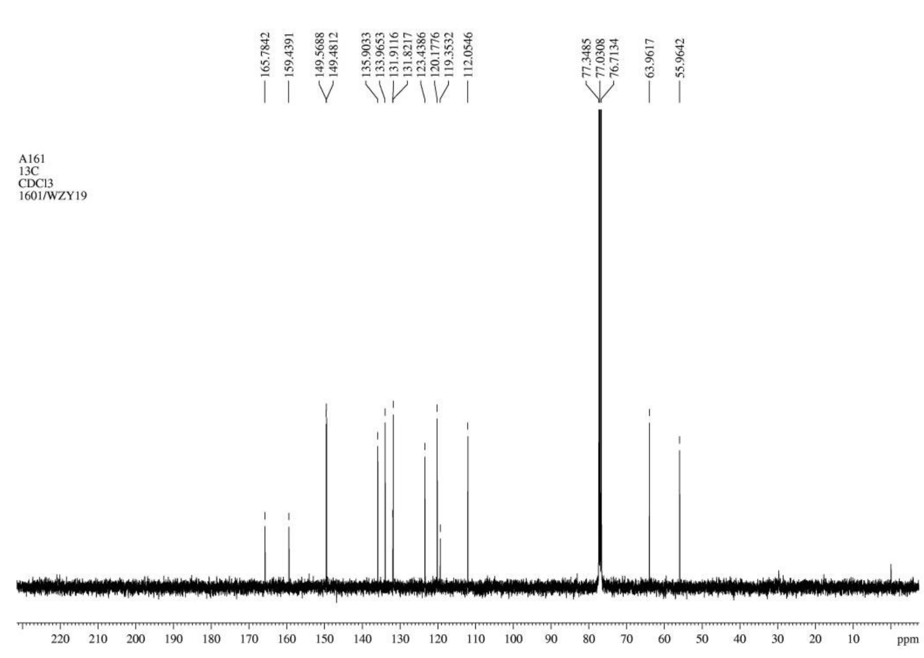

^{13}C NMR spectrum of compound **5h**

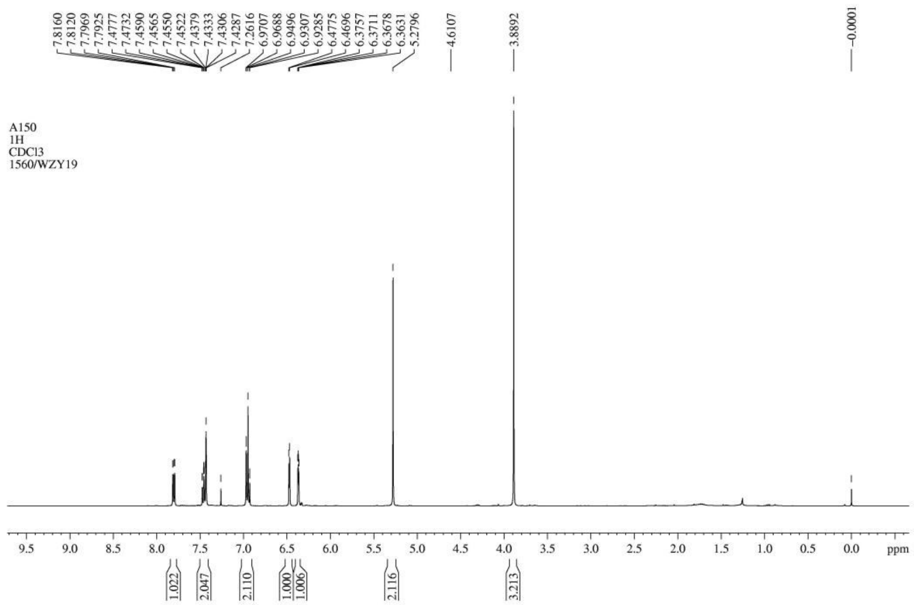

^{1}H NMR spectrum of compound **5i**

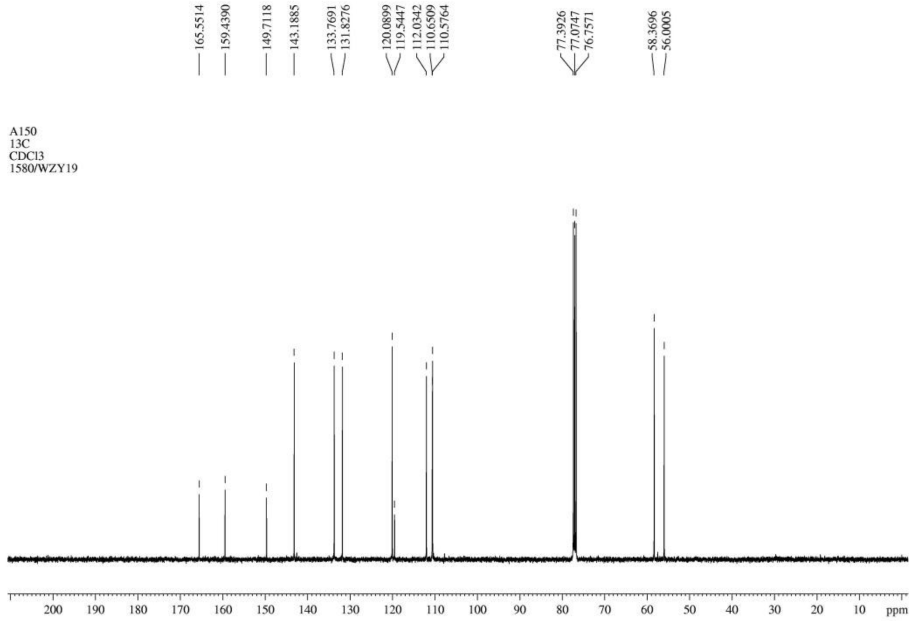

^{13}C NMR spectrum of compound **5i**

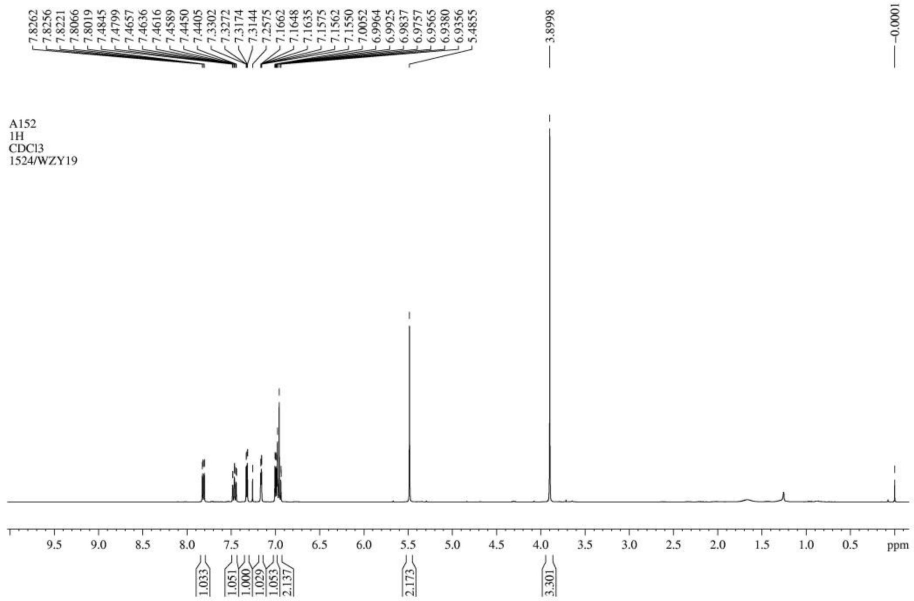

^{1}H NMR spectrum of compound **5j**

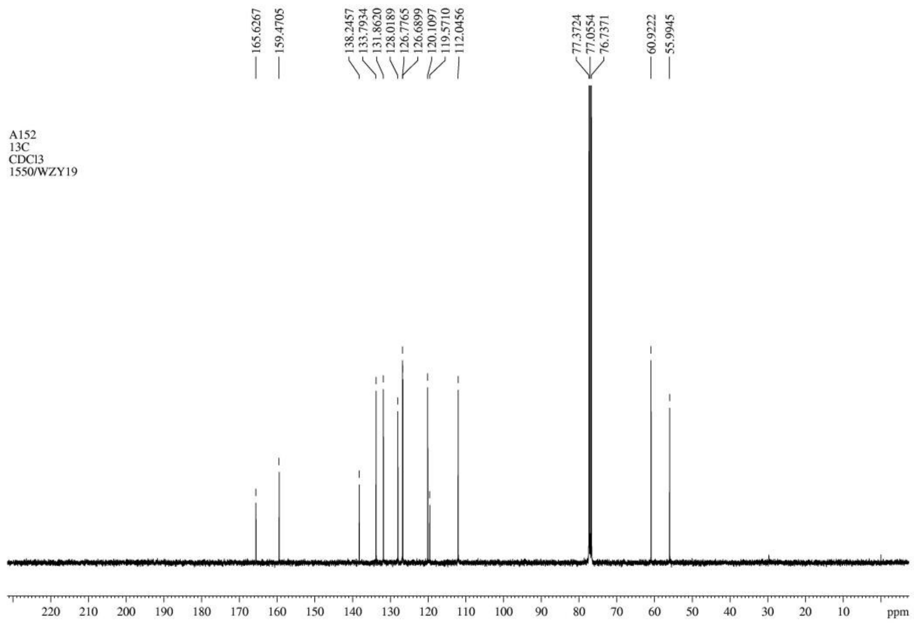

^{13}C NMR spectrum of compound **5j**

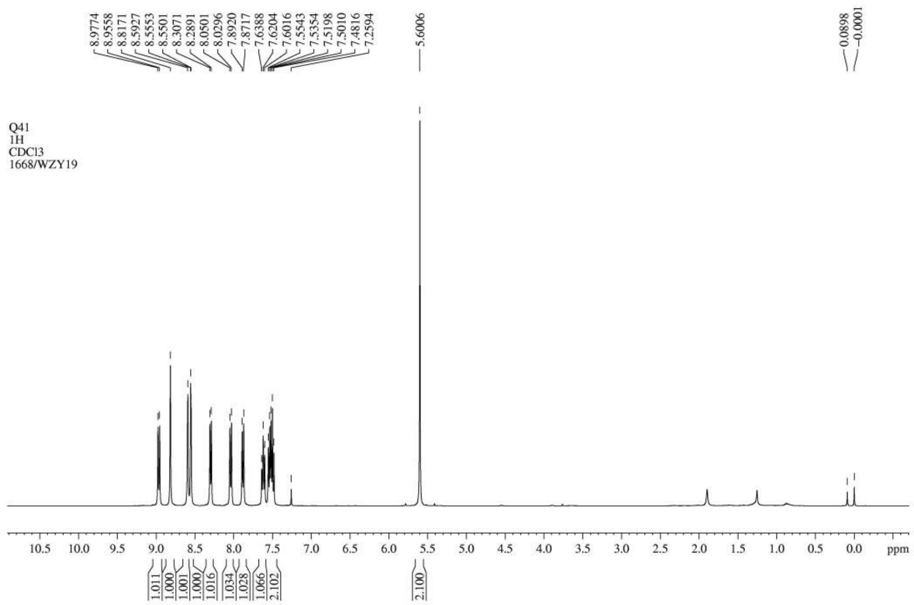

^{1}H NMR spectrum of compound **5k**

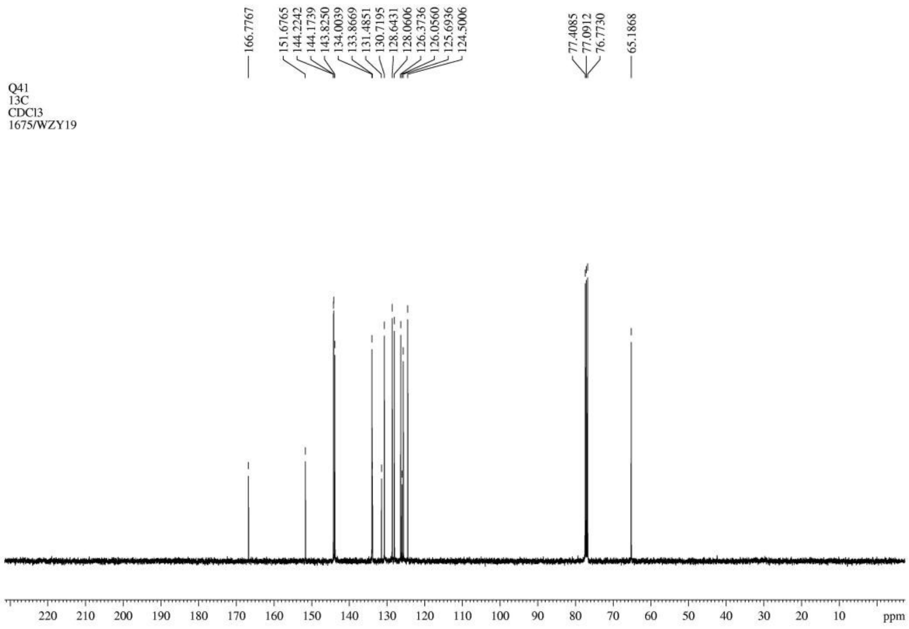

^{13}C NMR spectrum of compound **5k**
